# Humanized APOE genotypes influence lifespan independently of tau aggregation in the P301S mouse model of tauopathy

**DOI:** 10.1186/s40478-023-01581-2

**Published:** 2023-06-19

**Authors:** Tristan Williams, Tim Bathe, Quan Vo, Patricia Sacilotto, Karen McFarland, Alejandra Jolie Ruiz, Gabriela P. Hery, Patrick Sullivan, David R. Borchelt, Stefan Prokop, Paramita Chakrabarty

**Affiliations:** 1 Center for Translational Research in Neurodegenerative Disease, University of Florida, Gainesville, FL 32610 USA; 2 Department of Neuroscience, University of Florida, Gainesville, FL 32610 USA; 3 Department of Neurology, University of Florida, Gainesville, FL 32610 USA; 4 McKnight Brain Institute, University of Florida, Gainesville, FL 32610 USA; 5 Department of Medicine, Duke University, Durham, NC 27710 USA; 6 Department of Pathology, Immunology & Laboratory Medicine, University of Florida, Gainesville, FL 32610 USA; 7 Fixel Institute for Neurological Diseases, University of Florida, Gainesville, FL 32608 USA; 8 Present Address: Eli Lilly & Company, Indianapolis, IN 46285 USA

**Keywords:** APOE, Phosphorylated tau, Aging, Survival, Immune response

## Abstract

Apolipoprotein (APOE) E4 isoform is a major risk factor of Alzheimer’s disease and contributes to metabolic and neuropathological abnormalities during brain aging. To provide insights into whether APOE4 genotype is related to tau-associated neurodegeneration, we have generated human P301S mutant tau transgenic mice (PS19) that carry humanized APOE alleles (APOE2, APOE3 or APOE4). In aging mice that succumbed to paralysis, PS19 mice homozygous for APOE3 had the longest lifespan when compared to APOE4 and APOE2 homozygous mice (APOE3 > APOE4 ~ APOE2). Heterozygous mice with one human APOE and one mouse Apoe allele did not show any variations in lifespan. At end-stage, PS19 mice homozygous for APOE3 and APOE4 showed equivalent levels of phosphorylated tau burden, inflammation levels and ventricular volumes. Compared to these cohorts, PS19 mice homozygous for APOE2 showed lower induction of phosphorylation on selective epitopes, though the effect sizes were small and variable. In spite of this, the APOE2 cohort showed shorter lifespan relative to APOE3 homozygous mice. None of the cohorts accumulated appreciable levels of phosphorylated tau compartmentalized in the insoluble cell fraction. RNAseq analysis showed that the induction of immune gene expression was comparable across all the APOE genotypes in PS19 mice. Notably, the APOE4 homozygous mice showed additional induction of transcripts corresponding to the Alzheimer’s disease-related plaque-induced gene signature. In human Alzheimer’s disease brain tissues, we found no direct correlation between higher burden of phosphorylated tau and APOE4 genotype. As expected, there was a strong correlation between phosphorylated tau burden with amyloid deposition in APOE4-positive Alzheimer’s disease cases. Overall, our results indicate that APOE3 genotype may confer some resilience to tauopathy, while APOE4 and APOE2 may act through multiple pathways to increase the pathogenicity in the context of tauopathy.

**Supplementary Information:**

The online version contains supplementary material available at 10.1186/s40478-023-01581-2.

## Introduction

Both sporadic and familial forms of Alzheimer’s disease (AD) are associated with the presence of the E4 allele of Apolipoprotein E (APOE) [1, 2]. Indeed, relative to the most prevalent E3 allele representing the ‘reference allele’, the E4 allele significantly increases the risk of AD and lowers the age of dementia onset in a gene-dose dependent manner. On the other hand, the E2 allele is associated with increased longevity and protection from AD [3], though presence of E2 allele can often lead to hyperlipoproteinemia and metabolic pathologies [4, 5].

APOE is a lipoprotein that functions as a cholesterol carrier and its three major isoforms—E2, E3 and E4—differ in sequence at two positions [1]. The pathogenic effects of APOE4 in aging and Alzheimer’s dementia is thought to be mediated by its role in various metabolic and immune pathways [6]. APOE4 is associated with higher burden of amyloid β (Aβ) burden [1]. However, there are some divergent datasets regarding the direct involvement of APOE alleles in tau pathogenesis. Previous studies in human tau transgenic PS19 mice showed that APOE4 triggers tau-associated neurodegeneration and unique phosphorylation patterns [7–9], while another report found that APOE2 exacerbates pathologic tau inclusions in a hyper-expression model of tau [10]. In the K18-tau aggregate seeded PS19 mice, we showed that APOE3 homozygosity (E3H) exacerbates the prevalence of hyperphosphorylated tau (phospho-tau) in the early stages of seeded tauopathy (E3H > E4H ~ E2H) [11]. Given these disparate observations, we wanted to examine whether presence of a particular human APOE isoform alters survival and tau pathology in aged PS19 mice. We found that PS19 mice carrying E3 allele (PS/E3H) survived the longest (E3H > E4H ~ E2H) and that the burden of tau pathology was equivalent between PS/E3H and PS/E4H (PS19 mice homozygous for APOE4) mice at end-stage. RNAseq analysis showed that PS/E4H mice showed AD-associated profiles, relative to PS/E3H mice and PS19 mice homozygous for APOE2 (PS/E2H). A survey using limited human AD cases demonstrated that phospho-tau levels were similar between APOE4 and non-APOE4 individuals. Interestingly, phospho-tau burden showed positive correlation with amyloid β (Aβ) deposits in APOE4 individuals, but not in non-APOE4 AD patients. Collectively, our data suggests that APOE3 may influence resilience to tau-associated phenotype in PS19 mice while presence of APOE4 was pathological in the same context.

## Materials and methods

### Mice and study design

Mouse husbandry and experimental procedures were performed in accordance with the protocols and policies approved by the Institutional Animal Care and Use Committee at the University of Florida. All mice were maintained under a 12-h light/dark cycle and had access to water and food ad libitum. The mice were maintained under specific pathogen-free conditions. PS19 mice were obtained from Jackson Labs and maintained on a B6/C3H background as heterozygotes for P301S tau transgene, developing age-progressive hindlimb paralysis at 9–12 months of age [12]. APOE targeted replacement (TR) mice were obtained from Duke University and were maintained as homozygotes on C57BL6 background [13–15]. PS19 mice were mated with APOE homozygote mice to produce N1 cohorts that were heterozygous for APOE. N2 cohorts of PS19 mice that are heterozygous or homozygous for APOE were generated by back-crossing the N1 generation mice with corresponding APOE homozygous mice. APOE and tau levels in these N1 and N2 cohorts were described in a previous study [11]. Cohorts were set up to be aged until the animals reached hindlimb paralysis. At endpoint, all euthanasia was performed with intracardiac perfusion of cold phosphate buffered saline (PBS) containing heparin and the brains were fixed in 10% normal buffered formalin (Fisher Scientific). The fixed brains were then sliced sagitally and processed for paraffin embedding.

### Human brain samples—demographics and APOE genotyping protocol

All brains were obtained from the University of Florida Neuromedicine Human Brain and Tissue Bank. Information on individual patients are summarized in Additional file 6: Table S1.

For APOE genotyping, DNA was extracted from formalin fixed paraffin embedded (FFPE) sections or frozen brain tissue using DNA extraction kits (GeneRead DNA FFPE Kit, Qiagen; and QIAamp DNA Mini Kit, Qiagen). Two single nucleotide polymorphisms (SNPs) within the APOE gene, rs7412 and rs429358, were detected using the Human TaqMan™ SNP Genotyping Assay (Applied Biosystems, Cat. no. 4351376) and TaqMan™ Genotyping Master Mix (Applied Biosystems, Cat. no. 4371355). The relative fluorescent units (RFU) were measured by performing qRT-PCR. The frequencies of APOE alleles and corresponding genotypes were obtained by calculating the ratio and combination of RFUs for both SNPs.

### Immunohistochemical analysis of mouse brain sections

FFPE slides with mouse brain sections were deparaffinized and probed with primary antibodies as described before [11]. For antigen retrieval, slides receiving AT8 (1:1000; Invitrogen MN1020), AT100 (1:1000; Invitrogen MN1060), AT270 (1:1000; Invitrogen MN1050), AT180 (1:1000; Invitrogen MN1040) and glial fibrillary acidic protein (GFAP) (1:3000; Cell Signaling) antibodies were steamed in water at high pressure for 15 min, while Iba-1 (1:2000; Wako) antibody slides were steamed in citrate buffer pH 6.0 (Target Retrieval Solution, Dako). Slides were incubated in 3% hydrogen peroxide (Fisher Scientific) for 20 min to block endogenous peroxidase activity and then washed three times in PBS for 5 min each. Slides were then blocked in 2% fetal bovine serum (FBS) (Hyclone, GE) for 45 min before incubating in primary antibody diluted in block solution overnight at 4 °C. The following day, slides were washed and appropriate secondary antibody (ImmPRESS Polymer Reagent, Vector Labs) was applied for 30 min at room temperature. Following PBS washes, color was developed using 3,3’-diaminobenzidine (Vector DAB, Vector Labs) and slides were counterstained with haematoxylin (Vector Labs). Next, brain sections were dehydrated in a series of ethanol, cleared in xylene, mounted in Cytoseal-60 media (Fisher Scientific) and coverslipped.

### Analysis of histochemical images from mice brain

Immunostained images were captured using a Scanscope XT image scanner (Aperio, Vista, CA). Percent immunoreactivity was computed using the Aperio Positive Pixel Count program (Aperio, Vista, CA). The data is shown as the average %immunoreactivity ± S.E.M. per group. Statistical comparisons were conducted using 1-way ANOVA (GraphPad Prism 7).

### Estimation of ventricular volume

FFPE tissues were sectioned sagittally, starting 1 mm from the midline. Initial slides were stained with haematoxylin and eosin, their location identified using Paxinos and Franklin’s mouse brain atlas and further slides were prepared according to desired bregma locations. 5 µm sections were collected spaced at regular intervals of 25 µm followed by an interval of 250 µm. This pattern was repeated until reaching the target area, which was approximately 3.2 mm from the midline bregma. Slides were stained with haematoxylin and eosin at target area. Assessments of ventricle volume were made at two distances from bregma. One measurement of ventricle volume was made at 2.8–2.76 mm from bregma, and a second measurement was made at 3.2 mm from bregma. Using an Aperio Image Scope application to view the images, the entire perimeter of the lateral ventricles that were visible in the images was outlined and the area of the marked areas were recorded. In more medial sections where the ventricles were discontinuous, the total area of the ventricles was summed.

### Analysis of mouse brains using immunoblotting

Frozen hemibrains were cryo-pulverized and used for tau extraction as detailed earlier [16]. Briefly, brain fractions S1 and S3 containing tau with differential soluble properties were obtained by treating the brain homogenates with a sequential series of buffers with increasing detergent concentrations—Tris buffered saline, high salt buffer containing 10% sucrose and finally 1% sarkosyl solution. The S1 fraction refers to clarified homogenate prepared in Tris buffered saline while S3 fraction refers to the pellet obtained following 1% sarkosyl incubation. The S3 pellet was further solubilized in 4 M urea and 2% SDS and used in western blotting. All steps in the protocol included addition of protease and phosphatase inhibitors (Pierce Protease and Phosphatase Inhibitor Mini Tablets, Thermo Scientific). Immunoblotting methods were followed as described earlier [16]. Membranes were incubated with primary antibodies [Iba-1 (1:250; Novus Biologicals), GFAP (1:1000; Cell Signaling), GAPDH (1:1000; Abcam), AT8 (1:1000; Invitrogen MN1020), AT100 (1:1000; Invitrogen MN1060), AT270 (1:1000; Invitrogen MN1050), AT180 (1:1000; Invitrogen MN1040), PHF1 (1:1000; Peter Davies), total tau (1:1000; Abcam ab254256] and signals detected using multiplex Li-Cor Odyssey Infrared Imaging system (Li-Cor Biosciences, Lincoln, NE). Relative band intensity was quantified using ImageJ software (NIH).

### Analysis of histochemical images from human brain

Human brain tissue sections were received from the UF Neuromedicine Human Brain and Tissue Bank (UF HBTB) following protocols approved by the Institutional Review Board of the University of Florida. Staging of AD neuropathologic changes and associated co-pathologies was conducted per guidelines published by the National Institute on Aging-Alzheimer’s Association [17] by a board-certified neuropathologist (SP). 5 μm thick tissue sections of FFPE brain tissue specimens were deparaffinized and rehydrated. Heat-induced epitope retrieval was performed in a pressure cooker (Tintoretriever, Bio SB) for 15 min at high pressure in a 0.05% Tween-20 solution. Endogenous peroxidase was quenched by incubation of sections in 1.5% hydrogen peroxide/0.005% Triton-X-100 diluted in sterile PBS (Invitrogen) for 20 min, following multiple washes in tap water and subsequently, 0.1 M Tris, pH 7.6. Non-specific antibody binding was minimized with sections incubated in 2% FBS/0.1 M Tris, pH 7.6. Primary antibodies were diluted in 2% FBS/0.1 M Tris, pH 7.6 (anti-Aβ antibody Ab5 [Todd E. Golde], 1:1000; anti-tau (AT8), 1:5000, invitrogen MN1020; anti-α-synuclein (94-3A10) [Benoit I. Giasson], 1:10,000). Sections were incubated with primary antibody over night at 4 °C, washed one time in 0.1 M Tris, pH 7.6, followed by 2% FBS/0.1 M Tris, pH 7.6 for 5 min, incubated in goat anti-rabbit IgG horseradish peroxidase (HRP) conjugated secondary antibody (Millipore Sigma) for 1 h, additionally washed one time in 0.1 M Tris, pH 7.6, followed by 2% FBS/0.1 M Tris, pH 7.6 for 5 min, and incubated in VectaStain ABC Peroxidase HRP Kit (diluted in 2% FBS/0.1 M Tris, pH 7.6 at 1:1000) for 1 h. After a final wash in 0.1 M Tris, pH 7.6 for 5 min, immunocomplexes were visualized using 3,3′-diaminobenzidine (DAB). Tissue sections were counterstained with hematoxylin (Sigma Aldrich, St. Louis) for 2 min, dehydrated and cover slipped using Cytoseal 60 mounting medium (Thermo Scientific).

For analysis of stains, slides were scanned on an Aperio AT2 scanner (Leica biosystems) at 20 × magnification. Digital slides were analyzed using the QuPath platform (version 0.3.2) [18]. Cortex and white matter were annotated for regional analysis. After exclusion of tissue and staining artifacts, the ‘Positive Pixel Detection’ tool (e.g., Downsample factor 2, Gaussian sigma 1 μm, Hematoxylin threshold (‘Negative’) 1.5 OD units, DAB threshold (‘Positive’) 0.2 OD units) was used to determine the percentage of area covered by pathological staining. ‘Positive Pixel Detection’ thresholds were adjusted for individual stains.

### RNA isolation from frozen brains and bulk Rnaseq analysis

The number of samples were 3 mice each for parental E2H, E3H and E4H cohorts and 4 mice each for PS/E2H, PS/E3H and PS/E4H cohorts. All mice were male, except 1 female for E4H, 1 female for PS/E2H and 2 females for PS/E4H cohort. RNA was extracted from frozen, pulverized forebrains using TRIzol reagent (Invitrogen). Extracted RNA was cleaned using RNeasy mini extraction kit with an on-column DNase treatment (Qiagen). RNA quality was quantified with the Qubit RNA HS assay (Invitrogen) and transferred to LC Sciences (Houston, TX) for library construction and Rnaseq. Poly(A) RNA sequencing library was prepared following Illumina’s TruSeq-stranded-mRNA sample preparation protocol. RNA integrity was checked with Agilent Technologies 2100 Bioanalyzer. Poly(A) tail-containing mRNAs were purified using oligo-(dT) magnetic beads with two rounds of purification. After purification, poly(A) RNA was fragmented using divalent cation buffer in elevated temperature. Quality control analysis and quantification of the sequencing library were performed using Agilent Technologies 2100 Bioanalyzer High Sensitivity DNA Chip. Paired-ended sequencing was performed on Illumina’s NovaSeq 6000 sequencing system. Firstly, Cutadapt [19] and perl scripts in house were used to remove the reads that contained adaptor contamination, low quality bases and undetermined bases. Then sequence quality was verified using FastQC (http://www.bioinformatics.babraham.ac.uk/projects/fastqc/). We used HISAT2 [20] to map reads to the genome of ftp://ftp.ensembl.org/pub/release-101/fasta/mus_musculus/dna/. The mapped reads of each sample were assembled using StringTie [21]. Then, all transcriptomes were merged to reconstruct a comprehensive transcriptome using perl scripts and gffcompare. After the final transcriptome was generated, StringTie [21]] and ballgown (http://www.bioconductor.org/packages/release/bioc/html/ballgown.html) was used to estimate the expression levels of all transcripts. StringTie [21] was used to perform expression level for mRNAs by calculating FPKM (fragments per kilobase of exon per million mapped fragments). mRNAs differential expression analysis were performed by R package DESeq2 [22] between two different groups (and by R package edgeR [23] between two samples). The mRNAs with the parameter of false discovery rate (FDR) below 0.05 and absolute fold change ≥ 2 were considered differentially expressed mRNAs. Gene Set Enrichment Analysis (GSEA) with GO and KEGG databases were done using gene sets with |NES|≥ 1 & NOM *p*-value < 0.05 & FDR q-value < 0.25, where NES = normalized enrichment score, NOM = NOM and FDR = false discovery rate.

### Cell profile and microglial profile analysis

Gene lists identifying cell-types within the brain were described previously [24–30]. The geometric mean of the FPKM for genes identified for each cell or cellular sub-type was calculated from each animal. Group means were calculated, and between group significance values determined by 1-way ANOVA. Outliers were removed from the group if their value fell outside of 1.5 times the inter-quartile range (IQR).

### Statistics

Immunohistochemical data was analyzed using 1-way ANOVA unless otherwise indicated in figure legend. Outliers, if applicable, were removed using Rout’s test with Q = 1%. The neuropathological scoring was done in a blinded manner. All data was assembled using Adobe Photoshop Elements.

## Results

### APOE isoform dependent survival in PS19 mice homozygous for APOE

**Fig. 1:**
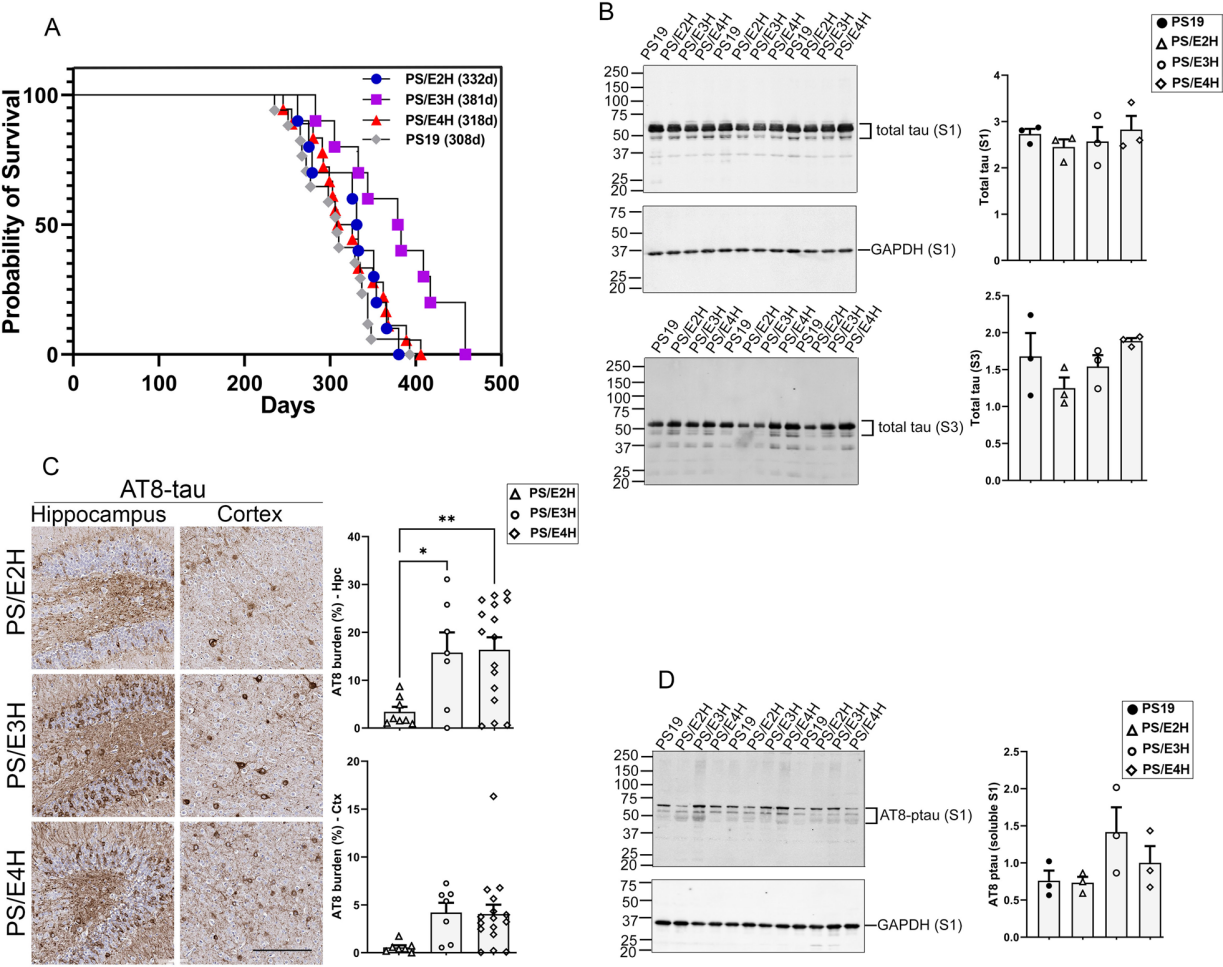
APOE isoform influences survival and tauopathy burden in PS19 mice. PS19 mice homozygous for APOE allele were aged until they exhibited bilateral hindlimb paralysis. **A** The percentage of surviving mice was plotted against survival days for each APOE genotype (blue circle = PS/E2H; purple square = PS/E3H; red triangle = PS/E4H; grey diamond = PS19). n = 10 mice/genotype (PS/E2H, PS/E3H), 18 mice (PS/E4H) and 16 mice (PS19). Log-rank (Mantel-Cox) test. **B** Immunoblotting of total tau was done in detergent soluble (S1) and sarkosyl-insoluble (S3 pellet) brain fractions derived from paralyzed mice. Band intensity of S1-total tau was normalized to GAPDH, whereas band intensity of S3-total tau was normalized to the total protein concentration of the brain homogenates. Numerals on the left side of each blot denote molecular weight standards in kDa. n = 3 mice/genotype. **C** Paralyzed PS/E2H, PS/E3H and PS/E4H mice were analyzed for phospho-tau levels using AT8 antibody. Representative immuno-stained images and corresponding quantification of AT8 immunostaining (% immunoreactivity) in the cortex and hippocampus are shown. n = 9 (PS/E2H), 7 (PS/E3H), 16 (PS/E4H).1-way ANOVA *p < 0.05, **p < 0.01. Scale bar: 100 µm. **D** Immunoblotting of AT8-tau was done in detergent soluble (S1) brain fraction derived from paralyzed mice. Band intensity of S1-total tau was normalized to GAPDH. Numerals on the left side of each blot denote molecular weight standards in kDa. n = 3 mice/genotype. 1-way Anova

As described earlier [11], mice carrying humanized APOE alleles [13–15] were bred with P301S tau transgenic Line PS19 mice [12] to generate tau transgenic mice that are heterozygous for APOE (PS/E2h, PS/E3h and PS/E4h) or homozygous (PS/E2H, PS/E3H and PS/E4H) for human APOE. PS19 mice show progressive paralysis as a result of accumulation of tau pathology in the spinal cord and brain [12]. In our colony, the median age to humane endpoint of bilateral hindlimb paralysis for PS19 mice on the B6/C3H background, carrying mouse Apoe, is 308 days (n = 7 males, 9 females) (Fig. 1A). We examined whether the presence of specific human APOE or the gene dosage of APOE influenced lifespan in PS19 mice (Fig. 1A). The median number of days to humane endpoint of bilateral hindlimb paralysis for PS/E2H, PS/E3H and PS/E4H mice was 332, 381 and 318 days, respectively (Fig. 1A). The longer average survival for PS/E3H mice was statistically different from PS/E2H mice (*p* = 0.015), PS/E4H mice (*p* = 0.008) and PS19 mice (*p* = 0.003). There was no statistical difference in median age to paralysis between PS/E2H, PS/E4H and PS19 cohorts. Previous studies, using coronal brain sections of PS19 crossed with humanized APOE mice, showed that APOE4 exacerbates ventricular enlargement [7]. In our study, we used sagittal sections from two selected brain areas, one a medial and another a lateral location with respect to midline to explore changes in ventricular volume. In our cohorts of PS19xAPOE mice, we observed high variability in ventricular volumes and did find any statistically significant differences in ventricular volume between the three genotypes (Additional file 1: Fig. S1A, B).

### Phosphorylated tau burden in PS19 mice homozygous for APOE

We investigated the levels of tau and phospho-tau in brains of these paralyzed mouse cohorts (Fig. 1, Additional file 2: Fig. S2). Using an antibody against total tau, we observed that all of our mouse lines showed comparable tau levels in both the detergent soluble (S1) and detergent insoluble (S3) fractions (Fig. 1B). Using AT8 (pSer202/Thr205) antibody staining in brain sections, we observed lower levels of phospho-tau immunoreactivity in PS/E2H mice compared to PS/E3H (*p* < 0.05) and PS/E4H (*p* < 0.01) mice in the hippocampus (Fig. 1C). Cortical AT8 immunoreactivity was somewhat lower in PS/E2H mice compared to PS/E3H and PS/E4H mice, though this did not reach statistical significance due to high variability (Fig. 1C). Immunoblotting of soluble and detergent-insoluble fractions from hemibrains showed that AT8-immunopositive detergent-soluble tau was equivalent between the three APOE genotypes (Fig. 1D). Notably, we failed to detect insoluble tau showing AT8-site phosphorylation by immunoblotting in these mice (Additional file 2: Fig. S2G). These findings suggest that most of the AT8 immunoreactivity seen in the cortex of PS19 mice is from phosphorylated soluble tau.

Next, we investigated whether other tau phosphorylation epitopes showed any differential association with APOE genotypes. Immunohistochemistry analysis of pThr212/Ser214 (AT100), pThr181 (AT270) and pThr231 (AT180) burden showed equivalent levels between PS/E2H, PS/E3H and PS/E4H mice (Additional file 2: Fig. S2A-B). Immunoblotting of soluble and detergent-insoluble fractions with these antibodies showed variable patterns of immunoreactivity (Additional file 2: Fig. S2C–G). Consistent with the immunohistological staining, the level of AT100 immunostaining in soluble fractions was low (Additional file 2: Fig. S2C). In the limited samples we analyzed here, soluble AT100 levels were somewhat lower in PS/E2H mice as compared to other genotypes (Additional file 2: Fig. S2C; *p* < 0.05 relative to PS/E3H and PS/E4H). Robust levels of AT270 and AT180 immunoreactivity were detected in soluble fractions from all genotypes (Additional file 2: Fig. S2D, E). We also analyzed biochemical levels of PHF1 (Ser396/404), finding robust staining in soluble fractions as well as detectable amounts in detergent-insoluble fractions (Additional file 2: Fig. S2F). In the detergent-insoluble fractions, AT100-, AT180- and AT8-positive phospho-tau were undetectable (Additional file 2: Fig. S2C, E, G). Presence of total tau on these same blots confirmed that these phospho-tau epitopes were specifically not induced. Levels of silver-positive Gallyas staining showed high variability in forebrains of these mice and thus were not considered for analysis (data not shown). Collectively, these data suggest that most of the cortical phospho-tau present in the brains of these mice was present as detergent soluble tau. Since our data shows that PS/E3H and PS/E4H mice had equivalent phospho-tau pathology, but PS/E4H mice reached end-stage (hindlimb paralysis) earlier than PS/E3H mice, this suggests that the E3 allele imparts some resilience to tau-mediated pathogenesis.

### Gliosis in PS19 mice homozygous for APOE

**Fig. 2:**
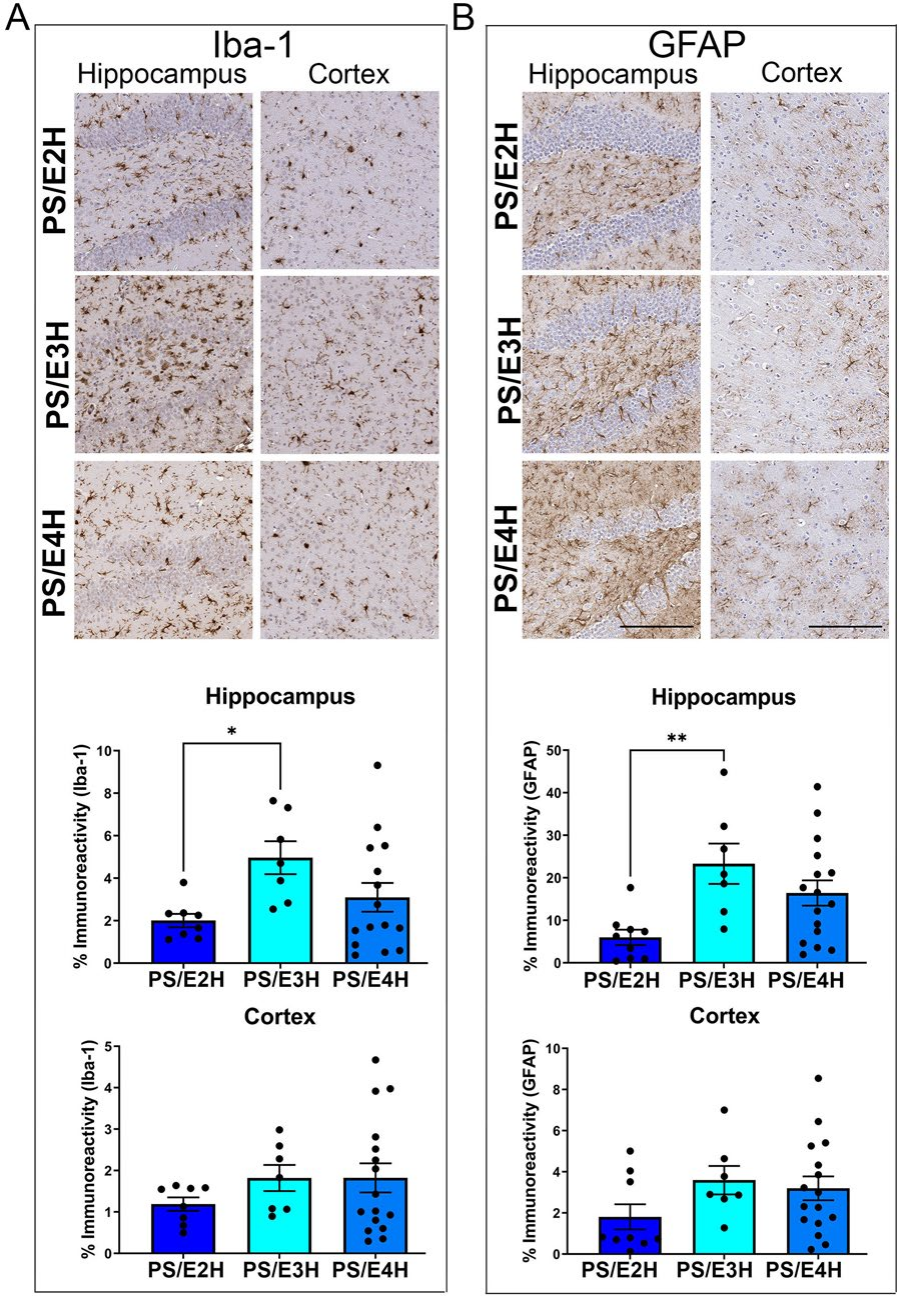
Gliosis levels in PS19 mice homozygous for APOE2, APOE3 or APOE4. PS19 mice homozygous for APOE allele were aged until they exhibited bilateral hindlimb paralysis and analyzed for microgliosis (**A**) and astrocytosis (**B**) in the forebrain. Representative immuno-stained images and corresponding quantification of Iba-1 and GFAP immunostaining (% immunoreactivity) in the cortex and hippocampus are shown. (n = 9 (PS/E2H), 7 (PS/E3H), 16 (PS/E4H).1-way ANOVA *p < 0.05, **p < 0.01. Scale bar: 100 µm

We examined hippocampal and cortical microgliosis and astrogliosis in PS19 mice carrying different APOE alleles, using Iba-1 and GFAP antibodies respectively. Hippocampal burden of Iba-1 immunoreactivity was somewhat lower in PS/E2H mice compared to PS/E3H and PS/E4H mice (PS/E2H vs PS/E3H: *p* < 0.05), with the latter two genotypes showing equivalent levels (Fig. 2A). There was no difference in cortical microglia staining in the three cohorts (Fig. 2A). Immunoblotting analyses of Iba-1 levels from the whole forebrain showed equivalent levels among the three APOE genotypes of PS19 mice (Additional file 3: Fig. S3A), indicating region-specific microgliosis in these mice.

We observed lower hippocampal burden of GFAP in PS/E2H mice compared to PS/E3H mice (*p* < 0.01), with no difference observed between PS/E3H and PS/E4H mice (Fig. 2B). There was no difference in cortical astroglia staining among the three cohorts of bigenic mice (Fig. 2B). GFAP immunoblotting showed similar levels in PS19 mice bearing mouse Apoe or human APOE2, APOE3 or APOE4 (Additional file 3: Fig. S3B). Notably, the PS/E3H bigenic mice showed higher astrogliosis compared to the corresponding parental E3H mice (Additional file 3: Fig. S3B; *p* < 0.05), while the other group comparisons (PS/E2H vs E2H and PS/E4H vs E4H) did not reach statistical significance. Our results indicate that presence of the tau transgene induces variable levels of astrogliosis in all parental APOE genotypes, and most consistently in E3H mice.

### Survival, tau burden and gliosis in PS19 mice heterozygous for APOE

We investigated the lifespan of PS19 mice carrying one human APOE allele and one endogenous Apoe allele. In these heterozygous APOE mice, we did not observe any differential time to paralysis based on APOE genotype. The median ages of survival of the PS/E2h, PS/E3h and PS/E4h mice were 358, 325 and 365 days respectively (Additional file 4: Fig. S4A, B). Interestingly, the PS/E2h mice had a longer lifespan compared to its APOE homozygous counterpart, PS/E2H mice (*p* = 0.028) (Additional file 4: Fig. S4B), showing the detrimental influence of increased APOE2 gene dose on age to paralysis. Likewise, the PS/E4h mice had a longer lifespan compared to PS/E4H mice (*p* = 0.013) (Additional file 4: Fig. S4B). Although the median age to paralysis for PS/E3h mice was substantially shorter than that of PS/E3H mice (325 vs 381 days), the difference was not statistically significant. Within the APOE heterozygous PS19 cohorts, we did not observe any statistically significant changes in the cortical or hippocampal burden of phospho-tau or Iba-1 (Additional file 4: Fig. S4C, D). Cortical (but not hippocampal) levels of GFAP was lower in PS/E2h mice compared to PS/E3h mice (*p* < 0.05), with no difference noted between PS/E3h and PS/E4h mice (Additional file 4: Fig. S4E). Whether this difference is biologically meaningful is uncertain, given the high variability in reactivity within these cohorts.

### RNAseq reveals APOE isoform-based differences in PS19 mice

**Fig. 3:**
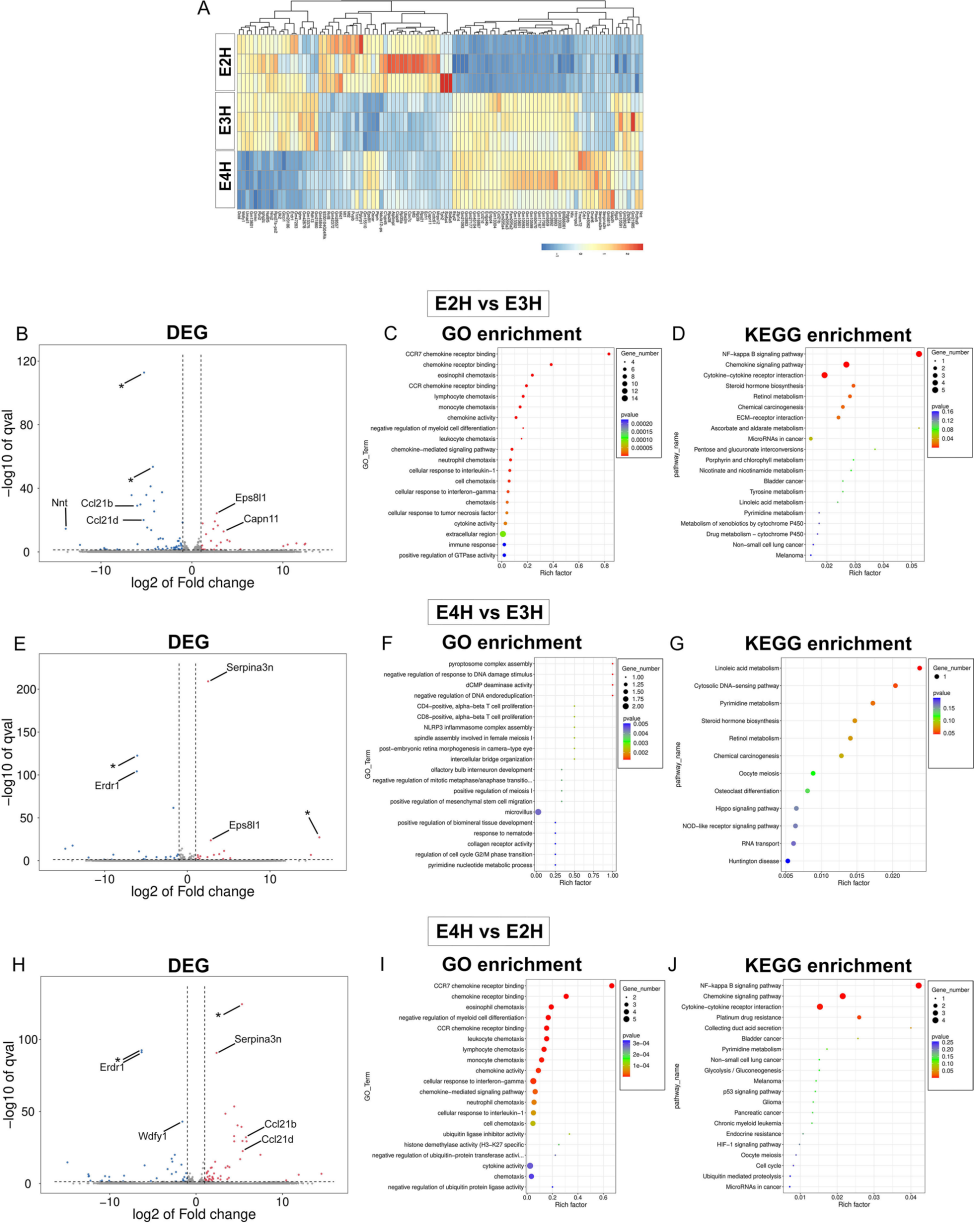
Bulk RNAseq analysis of the forebrains of APOE homozygous mice. Differentially regulated genes from APOE2, APOE3 and APOE4 homozygous mice are presented in a heat map (**A**). Volcano plot of DEG and GO and KEGG pathway analysis of enriched gene sets are shown for E2H vs E3H mice (**B**–**D**), E4H vs E3H mice (**E**–**G**), and E4H vs E2H mice (**H**–**J**). Asterisks in volcano plot refer to predicted genes. The bubble plots are colored by *p*-value and sized by number of genes in enriched gene sets, as defined in the accompanying panel. N = 3 mice/group

To illuminate the potential mechanisms by which APOE could influence the neuropathology in PS19 mice, we conducted bulk RNAseq analysis of the forebrains of PS19 mice homozygous for human APOE and corresponding parental APOE homozygous cohorts (Additional file 6: Table S2). Comparison of the three parental APOE homozygous cohorts showed clear APOE genotype-based differences in gene expression (Fig. 3; Additional file 6: Table S2A-C). Relative to E3H mice, the chemokine genes, Ccl21b and Ccl21d, were downregulated and the cysteine protease, Capn11, was upregulated in E2H mice. Gene Ontology (GO) enrichment analysis showed that multiple GO terms associated with immune function (chemokine receptor binding, chemotaxis and cellular response to cytokines) were differentially altered in E2H mice. Analysis of KEGG pathways also showed consistent enrichment in immune pathways in E2H mice relative to E3H mice (Fig. 3B–D). Fewer differences were observed between E4H and E3H mice at this age. Notable genes that were upregulated in E4H mice (relative to E3H mice) were Serpina3n (cysteine protease inhibitor) and Eps8l1 (signaling adaptor). Enrichment of GO terms and KEGG pathways showed few significant events – most of which were related to metabolic pathways and cellular response to DNA damage (Fig. 3F–G). Comparison of E4H to E2H revealed several immune genes upregulated in E4H mice, such as Ccl121b and Ccl21d (Fig. 3H). The GO term and KEGG pathway profiles in E4H vs E2H comparison were similar to E2H vs E3H comparison, with immune pathways identified as enriched in both the analyses (Fig. 3I, J; C, D). Notably, while immune genes were downregulated in E2H mice (relative to E3H mice), these genes were upregulated in E4H mice (relative to E2H mice).

There were substantial differences in PS19 mice carrying the three APOE alleles (Additional file 5: Fig. S5A). Genes that were differentially upregulated in the comparisons of parental APOE lines were also found in the PS19 tau transgenics carrying the corresponding APOE allele (examples include Eps8l1 in E2H vs E3H, Eps8l1 in E4H vs E3H and Ccl21d in E4H vs E2H comparisons) (Fig. 3B, E, H vs Additional file 5: Fig. S5 B, C, D; Additional file 6: Table S2D-F). We normalized the within-group differences to gain insights into the differential gene expression (DEG) patterns that are determined by APOE genotype or presence of tau transgene (Additional file 5: Fig. S5E–H). The common DEG list showed few genes that are altered and few common pathways were found to be enriched when we investigated differences between E2H vs E3H mice or E4H vs E3H mice, after allowing for the effect of PS19 genotype to be normalized (Additional file 5: Fig. S5E, G). For example, relative to APOE3, NADP + transhydrogenase activity and myeloid cell chemotaxis were downregulated in APOE2 genotypes, whereas no upregulated pathways were identified in this comparison. On the other hand, relative to APOE3, dUMP metabolic process was down regulated and translational fidelity pathway was upregulated in APOE4 mice. We further tested for the effect of the PS19 genotype, after normalizing for the differential contribution of APOE. The presence of tau transgene led to upregulation of a larger number of genes in these mice, with 127 genes commonly upregulated in E2H vs E3H comparison and 252 genes upregulated in E4H vs E3H comparison (Additional file 5: Fig. S5F–H). Expression of tau transgene leads to a robust immune response and stress response that are upregulated in both APOE2 (relative to APOE3) and APOE4 (relative to APOE3) genotypes (Additional file 5: Fig. S5F–H). This suggests that tau expression imparts an overwhelming immune phenotype in these APOE homozygous mice. There were fewer genes that were commonly downregulated in these comparisons (Additional file 5: Fig. S5F–H), one being the keratinocyte activation pathway that was downregulated in both these DEGs (E2H vs E3H and E4H vs E3H).

### Tau transgene induced DEG in APOE4 and APOE3 homozygous PS19 mice

**Fig. 4:**
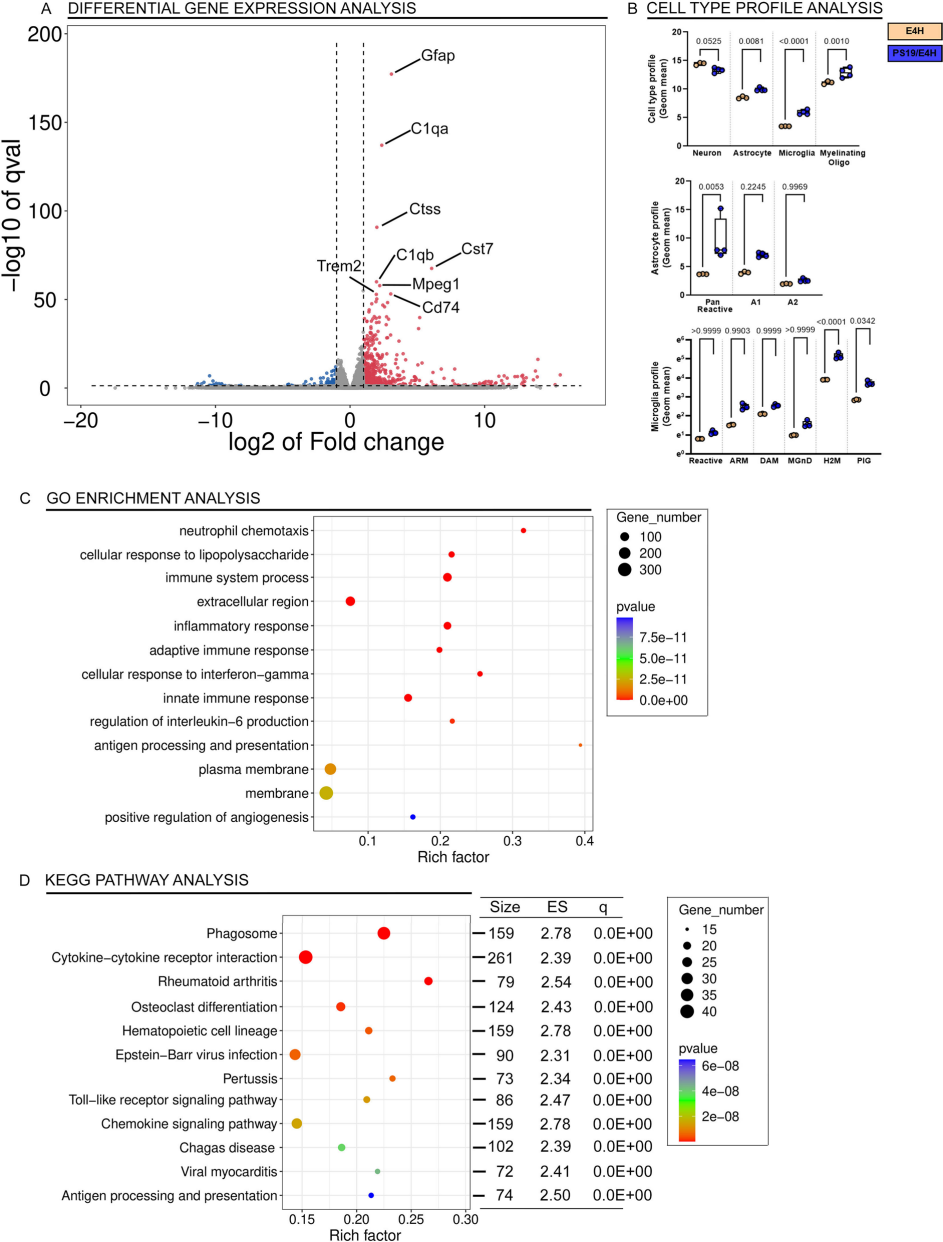
Unbiased transcriptomic analysis of PS19 mice homozygous for APOE4 shows predominant immune signature. Brains of paralyzed PS/E4H mice and 9–10-month-old E4H mice were analyzed by RNAseq. Genes (**A**), cell types (**B**), astrocyte gene expression profiles (**B**) and aging/AD-associated profiles (**B**) differentially regulated in PS/E4H mice relative to E4H mice is shown. *p* values adjusted for multiple testing; FDR = 0.05. 1-way Anova. qval denotes false discovery rate. The enriched up- and down-regulated genes were plotted onto pathways using the GO database (**C**) and KEGG pathway database (**D**) and represented in bubble plots. Pathways with an over-represented *p*-value ≤ 0.05, the number of module genes within the pathway > 15 and an enrichment score > 1.5 are depicted. The bubble plots are colored by *p*-value and sized by the enrichment score. Size, number of genes within each module; ES, Enrichment Score; q value, false discovery rate. N = 4 mice for PS/E4H and 3 mice for E4H

We wanted to compare the individual DEG in PS19 mice within each APOE genotype to understand how tau transgene expression is related to eventual tau-mediated neurodegeneration phenotype. Comparing the PS/E4H to E4H mice, we noted that 216 genes were upregulated while 40 genes were down regulated (Fig. 4A; Additional file 6: Table S2G). Genes corresponding to glial activation, such as Gfap, C1qa, C1qb and Mpeg1, were highly upregulated (Fig. 4A). Notable genes that have been reported to be part of the APOE-associated pathogenic signature in AD, Trem2 (↑3.7x, *p* = 1.46E−22) and Clec7A (↑36.2x, *p* = 3.1E−25), were upregulated in PS/E4H mice compared to E4H mice. APOE, Trem1 and Treml1 remained unchanged, but Treml2 was upregulated (↑4.7x, *p* = 4.22E−05). We also examined the cell types most perturbed in these mice. There was a slight decrease in neuronal gene expression (*p* = 0.052) and robust increase in glial genes (astrocyte, *p* = 0.0081; microglia, *p* < 0.0001; oligodendrocyte, *p* = 0.001) (Fig. 4B). We further examined whether a specific AD-associated astrocyte profile could be correlated with the early death phenotype. We found that pan-reactive astrocyte profile was upregulated (*p* = 0.0053), consistent with Gfap upregulation, but neither A1 or A2 astrocyte profiles were specifically induced. Among other aging/AD-associated gene expression signatures identified from human brains, we could identify plaque induced gene (PIG, *p* = 0.0342) and the H2M (*p* < 0.0001) homeostatic microglia signatures in the PS/E4H mice (Fig. 4B). The upward trends in microglia neurodegenerative signatures (MGnD and DAM) did not survive multiple testing correction (Fig. 4B). We performed pathway analysis using enriched gene sets. Using the GO and KEGG databases, we identified immune processes as the main pathways emerging in the PS/E4H vs E4H comparison. Examples of enriched pathways in PS/E4H mice include Il-6 and Ifn-γ signaling, antigen processing and presentation, Toll-like receptor and chemokine signaling pathway (Fig. 4C, D).

**Fig. 5:**
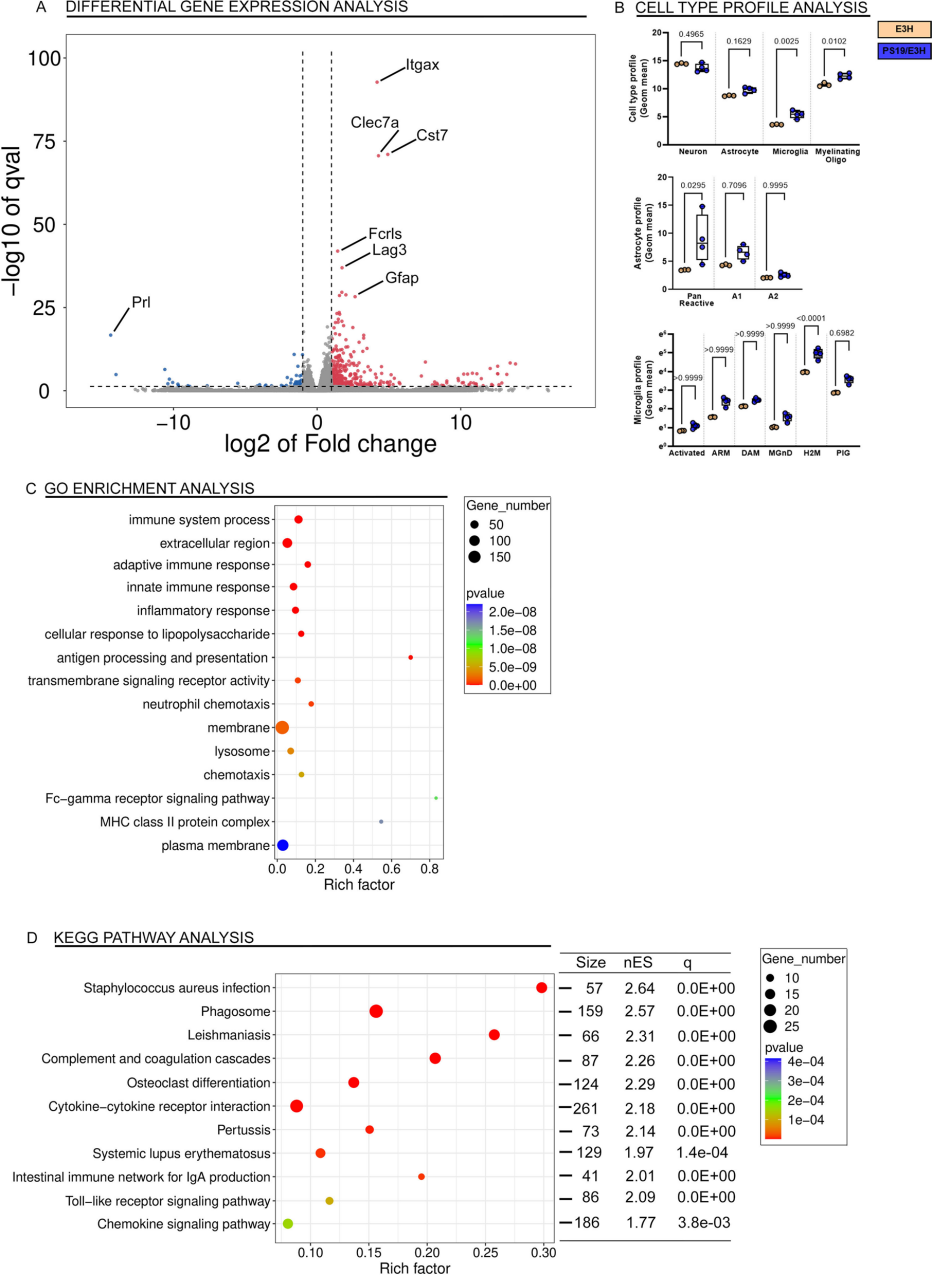
Unbiased transcriptomic analysis of PS19 mice homozygous for APOE3. The forebrains of paralyzed PS/E3H mice and 9–10-month-old E3H mice were analyzed by RNAseq. Genes (**A**), cell types (**B**), astrocyte gene expression profiles (**B**) and aging/AD-associated microglia profiles (**B**) differentially regulated in PS/E3H mice relative to E3H mice is shown. *p* values adjusted for multiple testing; FDR = 0.05. 1-way Anova. Q value denotes false discovery rate. The enriched up- and down-regulated genes were plotted onto pathways using the GO database (**C**) and KEGG pathway database (**D**) and represented in bubble plots. Pathways with an over-represented *p*-value ≤ 0.05, the number of module genes within the pathway > 15 and an enrichment score > 1.5 are depicted. The bubble plots are colored by *p*-value and sized by the enrichment score. Size, number of genes within each module; ES, Enrichment Score; q value, false discovery rate. N = 4 mice for PS/E3H and 3 mice for E3H

Compared to age-matched E3H mice, the PS/E3H mice showed 155 transcripts upregulated and 22 transcripts downregulated (Fig. 5A, Additional file 6: Table S2H). Mostly immune system mediators, such as Itgax, Fcrls, Lag3 and Gfap were upregulated in the PS/E3H mice relative to age-matched E3H mice (Fig. 5A). Similar to changes observed in PS/E4H cohorts, Trem2 (↑2.8x, *p* = 6.32E−13) and Clec7A (↑19.5x, *p* = 7.4E−18) were also upregulated in PS/E3H mice compared to E3H mice, though the strength of association (based on fold change and *p* values) was lower in the in PS/E3H mice. APOE, Trem1 and Treml1 levels also remained similar between PS/E3H and E3H mice, while Treml2 was upregulated (↑4.1x, *p* = 8.7E−04). The cell types that were most perturbed in the PS/E3H mice were microglia (*p* = 0.0025) and oligodendrocytes (*p* = 0.0102) (Fig. 5B). Similar to PS/E4H vs E4H comparison, we observed global changes in astrocytic gene expression (*p* = 0.0295) in PS/E3H mice, though the A1/A2 markers were not changed (Fig. 5B). Among the aging/AD-associated gene expression profiles examined in the PS/E3H vs E3H comparison, only the H2M signature was upregulated (*p* < 0.0001), with no changes in ARM, DAM, MGnD and PIG (Fig. 5B).

As there was a distinct difference in lifespan of the PS/E4H and PS/E3H mice, we were curious whether DEG analysis between these two cohorts could provide insights into tau-mediated pathological effects in E4H vs E3H background. A total of 45 genes were significantly altered, with 14 genes upregulated in PS/E4H mice (Additional file 5: Fig. S5C; Additional file 6: Table S2E). Analysis of the enriched gene sets revealed gene ontogeny and pathways related to pyrimidine metabolism, prolactin signaling, histone demethylation and immunoglobulin pathways most enriched in PS/E4H relative to PS/E3H mice (Additional file 5: Fig. S5I–J). Most of these pathway changes involved only a few genes that were enriched in the comparison analysis, ranging from 1 to 3 genes and the strength of the association (*p* values and gene numbers enriched in each pathway) were also weak. While our expectation was that the pathogenic interaction of APOE-TREM2 would be exacerbated in PS/E4H mice, surprisingly, we did not note any differential regulation (measured by fold change and *p*-value of significance) of Trem family members in the PS/E4H mice compared to PS/E3H mice (Additional file 5: Fig. S5K).

### Tau transgene induced DEG in APOE2 homozygous PS19 mice

**Fig. 6:**
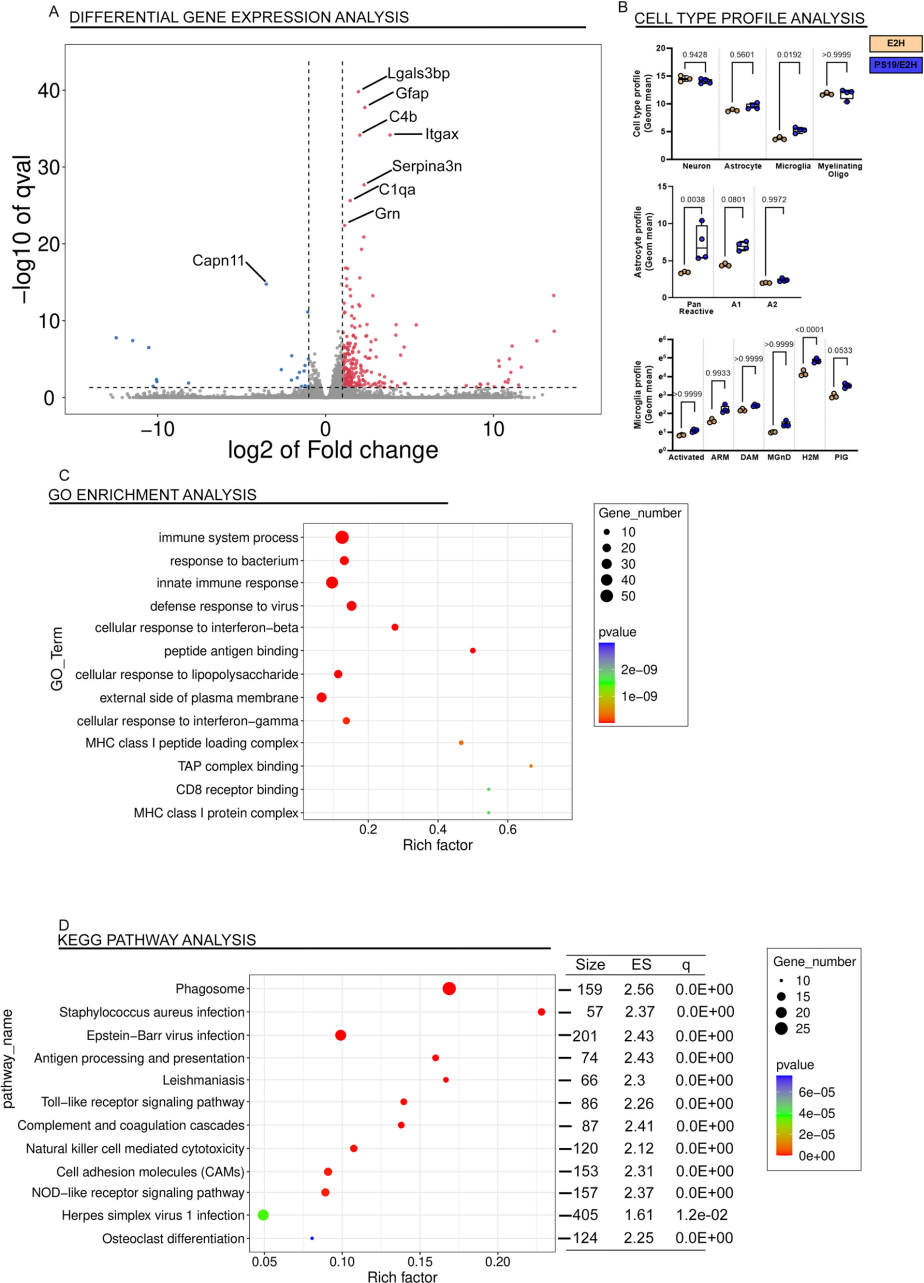
Unbiased transcriptomic analysis of PS19 mice homozygous for APOE2. The forebrains of paralyzed PS/E2H mice and 9–10-month-old E2H mice were analyzed by RNAseq. Genes (**A**), cell types (**B**), astrocyte gene expression profiles (**B**) and aging/AD-associated microglial profiles (**B**) differentially regulated in PS/E2H mice relative to E2H mice is shown. *p* values adjusted for multiple testing; FDR = 0.05. 1-way Anova. Q value denotes false discovery rate. The enriched up- and down-regulated genes were plotted onto pathways using the GO database (**C**) and KEGG pathway database (**D**) and represented in bubble plots. Pathways with an over-represented *p*-value ≤ 0.05, the number of module genes within the pathway > 15 and an enrichment score > 1.5 are depicted. The bubble plots are colored by *p*-value and sized by the enrichment score. Size, number of genes within each module; ES, Enrichment Score; q value, false discovery rate. N = 4 mice for PS/E2H and 3 mice for E2H

Relative to age-matched E2H mice, the PS/E2H showed 82 upregulated and 11 down regulated genes (Fig. 6, Additional file 6: Table S2I). Genes that were most perturbed were microglia or astrocyte-specific, such as C1qa, Grn, Itgax and Gfap (Fig. 6A), reiterating the commonalities of tau overexpression paradigm observed previously in PS/E3H and PS/E4H mice (Fig. 4 and 5). PS/E2H mice showed upregulated transcripts of Trem2 (↑2.2x, *p* = 3.7E−07), Clec7a (↑16.6x, *p* = 2.4E−15) and Treml2 (↑2.9x, *p* = 0.018), whereas APOE, Trem1 and Treml1 were unchanged. We observed only modest upregulation in microglial cell profile (*p* = 0.019), with other cell types (neurons, astrocytes and oligodendrocytes) remaining unaffected (Fig. 6B). The pan-reactive astrocyte gene expression profile (*p* = 0.0038), but not the A1/A2, was also identified (Fig. 6B). Among the aging/AD-associated transcriptional signatures, we found robust upregulation of H2M (*p* < 0.001) and modestly increased PIG (*p* = 0.0533) signature (Fig. 6B). Other AD-associated profiles, such as ARM, DAM or MGnD were not affected (Fig. 6B). Pathway analysis revealed immune system processes and defense response being enriched overall, with specific involvement of antigen processing, complement cascades, and NOD-like receptor signaling cascades in PS/E2H mice (Fig. 6C, D).

### APOE-based differences in neuropathology in human AD cases

**Fig. 7:**
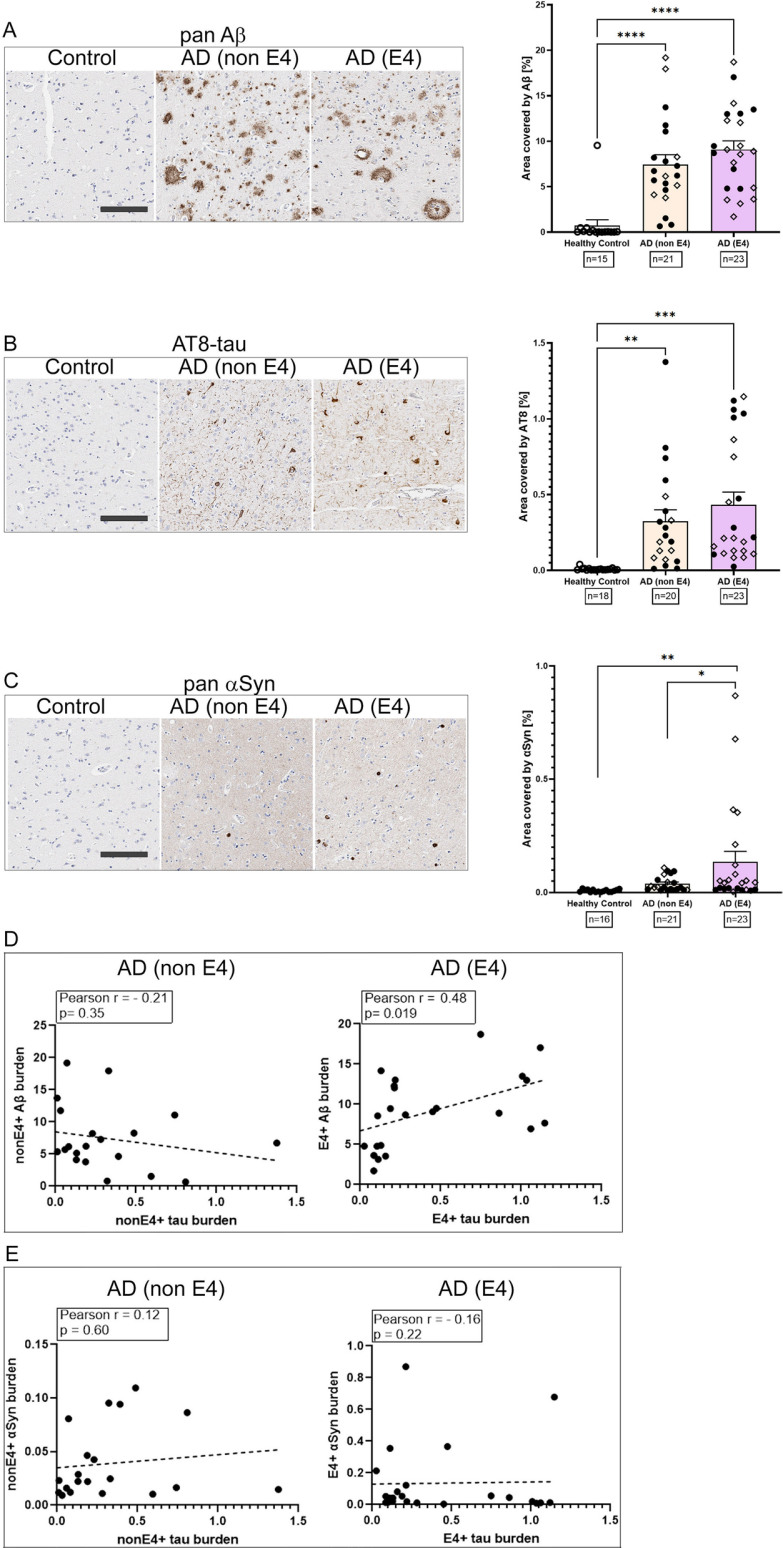
Comparative analysis of Aβ, tau and α-synuclein in AD patients. Forebrains of AD patients were stratified into E4 and non-E4 genotypes and stained for total Aβ (**A**), AT8-immunoreactive phospho-tau (**B**) and total α-synuclein (**C**). Brains of age-matched healthy controls used were all non-E4 genotype. Representative images of immunostained sections and corresponding quantitation of immunostaining shows increased Aβ, tau and α-synuclein in AD patients. Scale Bar, 150 µm; 1-way Anova with Sidak’s multiple comparison test. ***p < 0.0001; ***p < 0.001; **p < 0.01; *p < 0.05. Open circle, control non-dementia healthy cases; closed circles, pure AD cases and open diamonds, mixed AD pathology cases. (**D**) Linear covariance analysis of Aβ burden and tau burden in AD patients stratified into E4 or non-E4 genotype. (**E**) Linear covariance analysis of α-synuclein burden and tau burden in AD patients stratified into E4 or non-E4 genotype. N = 15–18 healthy controls, n = 20–21 AD (non-E4) and n = 23 (E4). Pearson’s correlation coefficient, r, and *p* value of correlation indicated on corresponding graphs

In order to understand the relationship between tau phosphorylation and APOE genotype, we next turned our attention to human AD cases that were stratified for APOE4 or non-APOE4 genotypes (Fig. 7; Additional file 6: Table S1). All of the AD cases had a primary neuropathological diagnosis of AD [17]. Some of the AD patients also exhibited co-pathology in form of α-synuclein positive Lewy bodies alongside canonical phospho-tau and amyloid β (Aβ) deposits (Additional file 6: Table S1). These patients were classified as having ‘mixed’ pathologies (open diamonds, Fig. 7). The age-matched healthy controls with no history of dementia were all non-E4 genotype (Additional file 6: Table S1). Immunostaining with pan Aβ and AT8 antibodies demonstrated that AD patients of both E4 and non-E4 genotypes showed the expected robust patterns of Aβ deposits and intracellular tau tangles relative to age-matched controls (****p < 0.0001 for Aβ; **p < 0.01 for phospho-tau) (Fig. 7A, B). We did not observe any obvious differences in the Aβ and tau burden between the E4 and non-E4 individuals within the neuropathologically-characterized AD group (Fig. 7A, B). Interestingly, we found that AD patients with E4 allele had slightly higher α-synuclein burden relative to non-E4 AD patients (*p* = 0.030) and controls (*p* = 0.0082) (Fig. 7C). We then wanted to investigate whether there was a correlation between tau and Aβ or tau and α-synuclein based on APOE4 genotype within AD patients. Phospho-tau burden was correlated to Aβ burden in the E4 AD group (*p* = 0.019) but not in the non-E4 AD group (Fig. 7D). On the other hand, tau and α-synuclein burden showed no correlation in either non-E4 or E4 AD cohorts (Fig. 7E).

## Discussion

Here, we report on life expectancy and end-stage tau pathology in tau transgenic mice with different doses of the three different human APOE alleles. In aging cohorts of PS19 mice that reached end-of-life hindlimb paralysis, APOE3 mice lived the longest, whereas both APOE4 and APOE2 homozygotes showed significantly shorter lifespan. While the APOE4 and APOE3 homozygotes had equivalent levels of phospho-tau burden (AT8, AT270, AT180 and PHF1) at end-stage, the APOE4 mice had significantly reduced lifespan compared to APOE3 homozygotes, suggesting that APOE3 conferred some resiliency to the degenerative phenotype inherent in PS19 mice. When we compared PS19 mice that are heterozygous or homozygous for human APOE4 or APOE2 (i.e., mouse carrying one human and one mouse allele), we observed that human APOE homozygous mice generally developed paralysis sooner than heterozygous counterparts. Thus, the presence of one copy of mouse Apoe modified human APOE4- and human APOE2-dependent effects on lifespan of PS19 mice. This finding suggests that either the effect of human APOE is dose-dependent, or that mouse Apoe could have modulatory effects on tau-associated neurodegeneration relative to human APOE, or that even subtle differences in mouse genetic background could have a regulatory effect on tau-APOE interactions. Overall, our data shows that presence of two alleles of human APOE3 substantially extends lifespan of PS19 mice relative to mice with heterozygous or homozygous for human APOE4, human APOE2 or mouse Apoe.

Previous studies have highlighted how APOE genotype affected tauopathy in tau transgenic animals [7], in viral-mediated tau overexpression cohorts [10] and in K18-tau seed injected tau transgenic animals [11]. Collectively, these data on APOE genotype association with tau neuropathology seem to be disparate between the studies. The first report on PS19 transgenic animals showed that APOE4 exacerbated tau burden and neurodegeneration [7] while the second study provides evidence for a highly pathologic role of APOE2 on tau pathology [10]. Additionally, in the K18-tau aggregate seeding model, induction of AT8-phospho-tau was exacerbated in APOE3 mice, but the levels of early tangle pathology did not seem to be APOE genotype-dependent [11]. In this current study, we establish the relationship of motor neuron degeneration (leading to paralysis) and tau neuropathology in PS19 transgenic mice carrying different human APOE alleles. Our data generally supports the observations of Shi and colleagues who reported that APOE4 exacerbates tau-mediated neurodegeneration in PS19 mice [7]. However, there are several differences between our current observations and this previous study. Namely, we observed equivalent phospho-tau burden in brains of PS/E4H and PS/E3H mice at end stage using several different phospho-tau antibodies, whereas the former study reported focused on AT8-tau profiles associated with APOE genotype around 9.5 months of age. A caveat to our study is that it is possible we may have missed a critical window of APOE genotype-associated differential tau pathology as we examined mice at physiological end-of-life when our cohorts are expected to reach a ‘ceiling effect’ in terms of phospho-tau burden concurrent with paralysis. Additionally, our colony of PS/E4H mice did not display consistent ventricular enlargement relative to other APOE genotypes. It is possible that our modest sample size precluded us from reaching statistically significant data on APOE genotype-related cortical atrophy due to the high degree of inter-animal variability in ventricle size within our colony. Other studies have noted inherent phenotypic variability PS19 mice [31]. Additionally, variability in phenotypes between mouse colonies maintained in different facilities have been attributed to background strain effects [32] and microbiome variabilities unique to different vivarium conditions [33]. Importantly, the accelerated motor neuron degeneration phenotype leading to paralysis in PS/E4 mice relative to PS/E3 mice mirrors the cortical degeneration phenotype reported by Shi et al. in their PS19 mice humanized for APOE4 [7].

We also observed an unexpected negative association of PS19 mice lifespan with APOE2 homozygosity relative to APOE3, in spite of modest reductions in selective soluble phospho-tau species (AT8 and AT100) in the PS/E2H mice. Notably, the immune profile determined from transcriptomic changes in PS/E2H mice were similar to PS/E3H and PS/E4H mice. It is possible that the early death phenotype in PS/E2H mice could be related to pathways independent of somatodendritic tau accumulation. In humans, APOE2 is associated with higher longevity, lower Aβ and reduced risk of AD [3]. At least in mice, the biologically beneficial effects of APOE2 on brain health has been shown to be related to cholesterol handling in the CNS [34] as well as reduced amyloid precursor protein (APP) transcription [35] relative to the reference APOE3 allele. However, during aging, APOE2 homozygous mice, by themselves, show hyperlipoproteinemia and spontaneous atherosclerosis [14], consistent with phenome-wide association studies revealing harmful effects of APOE2 homozygosity on peripheral vascular homeostasis in humans [5]. Together, our data are consistent with the idea that APOE3 confers some level of systemic resilience relative to APOE2.

AD patients are characterized by the presence of Aβ and tau pathologies, in addition to some cases showing abundant mixed pathologies such as α-synuclein and TDP-43 inclusions [36]. This does not allow for easy interpretation of APOE genotype-tau association data or establishing the individual contribution of a specific APOE genotype on Aβ, α-synuclein and tau pathogenesis separately. Using a cohort of AD patient brains that have undergone extensive post-mortem neuropathology analysis, we show that tau and Aβ levels are individually upregulated in these patients, irrespective of APOE genotype, though interestingly, α-synuclein pathology is slightly more predominant in APOE4 + individuals. Further analysis in this cohort of human AD patients appear to indicate a strong association between phospho-tau and Aβ levels, but not between phospho-tau and α-synuclein levels, in APOE4 + individuals. This implies a pathologic synergy between tau and Aβ directed by the presence of APOE4 genotype. The direct association between APOE4 and increased Aβ deposition has been corroborated over the last three decades of work in humans and mice [1, 37–39], though the direct link between APOE4 and tau tangles remain controversial [7, 40–42]. On the other hand, there is also considerable evidence that development and progression of tau tangle pathology is directly related to the extent and distribution of Aβ in AD patients and rodent models [43–47]. Thus, broadly our data is in agreement with the theory that higher levels of tau correspond to APOE4 + individuals that also have higher levels of Aβ [40, 48]. However, our data does not agree with neuropathology data derived from several cohorts that showed a direct association between APOE4 and tau neuroimaging measures in specific brain areas [49]. Further experiments are needed to experimentally establish if specific APOE genotypes are directly associated with tau and tau-associated neurodegeneration when other AD-typical pathologies such as Aβ are also present.

Our RNAseq data indicates that there are only modest overall differences between the three parental lines of APOE homozygous mice, especially between APOE4 and APOE3 mice where the major differences centered on fatty acid metabolism and cytosolic DNA sensing pathway (inflammasome and Type 1 IFN response). Additionally, APOE4 and APOE2 comparisons showed differential regulation of immune signaling pathways relative to APOE3. Notably, Serpina3n is upregulated in APOE4 mice relative to both APOE3 and APOE2 mice, which is a key mediator of reactive astrocytosis [50]. Erdr1, a stress-induced cell survival factor, was downregulated in APOE4 mice relative to APOE3 and APOE2 mice while Eps8l1, a tauopathy-related Bin1-responsive gene, was downregulated in APOE3 mice relative to APOE4 and APOE2 mice [51, 52]. Interestingly, the presence of tau transgene led to the emergence of overwhelmingly robust immune transcriptome, irrespective of the APOE genotype. Indeed, when we analyzed the differential transcriptome in PS19 mice carrying APOE4 or APOE2 or APOE3 relative to their corresponding parental APOE lines, we found that immune response and stress response pathways were commonly upregulated in PS19 mice irrespective of the APOE genotype. We also found that tau overexpression and accompanying tau neuropathology increased transcription of Trem family members in these PS19 mice, consistent with previous evidence of a pathologic relationship between Trem2 and Apoe [29], though there was no clear APOE genotype-dependent outcome noticed in our study. Originally these Apoe/Trem2-associated DAM microglial phenotypes were characterized in aging mouse brains associated with Aβ- or demyelinated-associated neurodegeneration [53]. Subsequent work showed that loss of Trem2 worsened tau pathology, microgliosis and neurodegeneration in APOE4 mice [54] but not in mice carrying mouse Apoe [55]. Together, these previous studies suggested that there could be a pathologic relationship between APOE4, tau and Trem2-microglia function, though there is a lack of consensus on underlying mechanisms. In our Rnaseq analyses on paralyzed cohorts, we did not observe a clear differential upregulation of the Trem2-associated DAM microglial profile in any particular APOE genotypes. On the other hand, our transcriptome studies showed that the homeostatic H2M signature was upregulated in all cohorts of PS19 mice, independent of APOE genotype. It is possible that the microglia in our PS19xAPOE cohorts are persisting in the homeostatic phase, pausing prior to maturing into DAM or other degenerative phenotype in the absence of additional insults. This could possibly suggest that the PS/E4H mice could require additional triggers, such as Tgfβ or other AD-related factors, for gradual differentiation into progressively dystrophic Apoe/Trem2-associated DAM state [53]. We also noted that the APOE4 mice uniquely showed modest upregulation of another AD-associated profile, the PIG, that was identified from APP/Aβ model and human AD brains and is related to neurodegenerative stimulus [25]. Future studies could focus on relating the relative abundance of these transcriptional signatures to APOE genotype-dependent biological outcomes.

In conclusion, our studies demonstrate that relative to human APOE2 and APOE4, the APOE3 allele delays paralysis in PS19 mice. At end-stage, the burden of phospho-tau pathology was similar among the three APOE genotypes, with some select phospho-tau epitopes being under-represented in APOE2 mice. Robust immune changes in the transcriptome was noted in PS19 mice, irrespective of the APOE genotype. Our findings indicate that APOE genotype plays a regulatory role in tauopathy, though the exact pathogenic mechanism remains elusive.

## Supplementary Information

**Additional file 1: Fig. S1**. Analysis of aging cohorts of PS19xAPOE mice. Representative images of hematoxylin & eosin-stained brains and volumes of ventricles at two locations (bregma +2.6mm denoted as ‘lateral’, A, and bregma +3.2mm denoted as ‘medial’, B) in PS19 mice homozygous for APOE2, APOE3 or APOE4 shown. Male mice indicated in blue; female mice indicated in pink. N = 6 (PS/E2H), n = 6 (PS/E3H), n = 8 (PS/E4H).

**Additional file 2: Fig. S2**. Analysis of phospho-tau epitopes in paralyzed PS19 mice homozygous for APOE2, APOE3 or APOE4. A-B. Representative images from hippocampus and corresponding quantitation of whole brain burden of phospho-tau in PS19 mice homozygous for APOE2 (triangle), APOE3 (circle) or APOE4 (diamond). 3 different phospho-tau epitopes were tested: AT100, AT270 and AT180. 1-way Anova. N = 10 (PS/E2H), n = 8 (PS/E3H), n = 9 (PS/E4H). C-G. Biochemical analysis of phospho-tau using various antibodies in detergent soluble (S1) and detergent-insoluble (S3 pellet) fractionated brains of paralyzed mice. Band intensities of phospho-tau in S1 fraction have been normalized to GAPDH, while band intensities of phospho-tau in S3 fraction (if detectable) have been normalized to total tau. Numerals on the left side of each blot denote molecular weight standards in kDa. As no phospho-tau was detected for AT100 (C), AT180 (E) and AT8 (G), S3-phospho-tau values have not been plotted. S1- and S3- phospho-tau levels have been presented for AT270 (D) and PHF1 (F) epitopes. Total tau levels in S3 fraction corresponding to the AT8-stained blot (G) is shown in Fig. 1D. The loading order has been denoted by consecutive numbering and the key is presented (H). 1-way Anova; *p<0.05. n = 3 mice/genotype.

**Additional file 3: Fig. S3**. Inflammation markers in paralyzed PS19 mice homozygous for APOE2, APOE3 or APOE4. Immunoblots representing Iba-1 (A) and GFAP (B) levels from the whole forebrain and corresponding quantitation of protein bands are presented for PS19 mice carrying mouse ApoE, APOE mice (with no tau) or PS19 mice carrying human APOE alleles. Individual protein bands were normalized to actin and data from 3 different blots were averaged for the graph. N = 3 mice/group. 1-way Anova, **p<0.01, *p<0.05.

**Additional file 4: Fig. S4**. Lifespan analysis and neuropathological characterization of PS19 mice heterozygous for APOE. PS/E2h, PS/E3h and PS/E4h mice were aged to paralysis as indicated (A). Median age to paralysis of these APOE heterozygous ‘h’ mice are compared to APOE-genotype matched homozygous ‘H’ mice - PS/E2H, PS/E3H and PS/E4H mice (shown in Fig. 1) (B). Log-rank (Mantel-Cox) test. Significant p values are in bold. n = 34 (PS/E2h), n = 31 (PS/E3h), n = 43 (PS/E4h). Paralyzed PS/E2h, PS/E3h, PS/E4h mice were analyzed for phospho-tau, microgliosis and astrocytosis levels using AT8 (C), Iba-1 (D) and GFAP (E) antibodies respectively. Quantification of AT8, Iba-1, and GFAP immunostaining is presented as % immunoreactivity in the cortex and hippocampus underneath corresponding antibody-stained panels. n = 12 (PS/E2h), n = 11 (PS/E3h), n = 26 (PS/E4h). 1-way ANOVA, *p<0.05. Scale bar: 100 µm.

**Additional file 5: Fig. S5**. Comparative RNAseq analysis of PS/E2H, PS/E3H and PSE4H mice. Heat map (A) and individual volcano plots (B-D) of differentially expressed genes in PS/E2H, PS/E3H and PSE4H mice. Q value (qval) indicates false discovery rate. N = 4 mice/group. Asterisks in volcano plot refer to predicted genes. Commonly enriched gene sets (upregulated or downregulated) were identified from a comparison of PS/E2H vs PS/E3H and E2H vs E3H to enumerate genes that are driven by the variation in APOE allele. Total number of significantly altered genes are indicated by numerals within the Venn diagram. The comonly altered genes (in the overlapping Venn diagram) were used to map onto KEGG pathways for this comparison (E). Similar KEGG pathway mapping was done for PS/E3H vs E3H and PS/E2H vs E2H (F), PS/E4H vs PS/E3H and E4H vs E3H (G), PS/E4H vs E4H and PS/E3H vs E3H (H) using overlapping gene sets from the Venn diagram. Enriched gene sets from comparison of PS/E4H vs PS/E3H is represented as KEGG pathways (I) and GO pathways (J). The bubble plots (I, J) are colored by p-value and sized by number of genes in enriched gene sets. Tabulation of gene expression levels of Trem family genes (K) from PS/E4H vs PS/E3H analysis. BaseMean, means of normalized counts of all samples; FC, Fold change; lFcSE, log of Fold change Standard Error; stat, Wald statistics. N = 3-4 mice/group.

**Additional file 6: Table S1.** Detailed information of human brains used in the study. Individually characterized brains were obtained from the University of Florida Neuromedicine Human Brain and Tissue Bank. Experimental Group denotes the designation used in this study (Fig. 7). Dx1, primary diagnosis based on neuropathology; Dx2, Dx3, Dx4, secondary diagnoses based on neuropathology; Thal phase, burden of immunostained amyloid deposits in cortical and subcortical area; CERAD score, neuritic plaque frequency; AD, Alzheimer's disease; ARTAG, Aging-related tau astrogliopathy; CAA, cerebral amyloid angiopathy; CVD, cardiovascular disease; LATE-NC, limbic-predominant age-related TDP-43 encephalopathy neuropathological change; LBD, Lewy body dementia; PART, primary age-related tauopathy. **Table S2.** Transcriptome analysis of forebrains of PS19 mice homozygous for APOE2, APOE3 or APOE4 and corresponding APOE TR lines. RNAseq data has been deposited in GEO database. A selection of genes that are altered in each experimental group comparisons are presented. The corresponding tables show the DEG analysis from the following comparisons: Table S2A, E2H vs E3H; Table S2B, E4H vs E3H; Table S2C, E4H vs E2H; Table S2D, PS/E2H vs PS/E3H; Table S2E, PS/E4H vs PS/E3H; Table S2F, PS/E4H vs PS/E2H; Table S2G, PS/E4H vs E4H; Table S2H, PS/E3H vs E3H; Table S2I, PS/E2H vs E2H.

## Data Availability

All data and code are available from the corresponding author upon reasonable request following publication. The RNAseq data is available from GEO database (GSE226093).

## Abbreviations

Aβ: Amyloid β
AD: Alzheimer’s disease
APOE: Apolipoprotein E
APP: Amyloid precursor protein
CBD: Cortico-basal degeneration
DAM: Damage associated microglia
DEG: Differential expression of genes
E2H: APOE2 targeted replacement mouse
E3H: APOE3 targeted replacement mouse
E4H: APOE4 targeted replacement mouse
FBS: Fetal bovine serum
FFPE: Formalin fixed, paraffin embedded
FPKM: Fragments per kilobase of exon per million mapped fragments
GFAP: Glial fibrillary acidic protein
H2M: Homeostatic microglia
MGnD: Neurodegeneration associated microglia
NES: Normalized enrichment score
NOM *p-* value: Nominal *p* value
Phospho-tau: Phosphorylated tau
PIG: Plaque induced genes
PS/E2H: PS19 mice Homozygous for APOE2
PS/E3H: PS19 mice Homozygous for APOE3
PS/E4H: PS19 mice Homozygous for APOE4
PS/E2h: PS19 mice heterozygous for APOE2
PS/E3h: PS19 mice heterozygous for APOE3
PS/E4h: PS19 mice heterozygous for APOE4
PSP: Progressive supranuclear palsy
RFU: Relative fluorescent units
SNP: Single nucleotide polymorphism
