# Supplementary figures and images for: Humanized APOE genotypes influence lifespan independently of tau aggregation in the P301S mouse model of tauopathy
Source: Acta Neuropathol Commun. 2023 Jun 19;11:99. doi: 10.1186/s40478-023-01581-2 (PMC10280946; doi:10.1186/s40478-023-01581-2)

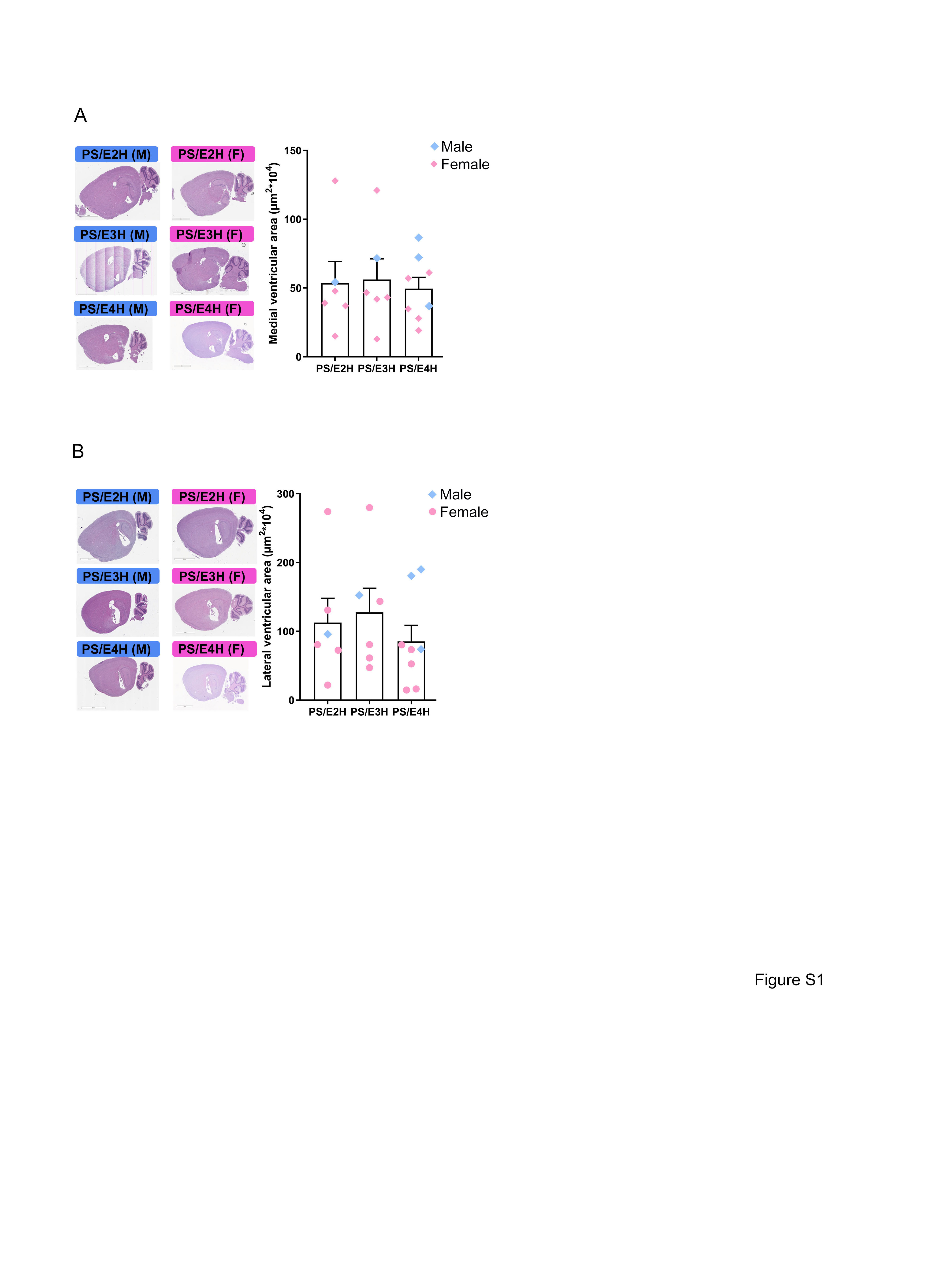

Supplement: Supplementary file 1 — Additional file 1: Fig. S1. Analysis of aging cohorts of PS19xAPOE mice. Representative images of hematoxylin & eosin-stained brains and volumes of ventricles at two locations (bregma +2.6mm denoted as ‘lateral’, A, and bregma +3.2mm denoted as ‘medial’, B) in PS19 mice homozygous for APOE2, APOE3 or APOE4 shown. Male mice indicated in blue; female mice indicated in pink. N = 6 (PS/E2H), n = 6 (PS/E3H), n = 8 (PS/E4H). [file 40478_2023_1581_MOESM1_ESM.jpg]

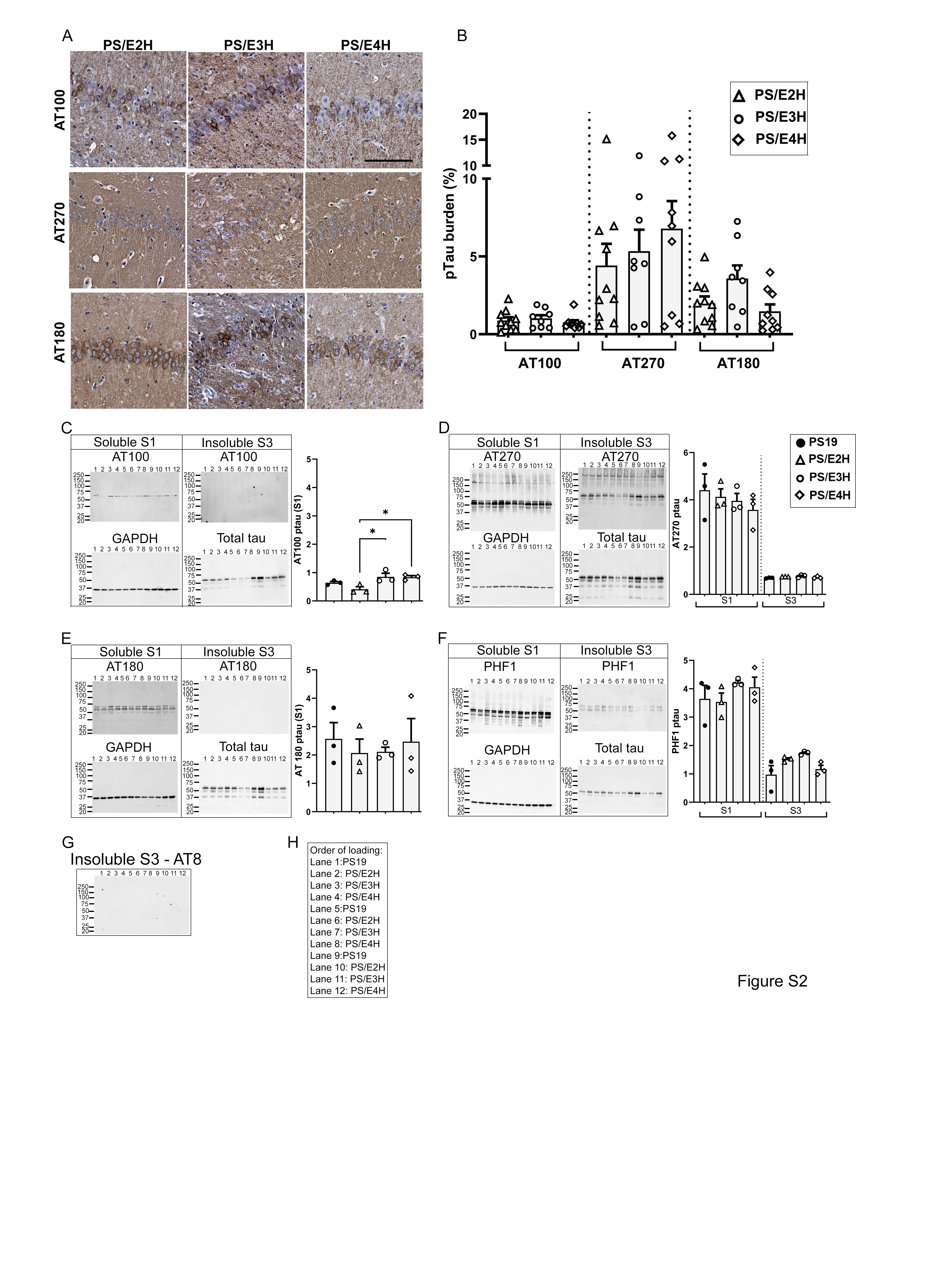

Supplement: Supplementary file 2 — Additional file 2: Fig. S2. Analysis of phospho-tau epitopes in paralyzed PS19 mice homozygous for APOE2, APOE3 or APOE4. A-B. Representative images from hippocampus and corresponding quantitation of whole brain burden of phospho-tau in PS19 mice homozygous for APOE2 (triangle), APOE3 (circle) or APOE4 (diamond). 3 different phospho-tau epitopes were tested: AT100, AT270 and AT180. 1-way Anova. N = 10 (PS/E2H), n = 8 (PS/E3H), n = 9 (PS/E4H). C-G. Biochemical analysis of phospho-tau using various antibodies in detergent soluble (S1) and detergent-insoluble (S3 pellet) fractionated brains of paralyzed mice. Band intensities of phospho-tau in S1 fraction have been normalized to GAPDH, while band intensities of phospho-tau in S3 fraction (if detectable) have been normalized to total tau. Numerals on the left side of each blot denote molecular weight standards in kDa. As no phospho-tau was detected for AT100 (C), AT180 (E) and AT8 (G), S3-phospho-tau values have not been plotted. S1- and S3- phospho-tau levels have been presented for AT270 (D) and PHF1 (F) epitopes. Total tau levels in S3 fraction corresponding to the AT8-stained blot (G) is shown in Fig. 1D. The loading order has been denoted by consecutive numbering and the key is presented (H). 1-way Anova; *p<0.05. n = 3 mice/genotype. [file 40478_2023_1581_MOESM2_ESM.jpg]

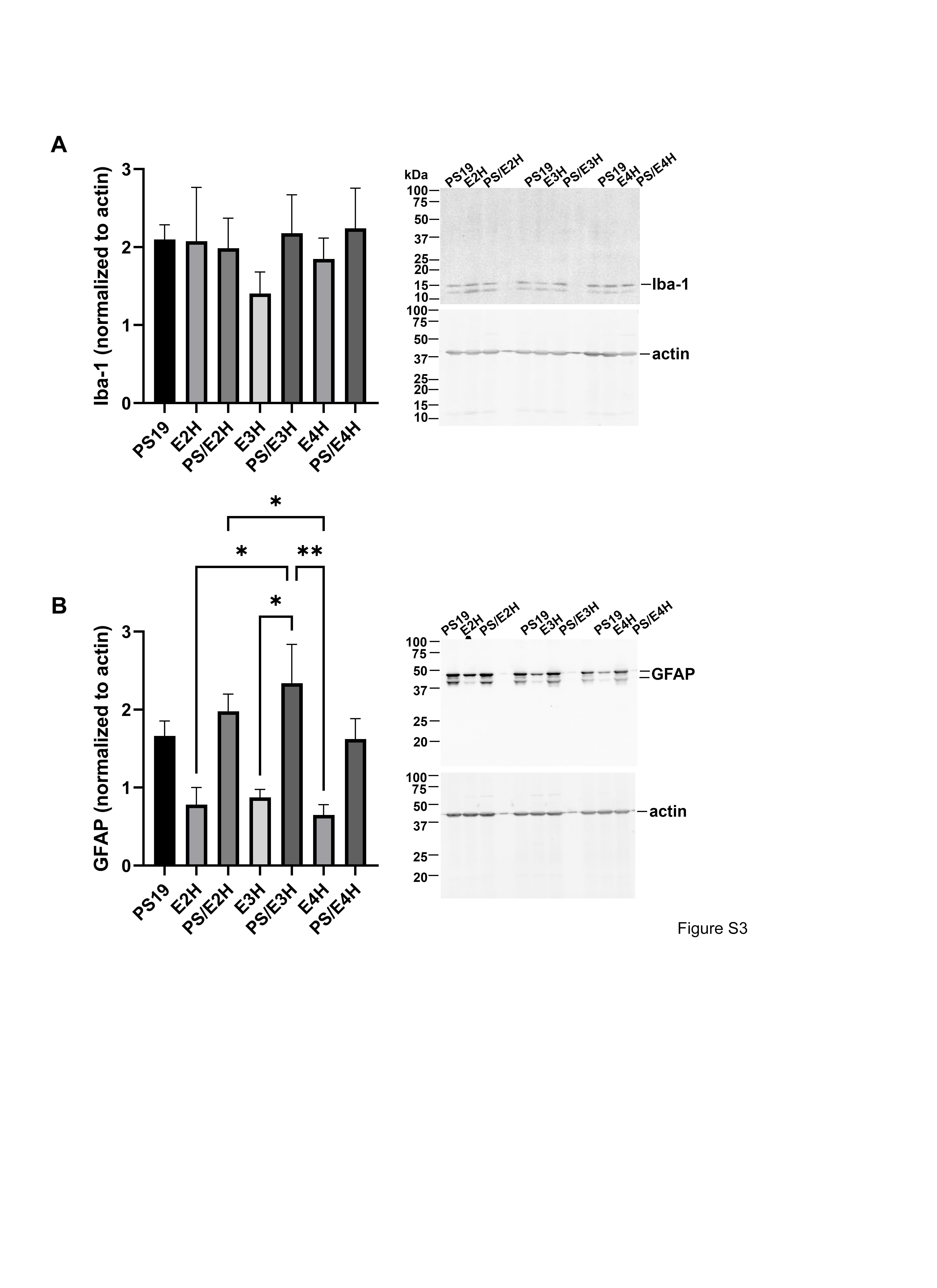

Supplement: Supplementary file 3 — Additional file 3: Fig. S3. Inflammation markers in paralyzed PS19 mice homozygous for APOE2, APOE3 or APOE4. Immunoblots representing Iba-1 (A) and GFAP (B) levels from the whole forebrain and corresponding quantitation of protein bands are presented for PS19 mice carrying mouse ApoE, APOE mice (with no tau) or PS19 mice carrying human APOE alleles. Individual protein bands were normalized to actin and data from 3 different blots were averaged for the graph. N = 3 mice/group. 1-way Anova, **p<0.01, *p<0.05. [file 40478_2023_1581_MOESM3_ESM.jpg]

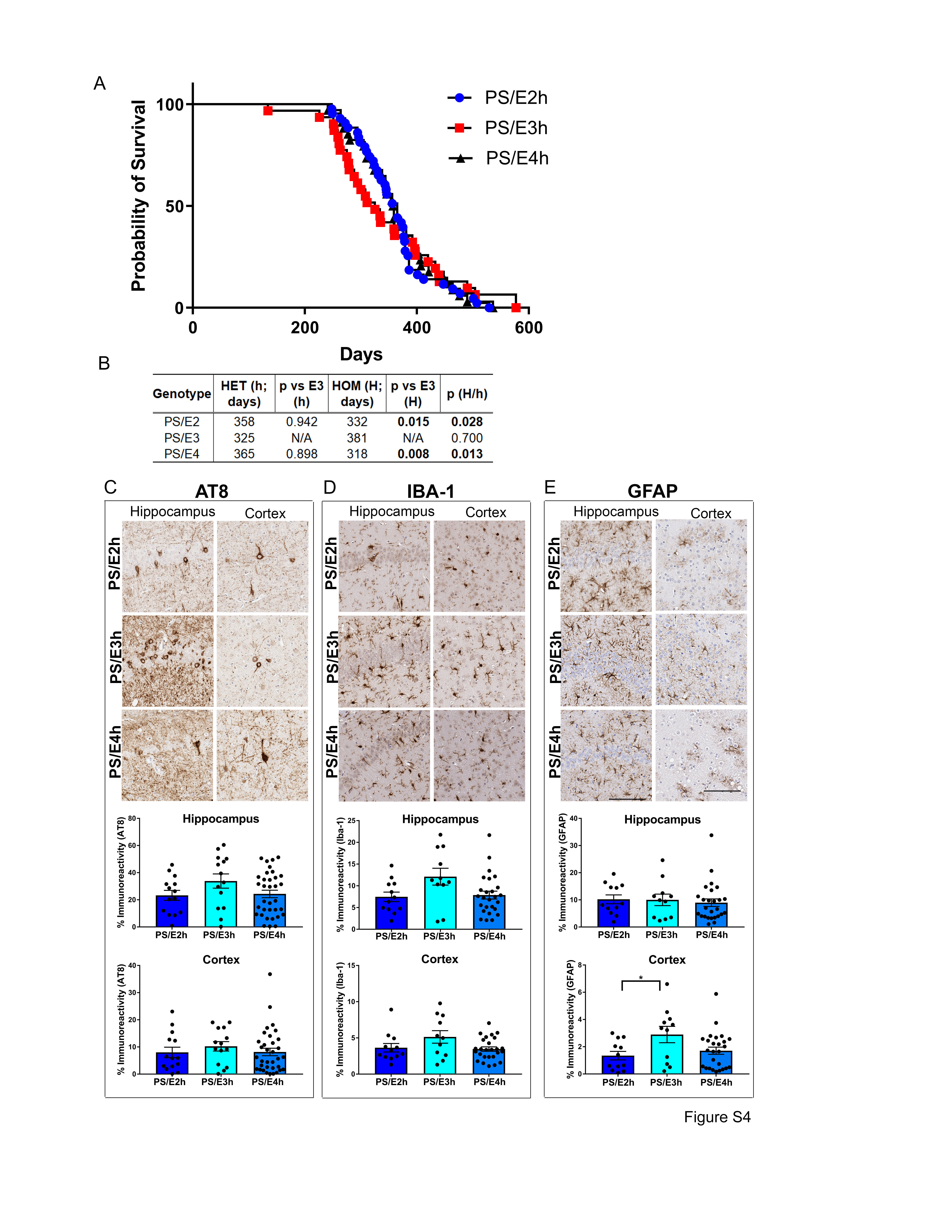

Supplement: Supplementary file 4 — Additional file 4: Fig. S4. Lifespan analysis and neuropathological characterization of PS19 mice heterozygous for APOE. PS/E2h, PS/E3h and PS/E4h mice were aged to paralysis as indicated (A). Median age to paralysis of these APOE heterozygous ‘h’ mice are compared to APOE-genotype matched homozygous ‘H’ mice - PS/E2H, PS/E3H and PS/E4H mice (shown in Fig. 1) (B). Log-rank (Mantel-Cox) test. Significant p values are in bold. n = 34 (PS/E2h), n = 31 (PS/E3h), n = 43 (PS/E4h). Paralyzed PS/E2h, PS/E3h, PS/E4h mice were analyzed for phospho-tau, microgliosis and astrocytosis levels using AT8 (C), Iba-1 (D) and GFAP (E) antibodies respectively. Quantification of AT8, Iba-1, and GFAP immunostaining is presented as % immunoreactivity in the cortex and hippocampus underneath corresponding antibody-stained panels. n = 12 (PS/E2h), n = 11 (PS/E3h), n = 26 (PS/E4h). 1-way ANOVA, *p<0.05. Scale bar: 100 µm. [file 40478_2023_1581_MOESM4_ESM.jpg]

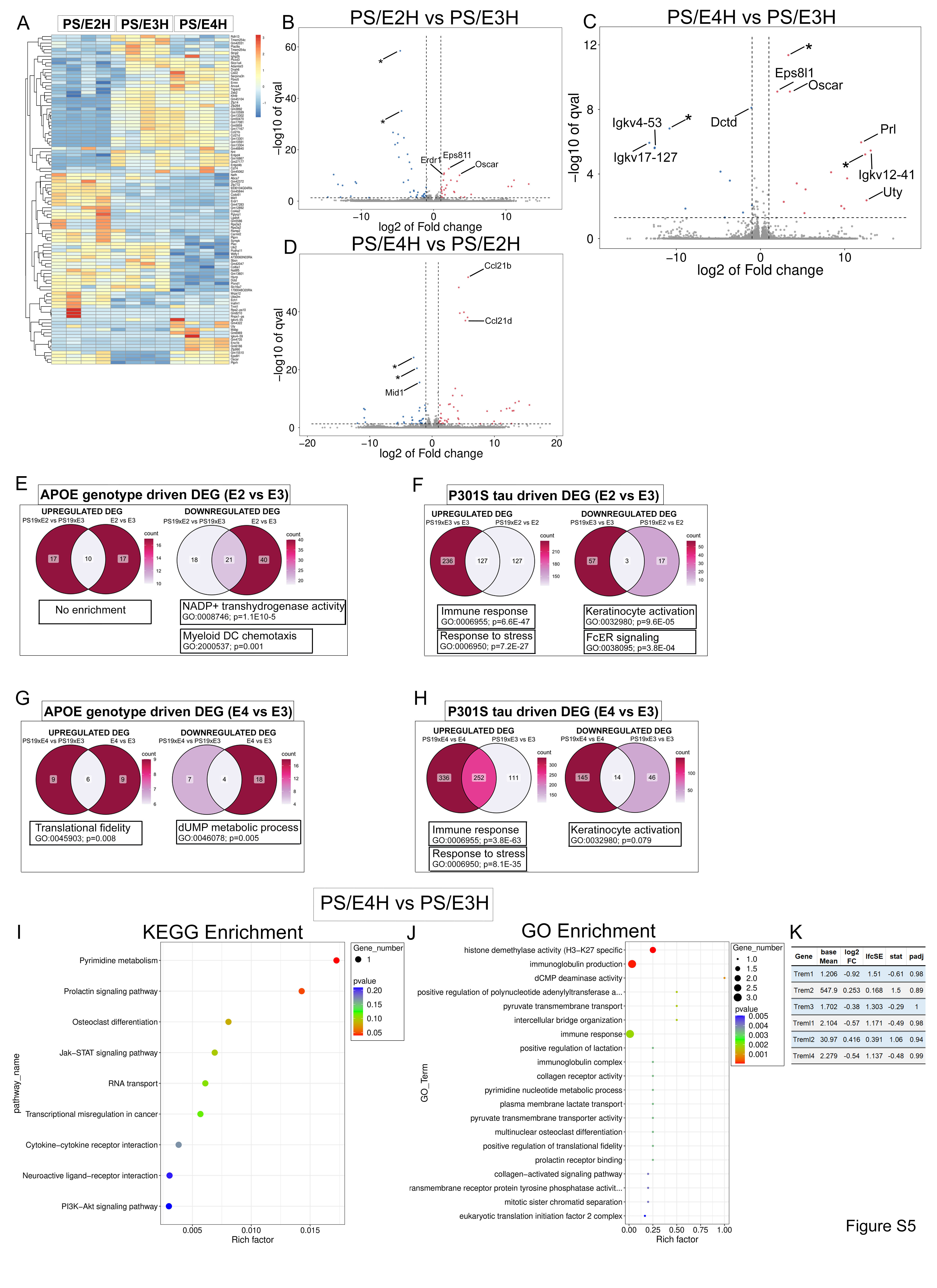

Supplement: Supplementary file 5 — Additional file 5: Fig. S5. Comparative RNAseq analysis of PS/E2H, PS/E3H and PSE4H mice. Heat map (A) and individual volcano plots (B-D) of differentially expressed genes in PS/E2H, PS/E3H and PSE4H mice. Q value (qval) indicates false discovery rate. N = 4 mice/group. Asterisks in volcano plot refer to predicted genes. Commonly enriched gene sets (upregulated or downregulated) were identified from a comparison of PS/E2H vs PS/E3H and E2H vs E3H to enumerate genes that are driven by the variation in APOE allele. Total number of significantly altered genes are indicated by numerals within the Venn diagram. The comonly altered genes (in the overlapping Venn diagram) were used to map onto KEGG pathways for this comparison (E). Similar KEGG pathway mapping was done for PS/E3H vs E3H and PS/E2H vs E2H (F), PS/E4H vs PS/E3H and E4H vs E3H (G), PS/E4H vs E4H and PS/E3H vs E3H (H) using overlapping gene sets from the Venn diagram. Enriched gene sets from comparison of PS/E4H vs PS/E3H is represented as KEGG pathways (I) and GO pathways (J). The bubble plots (I, J) are colored by p-value and sized by number of genes in enriched gene sets. Tabulation of gene expression levels of Trem family genes (K) from PS/E4H vs PS/E3H analysis. BaseMean, means of normalized counts of all samples; FC, Fold change; lFcSE, log of Fold change Standard Error; stat, Wald statistics. N = 3-4 mice/group. [file 40478_2023_1581_MOESM5_ESM.jpg]
