# Supplementary material for: Humanized APOE genotypes influence lifespan independently of tau aggregation in the P301S mouse model of tauopathy
Source: Acta Neuropathol Commun. 2023 Jun 19;11:99. doi: 10.1186/s40478-023-01581-2 (PMC10280946; doi:10.1186/s40478-023-01581-2)
Supplement: Supplementary file 6 — Additional file 6: Table S1. Detailed information of human brains used in the study. Individually characterized brains were obtained from the University of Florida Neuromedicine Human Brain and Tissue Bank. Experimental Group denotes the designation used in this study (Fig. 7). Dx1, primary diagnosis based on neuropathology; Dx2, Dx3, Dx4, secondary diagnoses based on neuropathology; Thal phase, burden of immunostained amyloid deposits in cortical and subcortical area; CERAD score, neuritic plaque frequency; AD, Alzheimer's disease; ARTAG, Aging-related tau astrogliopathy; CAA, cerebral amyloid angiopathy; CVD, cardiovascular disease; LATE-NC, limbic-predominant age-related TDP-43 encephalopathy neuropathological change; LBD, Lewy body dementia; PART, primary age-related tauopathy. Table S2. Transcriptome analysis of forebrains of PS19 mice homozygous for APOE2, APOE3 or APOE4 and corresponding APOE TR lines. RNAseq data has been deposited in GEO database. A selection of genes that are altered in each experimental group comparisons are presented. The corresponding tables show the DEG analysis from the following comparisons: Table S2A, E2H vs E3H; Table S2B, E4H vs E3H; Table S2C, E4H vs E2H; Table S2D, PS/E2H vs PS/E3H; Table S2E, PS/E4H vs PS/E3H; Table S2F, PS/E4H vs PS/E2H; Table S2G, PS/E4H vs E4H; Table S2H, PS/E3H vs E3H; Table S2I, PS/E2H vs E2H. [file 40478_2023_1581_MOESM6_ESM.pdf]

Table S1. Detailed information of human brains used in the study. Individually characterized brains were obtained from the the University of Florida Neuromedicine Human Brain and Tissue Bank. Experimental Group denotes the designation for this study (Fig. 7). Dx1, primary diagnosis based on neuropathology; Dx2, Dx3, Dx4, secondary diagnoses based on neuropathology; Thal phase, burden of immunostained amyloid deposits in cortical and subcortical area; CERAD score, neuritic plaque frequency; AD, Alzheimer's disease; ARTAG, Aging-related tau astrogliopathy; CAA, cerebral amyloid angiopathy; CVD, cardiovascular disease; LATE-NC, limbic-predominant age-related TDP-43 encephalopathy neuropathological change; LBD, Lewy body dementia; PART, primary age-related tauopathy.

Experimental Group	Sex	APOE	Age	Dx1	Dx2	Dx3	Thal Stage	Braak Stage	CERAD Score
Control	m	3/3	90	AD low			1	II	none
Control	m	3/3	88	AD low	CVD		1	II	none
Control	f	3/3	72	PART, definite, Braak II			0	II	none
Control	m	3/3	71	PART, Braak I	CAA widespread, mild		0	I	none
Control	m	3/3	84	Atherosclerosis (moderate)	AD low		1	I	none
Control	f	3/3	76	PART, Braak I	Atherosclerosis (moderate)		0	I	none
Control	m	3/3	85	PART, definite, Braak II			0	II	none
Control	m	3/3	71	Atherosclerosis (moderate)			0	0	none
Control	m	3/3	88	Atherosclerosis (moderate)			0	0	none
Control	m	3/3	87	PART, definite, Braak I	LATE stage 1		0	I	none
Control	m	3/3	77	PART, definite, Braak I	ARTAG, subependymal		0	I	none
Control	m	3/3	72	Acute microinfarcts			0	0	none
Control	f	2/3	73	PART, definite, Braak I			0	I	none
Control	f	3/3	90	PART, definite, Braak II	subacute microinfarct corpus callosum		0	II	none
Control	f	3/3	78	PART, definite, Braak I			0	I	none
Control	m	3/3	77	PART, definite, Braak II	CAA focal, moderate		0	II	none
Control	m	2/3	51	PART, definite, Braak I	ARTAG, subependymal		0	I	none
Control	m	2/3	52	PART, definite, Braak I			0	I	none
AD	f	3/4	72	AD high	CAA widespread, severe		5	V	frequent
AD	m	3/4	84	AD high	CAA (moderate)		4	V	frequent
AD	m	3/3	83	AD high	CAA widespread, moderate		5	VI	frequent
AD	f	3/3	78	AD high	CAA widespread, moderate		5	V	frequent
AD	f	3/3	78	AD high	CAA widespread, mild to moderate	LATE-NC stage 2	4	V	frequent
AD	m	3/4	78	AD high	CAA focal, mild		5	V	frequent
AD	m	3/4	81	AD high	CAA (moderate)	LATE-NC stage 1	5	VI	frequent
AD	m	3/4	63	AD high	CAA widespread, moderate		5	VI	frequent
AD	m	3/3	66	AD high	CAA widespread, mild to moderate		5	VI	frequent
AD	m	3/4	77	AD high	CAA widespread, mild		4	V	frequent
AD	m	3/3	95	AD high	CAA focal, moderate		5	V	frequent
AD	m	3/3	83	AD high	CAA widespread, moderate		5	VI	frequent
AD	m	3/4	59	AD high	CAA widespread, moderate		5	VI	frequent
AD	m	3/3	60	AD high	CAA widespread, moderate to severe		5	VI	frequent
AD	m	4/4	70	AD high	CAA widespread, moderate		5	VI	frequent
AD	m	2/3	74	AD high	CAA widespread, moderate to severe		5	V	frequent
AD	m	4/4	75	AD high	CAA widespread, moderate		4	VI	frequent
AD	f	3/3	86	AD high	CAA focal, moderate		5	VI	frequent
AD	f	3/3	84	AD high	CAA focal, moderate	LATE-NC stage 1	5	VI	frequent
AD	f	3/3	90	AD high	CAA focal, mild	LATE-NC stage 1	5	V	frequent
AD	m	3/3	87	AD high	LATE stage 2		5	V	frequent
AD	m	3/3	73	AD high	CAA focal, mild to moderate		5	V	frequent
Mixed (AD/LBD)	f	2/4	74	AD high	CAA widespread, moderate	LBD diffuse neocortical	5	V	frequent
Mixed (AD/LBD)	m	3/4	77	AD high	CAA (moderate)	LBD (neocortical)	4	V	frequent
Mixed (AD/LBD)	f	3/4	83	AD high	LBD diffuse neocortical	CAA widespread, moderate	5	V	frequent
Mixed (AD/LBD)	m	3/3	87	AD high	LBD (neocortical)	CAA (moderate to severe)	5	VI	frequent
Mixed (AD/LBD)	m	3/4	80	AD high	LBD diffuse neocortical	CAA widespread, moderate	5	V	frequent
Mixed (AD/LBD)	f	3/4	68	LBD diffuse neocortical	AD high	CAA	5	V	frequent
Mixed (AD/LBD)	m	3/4	78	AD high	LBD diffuse neocortical	CAA focal, mild	5	VI	frequent
Mixed (AD/LBD)	m	4/4	91	AD high	CAA widespread, moderate to severe	LBD diffuse neocortical	5	VI	frequent
Mixed (AD/LBD)	f	3/4	76	AD high	LBD diffuse neocortical	LATE-NC stage 3	4	V	frequent
Mixed (AD/LBD)	m	3/3	83	AD high	CAA widespread, moderate	CAA widespread, moderate	5	VI	frequent
Mixed (AD/LBD)	m	3/3	62	AD high	LBD diffuse neocortical	CAA focal, moderate	5	VI	frequent
Mixed (AD/LBD)	f	4/4	76	AD high	CAA widespread, mild	LBD limbic-transitional	5	VI	frequent
Mixed (AD/LBD)	f	3/3	81	AD high	CAA widespread, moderate	LBD limbic-transitional	5	VI	frequent
Mixed (AD/LBD)	m	3/4	64	AD high	CAA widespread, moderate	LATE stage 2	5	VI	frequent
Mixed (AD/LBD)	f	3/3	78	AD high	CAA, widespread, moderate	LBD limbic-transitional	5	V	frequent
Mixed (AD/LBD)	f	3/3	63	AD high	LBD diffuse neocortical	LATE-NC stage 3	5	V	frequent
Mixed (AD/LBD)	f	2/3	98	AD high	LBD diffuse neocortical	LATE-NC stage 3	5	V	frequent
Mixed (AD/LBD)	f	4/4	93	AD high	CAA, widespread, moderate	LBD diffuse neocortical	5	V	frequent
Mixed (AD/LBD)	f	4/4	75	AD high	LBD diffuse neocortical	LATE stage 2	5	VI	frequent
Mixed (AD/LBD)	f	4/4	85	AD high	LBD limibic-transitional	CAA focal, moderate	5	V	frequent
Mixed (AD/LBD)	m	3/4	74	AD high	LBD diffuse neocortical		5	V	frequent

Table S2A: Selection of genes altered in E2H vs E3H mice. FPKM values of genes in individual mice (indicated in underscored numeral), along with fold change (fc) value, p value adjusted for false discovery rate of 0.05 (q value, qval) are presented. N=3 mice/group.

gene_id	gene_name	Description	FPKM.E2H_1	FPKM.E2H_2	FPKM.E2H_3	FPKM.E3H_1	FPKM.E3H_2	FPKM.E3H_3	fc	log2 (fc)	pval	qval	Log10 (qval)	regulation	significant
ENSMUSG00000093954	Gm16867	predicted gene	0.07	0.04	0.07	1.92	2.21	2.44	0.03	-5.26	4.76E-118	1.57E-113	-112.80	down	yes
ENSMUSG00000098975	Gm27177	predicted gene	0.06	0.12	0.07	1.35	2.02	1.59	0.05	-4.29	1.81E-58	2.99E-54	-53.52	down	yes
ENSMUSG00000118458	Gm10599	predicted pseu	0.12	0.10	0.09	2.66	2.52	2.13	0.04	-4.55	5.34E-46	5.88E-42	-41.23	down	yes
ENSMUSG00000109209	Gm45104	predicted gene	0.17	0.26	0.24	1.92	2.08	2.46	0.10	-3.28	3.63E-42	3.00E-38	-37.52	down	yes
ENSMUSG00000090338	Gm17081	predicted gene	0.07	0.07	0.06	2.40	2.39	1.58	0.03	-5.01	2.26E-40	1.49E-36	-35.83	down	yes
ENSMUSG00000096596	Gm10591	predicted gene	0.10	0.08	0.03	6.25	9.89	4.52	0.01	-6.63	3.69E-40	2.03E-36	-35.69	down	yes
ENSMUSG00000091542	Gm17167	predicted gene	0.08	0.05	0.05	1.28	1.18	0.82	0.06	-4.18	1.33E-36	6.25E-33	-32.20	down	yes
ENSMUSG00000073878	Gm13304	predicted gene	0.15	0.14	0.03	6.65	5.72	3.84	0.02	-5.66	3.59E-34	1.48E-30	-29.83	down	yes
ENSMUSG00000095675	Ccl21b	chemokine (C-C	0.14	0.10	0.01	7.91	3.65	5.15	0.02	-6.01	2.42E-33	8.88E-30	-29.05	down	yes
ENSMUSG00000073879	Gm5859	predicted pseu	0.12	0.07	0.09	1.83	1.73	1.38	0.06	-4.15	1.08E-29	3.55E-26	-25.45	down	yes
ENSMUSG00000006154	Eps8l1	EPS8-like 1 [S	0.96	0.88	0.78	0.14	0.12	0.14	6.64	2.73	1.67E-28	5.02E-25	-24.30	up	yes
ENSMUSG00000094065	Ccl21d	chemokine (C-C	0.11	0.10	0.02	2.62	3.44	3.30	0.03	-5.31	3.27E-24	8.99E-21	-20.05	down	yes
ENSMUSG00000097842	9330104G04Ri	RIKEN cDNA 933	1.37	1.56	1.63	0.33	0.24	0.23	5.74	2.52	2.38E-23	6.04E-20	-19.22	up	yes
ENSMUSG00000022066	Entpd4b	ectonucleoside	5.18	4.64	5.10	9.77	9.87	10.64	0.49	-1.02	1.11E-22	2.62E-19	-18.58	down	yes
ENSMUSG00000025453	Nnt	nicotinamide n	4.76	4.49	5.03	2.32	1.84	2.15	2.26	1.18	5.76E-22	1.27E-18	-17.90	up	yes
ENSMUSG00000110105	Gm45844	predicted gene	0.62	0.62	0.60	0.11	0.05	0.10	7.22	2.85	1.11E-20	2.30E-17	-16.64	up	yes
ENSMUSG00000096862	Gm13301	predicted gene	0.03	0.02	0.04	0.91	0.98	0.74	0.03	-4.91	2.40E-19	4.67E-16	-15.33	down	yes
ENSMUSG00000116207	Nnt	nicotinamide n	0	0	0	1.14	2.57	0.79	0.00	-13.87	1.52E-18	2.78E-15	-14.56	down	yes
ENSMUSG00000035299	Mid1	midline 1 [Sou	2.32	0.96	2.87	0.42	0.40	0.39	5.11	2.35	1.96E-18	3.41E-15	-14.47	up	yes
ENSMUSG00000109176	Zfp264	zinc finger pr	0.02	0.03	0.05	0.60	0.76	0.73	0.04	-4.48	1.18E-17	1.95E-14	-13.71	down	yes
ENSMUSG00000058626	Capn11	calpain 11 [So	0.82	1.23	0.98	0.13	0.04	0.11	11.19	3.48	8.60E-17	1.35E-13	-12.87	up	yes
ENSMUSG00000064179	Tnnt1	troponin T1, s	6.33	4.73	4.24	1.88	1.38	1.95	2.94	1.55	4.52E-15	6.78E-12	-11.17	up	yes
ENSMUSG00000098374	Gm28043	predicted gene	1.88	1.13	1.88	3.37	4.24	4.75	0.40	-1.33	1.95E-12	2.72E-09	-8.57	down	yes
ENSMUSG00000084010	Gm13302	predicted gene	0.09	0.09	0.08	1.23	1.11	0.63	0.09	-3.46	1.98E-12	2.72E-09	-8.57	down	yes
ENSMUSG00000031762	Mt2	metallothionei	130.50	115.34	100.21	72.18	66.13	65.60	1.70	0.76	3.77E-12	4.98E-09	-8.30	up	no
ENSMUSG00000118425	Gm50470	predicted gene	0.08	0.09	0.15	1.07	1.08	0.83	0.11	-3.19	4.44E-12	5.64E-09	-8.25	down	yes
ENSMUSG00000118434	Gm13301	predicted gene	0.10	0.12	0.19	2.15	1.88	1.33	0.08	-3.67	6.82E-12	8.34E-09	-8.08	down	yes
ENSMUSG00000035429	Ptprh	protein tyrosi	0.36	0.34	0.47	0.04	0	0.04	14.79	3.89	2.91E-11	3.43E-08	-7.46	up	yes
ENSMUSG00000030413	Pglyrp1	peptidoglycan	6.08	2.37	2.14	0.77	0.89	0.81	4.28	2.10	9.67E-11	1.10E-07	-6.96	up	yes
ENSMUSG00000096256	Gm21093	predicted gene	1.14	0.68	1.17	2.46	2.80	2.93	0.36	-1.45	1.11E-10	1.22E-07	-6.91	down	yes
ENSMUSG00000093909	Gm3883	predicted gene	1.04	0.54	0.91	2.28	2.49	2.49	0.34	-1.54	1.24E-10	1.32E-07	-6.88	down	yes
ENSMUSG00000080893	Ndufa12-ps	NADH:ubiquinon	78.07	94.51	80.70	37.77	53.59	48.85	1.81	0.85	1.81E-10	1.86E-07	-6.73	up	no
ENSMUSG00000054594	Oscar	osteoclast ass	0.80	0.45	1.06	0.08	0	0.05	17.70	4.15	2.23E-10	2.23E-07	-6.65	up	yes
ENSMUSG00000090083	Rnf8	ring finger pr	2.03	1.89	1.96	1.31	1.17	1.17	1.61	0.69	4.96E-10	4.81E-07	-6.32	up	no
ENSMUSG00000095463	Entpd4	ectonucleoside	4.89	4.46	4.34	8.61	7.74	7.24	0.58	-0.79	7.25E-10	6.84E-07	-6.17	down	no
ENSMUSG00000086604	Gm15510	predicted gene	1.18	1.44	0.98	0.15	0.19	0.05	9.02	3.17	1.15E-09	1.05E-06	-5.98	up	yes
ENSMUSG00000008435	Rdh13	retinol dehydr	3.18	2.65	3.00	5.00	5.28	4.74	0.59	-0.77	1.25E-09	1.09E-06	-5.96	down	no
ENSMUSG00000031765	Mt1	metallothionei	116.71	108.43	89.72	72.25	64.05	62.35	1.59	0.66	1.26E-09	1.09E-06	-5.96	up	no
ENSMUSG00000005716	Pvalb	parvalbumin [S	71.28	75.60	55.73	38.38	44.42	41.00	1.64	0.71	1.64E-09	1.39E-06	-5.86	up	no
ENSMUSG00000053985	Zfp14	zinc finger pr	3.48	3.06	3.47	5.61	5.84	6.19	0.57	-0.82	3.36E-09	2.77E-06	-5.56	down	no
ENSMUSG00000107705	Gm45062	predicted gene	0	0.86	0	0	0	0	2878.26	11.49	5.01E-09	4.03E-06	-5.39	up	yes
ENSMUSG00000093996	Fam205a3	family with se	0.83	0.75	0.80	1.88	2.09	1.55	0.43	-1.22	5.45E-09	4.28E-06	-5.37	down	yes
ENSMUSG00000108815	Gm49388	predicted gene	1.70	0	0	0	0	0	5680.79	12.47	8.20E-09	6.30E-06	-5.20	up	yes
ENSMUSG00000091177	Gm15494	predicted gene	0	0	0.07	1.31	1.38	1.66	0.02	-5.98	1.79E-08	1.34E-05	-4.87	down	yes
ENSMUSG00000090996	Gm20458	predicted gene	0	1.61	0	0	0	0	5360.06	12.39	2.85E-08	2.09E-05	-4.68	up	yes
ENSMUSG00000078746	Fam205a4	family with se	0.81	0.73	0.79	1.83	2.01	1.47	0.44	-1.18	3.00E-08	2.15E-05	-4.67	down	yes
ENSMUSG00000053898	Ech1	enoyl coenzyme	16.40	19.51	16.05	10.97	11.15	11.67	1.54	0.62	3.58E-08	2.51E-05	-4.60	up	no
ENSMUSG00000089865	Gm44503	predicted read	0	0.44	0	0	0	0	1481.09	10.53	3.68E-08	2.53E-05	-4.60	up	yes
ENSMUSG00000024620	Pdgfrb	platelet deriv	2.33	2.28	2.77	4.44	4.19	4.14	0.58	-0.79	4.76E-08	3.21E-05	-4.49	down	no
ENSMUSG00000096764	Gm21985	predicted gene	0	0	0	0.11	1.08	0.33	0.00	-12.31	5.95E-08	3.93E-05	-4.41	down	yes
ENSMUSG00000057036	Gm7536	predicted gene	34.94	19.33	40.40	13.49	18.20	11.49	2.19	1.13	1.35E-07	8.75E-05	-4.06	up	yes
ENSMUSG00000116024	Gm49527	predicted gene	0.80	0.78	4.27	0.35	0.36	0.17	6.63	2.73	2.13E-07	1.35E-04	-3.87	up	yes
ENSMUSG00000094066	Fam205a2	family with se	0.76	0.70	0.70	1.67	1.87	1.33	0.44	-1.18	2.23E-07	1.39E-04	-3.86	down	yes
ENSMUSG00000110631	Gm42047	predicted gene	0.28	0.22	0.40	0.83	0.74	0.87	0.37	-1.43	2.33E-07	1.43E-04	-3.85	down	yes
ENSMUSG00000045948	Mrps12	mitochondrial	19.11	27.00	21.04	13.20	13.92	13.62	1.65	0.72	3.28E-07	1.97E-04	-3.71	up	no
ENSMUSG00000066838	Zfp772	zinc finger pr	4.74	3.47	4.72	2.35	2.87	2.51	1.67	0.74	3.41E-07	2.01E-04	-3.70	up	no
ENSMUSG00000026193	Fn1	fibronectin 1	3.05	2.31	3.17	4.53	4.92	4.32	0.62	-0.69	3.75E-07	2.17E-04	-3.66	down	no
ENSMUSG00000114942	Gm49361	predicted gene	0	0.32	0	0	0	0	1061.17	10.05	3.81E-07	2.17E-04	-3.66	up	yes
ENSMUSG00000026678	Rgs5	regulator of G	15.14	13.05	16.15	22.52	22.49	21.27	0.67	-0.58	4.07E-07	2.28E-04	-3.64	down	no
ENSMUSG00000095348	Gm3892	predicted gene	0.28	0.19	0.24	0.85	0.81	0.62	0.31	-1.69	5.23E-07	2.88E-04	-3.54	down	yes
ENSMUSG00000095366	Gm21860	predicted gene	0.26	0.03	0.51	1.88	1.58	2.26	0.14	-2.82	5.51E-07	2.95E-04	-3.53	down	yes
ENSMUSG00000039001	Rps21	ribosomal prot	117.81	170.56	122.35	98.56	72.81	71.47	1.69	0.76	5.54E-07	2.95E-04	-3.53	up	no
ENSMUSG00000014846	Tppp3	tubulin polyme	31.04	26.56	24.12	18.27	21.14	16.75	1.46	0.54	6.26E-07	3.28E-04	-3.48	up	no
ENSMUSG00000031760	Mt3	metallothionei	115.15	169.86	114.17	93.37	73.29	73.01	1.67	0.74	7.25E-07	3.74E-04	-3.43	up	no
ENSMUSG00000102440	Pcdhga9	protocadherin	4.54	3.88	5.35	8.60	10.32	6.32	0.55	-0.87	8.91E-07	4.53E-04	-3.34	down	no
ENSMUSG00000090223	Pcp4	Purkinje cell	299.85	283.63	249.81	204.31	216.62	211.70	1.32	0.40	1.05E-06	5.26E-04	-3.28	up	no
ENSMUSG00000073616	Cops9	COP9 signaloso	27.41	35.93	26.67	20.84	19.28	17.96	1.55	0.63	1.14E-06	5.60E-04	-3.25	up	no
ENSMUSG00000079641	Rpl39	ribosomal prot	30.67	42.85	29.85	24.30	19.48	19.05	1.65	0.72	1.36E-06	6.60E-04	-3.18	up	no
ENSMUSG00000060636	Rpl35a	ribosomal prot	35.13	47.69	33.95	26.83	25.76	24.69	1.51	0.60	1.39E-06	6.63E-04	-3.18	up	no
ENSMUSG00000090733	Rps27	ribosomal prot	85.14	118.12	84.66	67.86	60.90	57.26	1.55	0.63	1.53E-06	7.21E-04	-3.14	up	no
ENSMUSG00000109205	Gm44954	predicted gene	0.47	0.02	0.25	3.00	3.30	3.65	0.07	-3.74	1.73E-06	8.04E-04	-3.09	down	yes
ENSMUSG00000020182	Ddc	dopa decarboxy	5.97	3.45	5.36	2.26	3.62	2.20	1.83	0.87	1.90E-06	8.71E-04	-3.06	up	no
ENSMUSG00000021290	Atp5mpl	ATP synthase m	40.53	55.23	40.57	31.56	28.23	29.29	1.53	0.61	2.29E-06	1.03E-03	-2.99	up	no
ENSMUSG00000057863	Rpl36	ribosomal prot	107.92	147.29	117.87	91.16	78.13	69.30	1.56	0.64	2.48E-06	1.10E-03	-2.96	up	no
ENSMUSG00000062328	Rpl17	ribosomal prot	49.88	38.22	28.55	25.89	24.05	22.23	1.62	0.69	2.68E-06	1.17E-03	-2.93	up	no
ENSMUSG00000095258	Gm10593	predicted gene	0.22	0.17	0.17	0.58	0.65	0.55	0.32	-1.66	2.70E-06	1.17E-03	-2.93	down	yes
ENSMUSG00000017734	Dbndd2	dysbindin (dys	38.24	32.40	33.63	25.93	29.21	26.91	1.27	0.35	2.89E-06	1.24E-03	-2.91	up	no
ENSMUSG00000029761	Cald1	caldesmon 1 [S	2.11	1.58	1.78	2.80	3.00	2.76	0.64	-0.64	3.09E-06	1.31E-03	-2.88	down	no
ENSMUSG00000033208	S100b	S100 protein,	94.35	101.20	94.06	74.56	78.09	72.10	1.29	0.37	3.16E-06	1.32E-03	-2.88	up	no
ENSMUSG00000022132	Cldn10	claudin 10 [So	14.45	15.59	12.10	9.85	8.77	10.01	1.47	0.56	3.20E-06	1.32E-03	-2.88	up	no
ENSMUSG00000041607	Mbp	myelin basic p	330.82	371.13	332.77	250.74	288.66	256.33	1.30	0.38	3.27E-06	1.33E-03	-2.88	up	no
ENSMUSG00000103928	Gm37893	predicted gene	0.22	0.20	0.21	0.79	0.81	0.63	0.28	-1.81	3.39E-06	1.36E-03	-2.87	down	yes
ENSMUSG00000067288	Rps28	ribosomal prot	16.01	26.79	18.18	13.11	11.38	11.45	1.70	0.76	3.44E-06	1.36E-03	-2.87	up	no
ENSMUSG00000108348	Gm42372	predicted gene	22.01	19.91	21.16	14.98	16.50	17.76	1.28	0.36	3.45E-06	1.36E-03	-2.87	up	no
ENSMUSG00000074358	Ccdc61	coiled-coil do	1.98	2.49	1.83	1.21	1.05	0.95	1.96	0.97	3.75E-06	1.46E-03	-2.84	up	no
ENSMUSG00000068740	Celsr2	cadherin, EGF	9.09	7.71	10.27	13.22	13.13	13.59	0.68	-0.56	3.92E-06	1.50E-03	-2.82	down	no
ENSMUSG00000035783	Acta2	actin, alpha 2	7.64	6.93	8.37	11.93	15.23	10.93	0.60	-0.73	4.00E-06	1.52E-03	-2.82	down	no
ENSMUSG00000080921	Rpl38-ps2	ribosomal prot	39.19	65.07	36.40	26.51	22.80	20.26	2.02	1.02	4.27E-06	1.60E-03	-2.80	up	yes
ENSMUSG00000033061	Resp18	regulated endo	94.88	105.43	88.40	68.38	78.25	66.66	1.35	0.44	4.56E-06	1.69E-03	-2.77	up	no
ENSMUSG00000118603	BX666061	protein FAM205	0.16	0.14	0.17	0.64	0.67	0.59	0.25	-1.97	4.61E-06	1.69E-03	-2.77	down	yes
ENSMUSG00000017778	Cox7c	cytochrome c o	80.76	108.54	84.32	70.89	52.87	55.54	1.53	0.61	6.31E-06	2.29E-03	-2.64	up	no
ENSMUSG00000082414	Gm13303	predicted gene	0.42	0.22	0.36	1.32	1.15	1.11	0.28	-1.85	7.16E-06	2.55E-03	-2.59	down	yes
ENSMUSG00000049517	Rps23	ribosomal prot	140.01	184.03	134.70	111.34	105.24	103.87	1.43	0.52	7.18E-06	2.55E-03	-2.59	up	no

Table S2B: Selection of genes altered in E4H vs E3H mice. FPKM values of genes in individual mice (indicated in underscored numeral), along with fold change (fc) value, p value adjusted for false discovery rate of 0.05 (q value, qval) are presented. N=3 mice/group.

gene_id	gene_name	Description	FPKM.E4H_1	FPKM.E4H_2	FPKM.E4H_3	FPKM.E3H_1	FPKM.E3H_2	FPKM.E3H_3	fc	log2 (fc)	pval	qval	log10 (qval)	regulation	significant
ENSMUSG00000021091	Serpina3n	serine (or cys	71.58	62.15	75.81	11.81	13.33	11.56	5.71	2.51	2.23E-214	7.41E-210	-209.13	up	yes
ENSMUSG00000096768	Gm47283	predicted gene	0.09	0.04	0.07	5.77	4.18	3.58	0.02	-6.06	1.78E-127	2.96E-123	-122.53	down	yes
ENSMUSG00000095562	Erdr1	erythroid diff	0.12	0.17	0.19	14.44	10.26	8.01	0.01	-6.12	6.13E-109	6.80E-105	-104.17	down	yes
ENSMUSG00000073643	Wdfy1	WD repeat and	2.30	2.60	2.34	6.75	8.29	8.25	0.31	-1.69	2.87E-66	2.39E-62	-61.62	down	yes
ENSMUSG00000107705	Gm45062	predicted gene	7.60	6.71	4.96	0	0	0	64232.1	15.97	8.01E-32	5.34E-28	-27.27	up	yes
ENSMUSG00000006154	Eps8l1	EPS8-like 1 [S	0.98	1.17	0.69	0.14	0.12	0.14	7.16	2.84	3.09E-28	1.72E-24	-23.77	up	yes
ENSMUSG00000105340	Gm42878	predicted gene	0	0	0	1.55	1.59	1.39	0.00	-13.88	5.02E-22	2.39E-18	-17.62	down	yes
ENSMUSG00000004798	Ulk2	unc-51 like ki	11.95	11.62	12.92	18.43	18.50	20.04	0.64	-0.64	1.86E-18	7.74E-15	-14.11	down	no
ENSMUSG00000076612	Ighg2c	immunoglobulin	0	0	0	3.10	2.67	2.57	0.00	-14.76	2.72E-18	1.01E-14	-14.00	down	yes
ENSMUSG00000008435	Rdh13	retinol dehydr	2.68	3.10	2.81	5.00	5.28	4.74	0.57	-0.80	2.84E-16	9.44E-13	-12.03	down	no
ENSMUSG00000054594	Oscar	osteoclast ass	0.94	1.00	0.91	0.08	0	0.05	21.79	4.45	1.19E-15	3.60E-12	-11.44	up	yes
ENSMUSG00000087467	Gm13601	predicted gene	0.05	0	0.14	3.28	3.18	4.42	0.02	-5.85	4.75E-15	1.32E-11	-10.88	down	yes
ENSMUSG00000024213	Nudt3	nudix (nucleot	57.00	56.52	58.46	45.94	45.60	46.04	1.25	0.32	2.55E-12	6.54E-09	-8.18	up	no
ENSMUSG00000035429	Ptprh	protein tyrosi	0.34	0.45	0.36	0.04	0	0.04	14.53	3.86	8.61E-12	2.05E-08	-7.69	up	yes
ENSMUSG00000076617	Ighm	immunoglobulin	8.10	9.99	8.31	14.22	16.53	14.22	0.59	-0.77	1.77E-11	3.92E-08	-7.41	down	no
ENSMUSG00000031562	Dctd	dCMP deaminase	1.11	1.06	0.94	2.28	2.54	2.11	0.45	-1.15	2.44E-11	5.08E-08	-7.29	down	yes
ENSMUSG00000106874	Gm20186	predicted gene	0	0.01	0	1.36	1.43	1.33	0.00	-8.95	4.68E-11	9.16E-08	-7.04	down	yes
ENSMUSG00000032667	Pon2	paraoxonase 2	4.97	4.30	4.26	6.44	6.98	6.70	0.67	-0.57	8.12E-11	1.50E-07	-6.82	down	no
ENSMUSG00000086604	Gm15510	predicted gene	1.10	1.33	1.27	0.15	0.19	0.05	9.27	3.21	9.25E-11	1.62E-07	-6.79	up	yes
ENSMUSG00000069049	Eif2s3y	eukaryotic tra	0.02	0	9.44	0	0	0	31543.8	14.95	1.04E-10	1.74E-07	-6.76	up	yes
ENSMUSG00000058706	0610030E20Rik	RIKEN cDNA 061	2.46	2.87	2.24	1.20	1.39	1.58	1.82	0.86	1.29E-10	2.04E-07	-6.69	up	no
ENSMUSG00000001930	Vwf	Von Willebrand	0.78	0.72	0.62	1.34	1.33	1.11	0.56	-0.84	2.01E-10	3.04E-07	-6.52	down	no
ENSMUSG00000052861	Dnah6	dynein, axonem	0.60	0.62	0.53	0.17	0.33	0.23	2.40	1.27	2.37E-10	3.43E-07	-6.46	up	yes
ENSMUSG00000050071	Bex1	brain expresse	32.21	28.37	34.19	22.91	22.05	20.52	1.45	0.53	1.70E-09	2.36E-06	-5.63	up	no
ENSMUSG00000103707	Pcdha6	protocadherin	1.27	1.02	0.65	2.70	2.35	1.99	0.42	-1.26	2.10E-09	2.79E-06	-5.55	down	yes
ENSMUSG00000089862	Umad1	UMAP1-MVP12 as	5.91	6.04	6.76	8.65	8.89	9.51	0.69	-0.53	5.61E-09	7.11E-06	-5.15	down	no
ENSMUSG00000019773	Fbxo5	F-box protein	1.63	2.06	1.27	0.53	0.53	0.60	2.98	1.58	5.77E-09	7.11E-06	-5.15	up	yes
ENSMUSG00000095366	Gm21860	predicted gene	0.32	0.56	0.50	1.88	1.58	2.26	0.24	-2.04	1.47E-08	1.75E-05	-4.76	down	yes
ENSMUSG00000029994	Anxa4	annexin A4 [Sc	2.01	2.00	2.01	1.12	1.21	1.25	1.68	0.75	1.67E-08	1.91E-05	-4.72	up	no
ENSMUSG00000107927	Gm44090	predicted gene	0	0.03	0.02	0.73	0.38	0.26	0.03	-4.99	1.79E-08	1.98E-05	-4.70	down	yes
ENSMUSG00000002477	Snrpd1	small nuclear	16.13	16.04	15.47	11.27	12.77	10.38	1.38	0.47	2.40E-08	2.58E-05	-4.59	up	no
ENSMUSG00000075514	Gm13375	predicted gene	6.02	6.85	6.22	11.01	11.13	11.52	0.57	-0.82	4.70E-08	4.89E-05	-4.31	down	no
ENSMUSG00000042750	Bex2	brain expresse	175.32	160.95	175.57	140.71	143.53	129.31	1.24	0.31	6.19E-08	6.19E-05	-4.21	up	no
ENSMUSG00000037860	Aim2	absent in mela	0.98	0.99	0.92	0.43	0.41	0.33	2.46	1.30	6.32E-08	6.19E-05	-4.21	up	yes
ENSMUSG00000046952	Gm5815	predicted pseu	0.98	1.48	1.48	0.38	0.16	0.19	5.40	2.43	6.63E-08	6.30E-05	-4.20	up	yes
ENSMUSG00000035781	R3hdm4	R3H domain con	26.85	27.70	27.94	36.22	34.65	34.56	0.78	-0.35	7.25E-08	6.42E-05	-4.19	down	no
ENSMUSG00000073411	H2-D1	histocompatibi	17.17	16.97	17.06	22.04	24.08	21.90	0.75	-0.41	7.27E-08	6.42E-05	-4.19	down	no
ENSMUSG00000091177	Gm15494	predicted gene	0.05	0.08	0.19	1.31	1.38	1.66	0.08	-3.72	7.36E-08	6.42E-05	-4.19	down	yes
ENSMUSG00000079494	Nat8f5	N-acetyltransf	0.22	0.20	0.18	1.15	1.39	1.08	0.17	-2.60	7.52E-08	6.42E-05	-4.19	down	yes
ENSMUSG00000091228	Gm20390	predicted gene	0	0	0	0	1.19	0	0.00	-11.95	2.22E-07	1.85E-04	-3.73	down	yes
ENSMUSG00000042406	Atf4	activating tra	46.25	48.03	46.19	36.77	41.97	37.63	1.21	0.27	3.38E-07	2.74E-04	-3.56	up	no
ENSMUSG00000064345	mt-Nd2	mitochondriall	4352.43	4282.67	4065.09	3738.38	3594.01	3509.17	1.17	0.23	4.75E-07	3.76E-04	-3.42	up	no
ENSMUSG00000030123	Plxnd1	plexin D1 [Sou	7.90	8.62	8.10	11.70	10.20	11.32	0.74	-0.43	6.68E-07	5.17E-04	-3.29	down	no
ENSMUSG00000060962	Dmkn	dermokine [Sou	0.51	0.50	0.27	1.31	1.37	1.13	0.33	-1.58	9.15E-07	6.92E-04	-3.16	down	yes
ENSMUSG00000058838	Rps27a-ps2	ribosomal prot	22.37	24.08	23.02	37.58	32.38	36.74	0.65	-0.62	9.48E-07	7.01E-04	-3.15	down	no
ENSMUSG00000024104	Washc2	WASH complex s	14.42	13.95	14.14	11.32	12.07	12.44	1.19	0.25	1.09E-06	7.88E-04	-3.10	up	no
ENSMUSG00000079012	Serpina3m	serine (or cys	1.87	2.05	2.06	0.05	0.11	0	36.53	5.19	1.49E-06	1.05E-03	-2.98	up	yes
ENSMUSG00000020893	Per1	period circadi	10.27	10.11	6.92	14.36	13.65	13.02	0.67	-0.59	1.55E-06	1.07E-03	-2.97	down	no
ENSMUSG00000030126	Tmcc1	transmembrane	4.80	4.71	4.64	3.73	4.03	4.15	1.19	0.25	2.17E-06	1.47E-03	-2.83	up	no
ENSMUSG00000073418	C4b	complement com	7.70	7.55	8.42	9.53	11.14	10.75	0.75	-0.41	2.35E-06	1.54E-03	-2.81	down	no
ENSMUSG00000031375	Bgn	biglycan [Sour	3.09	3.12	2.74	4.45	4.52	3.89	0.70	-0.52	2.35E-06	1.54E-03	-2.81	down	no
ENSMUSG00000062995	Ica1	islet cell aut	10.11	10.18	11.20	12.92	14.56	15.11	0.74	-0.44	2.50E-06	1.60E-03	-2.80	down	no
ENSMUSG00000026278	Bok	BCL2-related c	17.16	16.52	19.11	13.75	13.29	13.42	1.30	0.38	2.69E-06	1.69E-03	-2.77	up	no
ENSMUSG00000020180	Snrpd3	small nuclear	15.69	16.54	17.95	13.04	13.67	13.86	1.24	0.31	3.24E-06	2.00E-03	-2.70	up	no
ENSMUSG00000064340	mt-Tl1	mitochondriall	491.08	468.04	542.37	417.66	341.17	329.94	1.38	0.46	3.54E-06	2.14E-03	-2.67	up	no
ENSMUSG00000052135	Foxo6	forkhead box C	2.48	2.54	2.67	4.72	4.16	3.71	0.61	-0.71	4.24E-06	2.52E-03	-2.60	down	no
ENSMUSG00000026234	Ncl	nucleolin [Sou	26.84	26.43	29.08	21.82	23.10	24.77	1.18	0.24	4.99E-06	2.91E-03	-2.54	up	no
ENSMUSG00000090223	Pcp4	Purkinje cell	240.40	234.77	243.38	204.31	216.62	211.70	1.14	0.18	5.28E-06	3.03E-03	-2.52	up	no
ENSMUSG00000063681	Crb1	crumbs family	0.18	0.14	0.12	0.04	0.06	0.05	2.76	1.47	6.64E-06	3.75E-03	-2.43	up	yes
ENSMUSG00000029819	Npy	neuropeptide Y	61.60	64.20	52.98	47.88	40.65	44.60	1.34	0.43	7.57E-06	4.20E-03	-2.38	up	no
ENSMUSG00000079592	C1qtnf5	C1q and tumor	2.36	2.10	2.42	1.02	1.39	0.95	2.04	1.03	7.70E-06	4.20E-03	-2.38	up	yes
ENSMUSG00000064341	mt-Nd1	mitochondriall	6903.28	6695.82	6581.76	6129.84	5745.46	5553.22	1.16	0.21	8.39E-06	4.51E-03	-2.35	up	no
ENSMUSG00000030108	Slc6a13	solute carrier	3.41	2.86	3.30	4.42	5.17	4.83	0.66	-0.59	1.04E-05	5.48E-03	-2.26	down	no
ENSMUSG00000065947	mt-Nd4l	mitochondriall	2405.55	2483.45	2460.75	2141.76	1889.46	2167.16	1.19	0.25	1.19E-05	6.21E-03	-2.21	up	no
ENSMUSG00000064344	mt-Tm	mitochondriall	275.00	257.17	248.62	197.29	201.06	189.98	1.33	0.41	1.41E-05	7.21E-03	-2.14	up	no
ENSMUSG00000067736	Gm10222	predicted gene	2394.69	2497.58	2478.33	2157.49	1904.45	2170.13	1.18	0.24	1.51E-05	7.64E-03	-2.12	up	no
ENSMUSG00000103793	Pcdhga6	protocadherin	6.11	7.91	6.74	9.76	8.76	11.20	0.70	-0.52	1.56E-05	7.70E-03	-2.11	down	no
ENSMUSG00000007594	Hapln4	hyaluronan and	30.21	32.64	29.75	25.79	27.84	25.67	1.17	0.22	1.59E-05	7.70E-03	-2.11	up	no
ENSMUSG00000051359	Ncald	neurocalcin de	61.14	63.72	64.82	50.49	55.68	58.40	1.15	0.20	1.60E-05	7.70E-03	-2.11	up	no
ENSMUSG00000087412	Gm15501	predicted pseu	5.84	6.23	6.44	11.48	9.02	10.22	0.60	-0.73	1.64E-05	7.82E-03	-2.11	down	no
ENSMUSG00000044676	Zfp612	zinc finger pr	8.10	7.75	7.43	9.35	11.05	9.63	0.77	-0.37	1.70E-05	7.97E-03	-2.10	down	no
ENSMUSG00000028790	Khdrbs1	KH domain cont	20.49	20.34	19.49	16.61	18.04	17.88	1.15	0.20	1.73E-05	7.98E-03	-2.10	up	no
ENSMUSG00000046056	Sbsn	suprabasin [Sc	0.90	1.16	1.00	1.84	1.87	1.66	0.57	-0.81	2.00E-05	9.13E-03	-2.04	down	no
ENSMUSG00000062132	Arhgap33os	Rho GTPase act	0.15	0.17	0.25	0	0	0.03	20.17	4.33	2.06E-05	9.26E-03	-2.03	up	yes
ENSMUSG00000107585	3300002P13Rik	RIKEN cDNA 330	21.90	22.40	23.67	37.03	29.43	29.61	0.71	-0.50	2.17E-05	9.62E-03	-2.02	down	no
ENSMUSG00000087263	Gm15726	predicted gene	0	0.04	0.01	1.00	0.50	0.79	0.03	-5.30	2.66E-05	1.16E-02	-1.93	down	yes
ENSMUSG00000064179	Tnnt1	troponin T1, s	2.74	2.66	4.49	1.88	1.38	1.95	1.90	0.92	2.72E-05	1.18E-02	-1.93	up	no
ENSMUSG00000045962	Wnk1	WNK lysine def	7.28	7.14	7.02	8.43	8.55	8.68	0.84	-0.26	3.14E-05	1.34E-02	-1.87	down	no
ENSMUSG00000029648	Flt1	FMS-like tyros	2.91	3.08	3.00	3.78	3.70	3.92	0.79	-0.34	3.55E-05	1.50E-02	-1.83	down	no
ENSMUSG00000051579	Tceal8	transcription	15.98	17.02	17.39	14.12	14.11	13.59	1.20	0.27	5.25E-05	2.18E-02	-1.66	up	no
ENSMUSG00000030680	Pagr1a	PAXIP1 associa	0.39	0.14	0.12	2.32	1.44	0.22	0.16	-2.61	5.30E-05	2.18E-02	-1.66	down	yes
ENSMUSG00000078578	Ube2d3	ubiquitin-conj	10.86	10.76	11.29	9.54	9.64	10.05	1.13	0.17	6.44E-05	2.61E-02	-1.58	up	no
ENSMUSG00000074923	Pak6	p21 (RAC1) act	9.98	9.98	10.08	13.26	12.20	12.30	0.80	-0.33	6.69E-05	2.68E-02	-1.57	down	no
ENSMUSG00000006362	Cbfa2t3	CBFA2/RUNX1 tr	1.48	1.52	1.48	2.15	2.10	1.94	0.72	-0.47	7.24E-05	2.87E-02	-1.54	down	no
ENSMUSG00000024565	Sall3	spalt like tra	0.25	0.19	0.37	0.54	0.54	0.62	0.48	-1.06	7.55E-05	2.96E-02	-1.53	down	yes
ENSMUSG00000071984	Fndc1	fibronectin ty	0.30	0.30	0.30	0.51	0.58	0.47	0.58	-0.79	7.85E-05	3.04E-02	-1.52	down	no
ENSMUSG00000028341	Nr4a3	nuclear recept	4.65	4.49	3.11	6.46	5.58	5.86	0.68	-0.55	8.32E-05	3.18E-02	-1.50	down	no
ENSMUSG00000085105	Gm12758	predicted gene	0.71	0.55	0.33	1.42	0.78	1.25	0.46	-1.12	8.65E-05	3.27E-02	-1.49	down	yes
ENSMUSG00000048550	Thnsl1	threonine synt	4.92	4.68	5.24	3.30	4.28	3.77	1.31	0.39	9.07E-05	3.39E-02	-1.47	up	no
ENSMUSG00000036915	Kirrel2	kirre like nep	2.93	1.81	2.23	1.51	1.28	1.55	1.61	0.68	9.25E-05	3.42E-02	-1.47	up	no
ENSMUSG00000075552	Cyp3a41b	cytochrome P45	0	0	0	0.09	0.20	0.03	0.00	-10.06	1.08E-04	3.96E-02	-1.40	down	yes
ENSMUSG00000017754	Pltp	phospholipid t	25.27	25.68	20.70	29.82	31.70	30.28	0.78	-0.36	1.17E-04	4.20E-02	-1.38	down	no
ENSMUSG00000111995	Gm48697	predicted gene	0	0	0	0.04	0.08	0.05	0.00	-9.16	1.17E-04	4.20E-02	-1.38	down	yes
ENSMUSG00000062593	Gm49339	predicted gene	0	0.02	0	0.10	0.25	0.18	0.03	-5.12	1.18E-04	4.20E-02	-1.38	down	yes
ENSMUSG00000000197	Nalcn	sodium leak ch	7.13	7.38	7.29	5.71	6.34	6.75	1.16	0.21	1.29E-04	4.52E-02	-1.34	up	no

ENSMUSG00000029634	Rnf6	ring finger pr	15.80	16.23	15.68	12.45	13.15	14.69	1.18	0.24	1.35E-04	4.67E-02	-1.33 up	no
ENSMUSG00000036644	Tbc1d9b	TBC1 domain fa	27.13	26.82	29.47	32.91	33.85	33.21	0.83	-0.26	1.36E-04	4.67E-02	-1.33 down	no
ENSMUSG00000001774	Chordc1	cysteine and h	9.90	10.40	9.98	8.35	9.24	8.85	1.15	0.20	1.41E-04	4.81E-02	-1.32 up	no
ENSMUSG00000042831	Alkbh6	alkB homolog 6	5.17	5.54	6.09	7.65	7.76	7.20	0.74	-0.43	1.44E-04	4.85E-02	-1.31 down	no

Table S2C: Selection of genes robustly altered in E4H vs E2H mice. FPKM values of genes in individual mice (indicated in underscored numeral), along with fold change (fc) value, p value adjusted for false discovery rate of 0.05 (q value, qval) are presented. N=3 mice/group.

gene_id	gene_name	Description	FPKM. E4H_1	FPKM.E 4H_2	FPKM. E4H_3	FPKM.E 2H_1	FPKM.E2 H_2	FPKM.E 2H_3	fc	log2 (fc)	pval	qval	log10 (qval)	regula tion	signif icant
ENSMUSG00000093954	Gm16867	predicted gene,	2.53	2.22	2.10	0.07	0.04	0.07	39.97	5.32	9.91E-130	3.29E-125	-124.48	up	yes
ENSMUSG00000096768	Gm47283	predicted gene,	0.09	0.04	0.07	7.73	3.26	5.04	0.01	-6.30	2.01E-97	3.33E-93	-92.48	down	yes
ENSMUSG00000095562	Erdr1	erythroid diffe	0.12	0.17	0.19	17.85	7.97	11.38	0.01	-6.30	7.07E-96	7.81E-92	-91.11	down	yes
ENSMUSG00000021091	Serpina3n	serine (or cyst	71.58	62.15	75.81	14.64	13.27	12.50	5.19	2.37	2.60E-95	2.15E-91	-90.67	up	yes
ENSMUSG00000098975	Gm27177	predicted gene	2.16	1.95	1.33	0.06	0.12	0.07	21.40	4.42	5.04E-58	3.34E-54	-53.48	up	yes
ENSMUSG00000109209	Gm45104	predicted gene	2.39	2.20	2.43	0.17	0.26	0.24	10.57	3.40	6.36E-53	3.51E-49	-48.45	up	yes
ENSMUSG00000073643	Wdfy1	WD repeat and F	2.30	2.60	2.34	7.96	6.64	7.02	0.33	-1.58	2.27E-47	1.07E-43	-42.97	down	yes
ENSMUSG00000118458	Gm10599	predicted pseud	2.91	3.87	1.98	0.12	0.10	0.09	27.98	4.81	1.09E-44	4.51E-41	-40.35	up	yes
ENSMUSG00000090338	Gm17081	predicted gene	2.53	3.36	1.71	0.07	0.07	0.06	38.38	5.26	1.30E-43	4.80E-40	-39.32	up	yes
ENSMUSG00000091542	Gm17167	predicted gene	1.18	1.79	0.87	0.08	0.05	0.05	21.21	4.41	3.86E-37	1.28E-33	-32.89	up	yes
ENSMUSG00000095675	Ccl21b	chemokine (C-C	4.18	4.66	5.32	0.14	0.10	0.01	54.75	5.77	2.84E-36	8.57E-33	-32.07	up	yes
ENSMUSG00000073878	Gm13304	predicted gene	4.45	4.42	4.45	0.15	0.14	0.03	41.49	5.37	1.10E-33	3.03E-30	-29.52	up	yes
ENSMUSG00000073879	Gm5859	predicted pseud	1.82	2.64	1.51	0.12	0.07	0.09	21.45	4.42	2.10E-33	5.36E-30	-29.27	up	yes
ENSMUSG00000096596	Gm10591	predicted gene	4.18	3.85	3.96	0.10	0.08	0.03	57.66	5.85	2.30E-33	5.44E-30	-29.26	up	yes
ENSMUSG00000094065	Ccl21d	chemokine (C-C	3.75	2.80	3.40	0.11	0.10	0.02	42.29	5.40	1.42E-26	3.13E-23	-22.50	up	yes
ENSMUSG00000097842	9330104G04Ri	RIKEN cDNA 9330	0.22	0.31	0.30	1.37	1.56	1.63	0.18	-2.46	5.88E-24	1.22E-20	-19.92	down	yes
ENSMUSG00000079012	Serpina3m	serine (or cyst	1.87	2.05	2.06	0.03	0	0	175.63	7.46	6.24E-24	1.22E-20	-19.92	up	yes
ENSMUSG00000035299	Mid1	midline 1 [Sour	0.33	0.32	0.21	2.32	0.96	2.87	0.14	-2.82	3.26E-21	6.01E-18	-17.22	down	yes
ENSMUSG00000096862	Gm13301	predicted gene	1.01	1.33	0.67	0.03	0.02	0.04	34.56	5.11	7.12E-21	1.24E-17	-16.91	up	yes
ENSMUSG00000110105	Gm45844	predicted gene	0.10	0.12	0.05	0.62	0.62	0.60	0.15	-2.74	3.89E-19	6.45E-16	-15.19	down	yes
ENSMUSG00000052861	Dnah6	dynein, axonema	0.60	0.62	0.53	0.18	0.09	0.11	4.64	2.21	5.03E-19	7.93E-16	-15.10	up	yes
ENSMUSG00000076612	Ighg2c	immunoglobulin	0	0	0	2.32	4.23	2.57	0.00	-14.89	1.40E-18	2.11E-15	-14.68	down	yes
ENSMUSG00000022066	Entpd4b	ectonucleoside	8.97	8.60	9.27	5.18	4.64	5.10	1.80	0.85	2.08E-18	3.00E-15	-14.52	up	no
ENSMUSG00000078897	Gm4724	predicted gene	0.60	0.70	0.65	2.28	1.98	2.06	0.31	-1.69	6.34E-17	8.76E-14	-13.06	down	yes
ENSMUSG00000087467	Gm13601	predicted gene	0.05	0	0.14	3.71	4.40	3.63	0.02	-5.96	2.02E-16	2.68E-13	-12.57	down	yes
ENSMUSG00000084010	Gm13302	predicted gene	1.11	1.49	0.79	0.09	0.09	0.08	12.60	3.66	6.93E-16	8.83E-13	-12.05	up	yes
ENSMUSG00000118434	Gm13301	predicted gene	2.01	2.44	1.66	0.10	0.12	0.19	14.54	3.86	3.69E-15	4.54E-12	-11.34	up	yes
ENSMUSG00000093909	Gm3883	predicted gene	2.93	3.31	2.55	1.04	0.54	0.91	3.54	1.82	6.87E-15	8.13E-12	-11.09	up	yes
ENSMUSG00000118425	Gm50470	predicted gene,	1.20	1.44	0.85	0.08	0.09	0.15	10.70	3.42	9.22E-15	1.05E-11	-10.98	up	yes
ENSMUSG00000096256	Gm21093	predicted gene,	3.60	3.45	2.71	1.14	0.68	1.17	3.26	1.71	1.35E-14	1.49E-11	-10.83	up	yes
ENSMUSG00000053985	Zfp14	zinc finger pro	6.31	6.39	6.33	3.48	3.06	3.47	1.90	0.93	1.82E-14	1.94E-11	-10.71	up	no
ENSMUSG00000095463	Entpd4	ectonucleoside	8.03	7.66	7.99	4.89	4.46	4.34	1.73	0.79	2.36E-14	2.45E-11	-10.61	up	no
ENSMUSG00000058626	Capn11	calpain 11 [Sou	0.05	0.08	0.18	0.82	1.23	0.98	0.10	-3.29	9.79E-14	9.84E-11	-10.01	down	yes
ENSMUSG00000094066	Fam205a2	family with seq	1.86	2.14	1.64	0.76	0.70	0.70	2.61	1.39	3.50E-12	3.41E-09	-8.47	up	yes
ENSMUSG00000030413	Pglyrp1	peptidoglycan r	0.71	0.69	0.71	6.08	2.37	2.14	0.20	-2.32	1.10E-11	1.04E-08	-7.98	down	yes
ENSMUSG00000093996	Fam205a3	family with seq	1.93	2.10	1.66	0.83	0.75	0.80	2.39	1.26	2.42E-11	2.23E-08	-7.65	up	yes
ENSMUSG00000031562	Dctd	dCMP deaminase	1.11	1.06	0.94	2.51	2.35	2.02	0.45	-1.14	7.45E-11	6.68E-08	-7.18	down	yes
ENSMUSG00000078746	Fam205a4	family with seq	1.87	2.02	1.60	0.81	0.73	0.79	2.35	1.23	1.10E-10	9.60E-08	-7.02	up	yes
ENSMUSG00000095348	Gm3892	predicted gene	0.98	1.30	0.72	0.28	0.19	0.24	4.23	2.08	1.17E-10	9.98E-08	-7.00	up	yes
ENSMUSG00000032667	Pon2	paraoxonase 2 [	4.97	4.30	4.26	7.07	7.92	7.13	0.61	-0.71	1.21E-10	1.01E-07	-7.00	down	no
ENSMUSG00000116207	Nnt	nicotinamide nu	3.53	0.35	3.17	0	0	0	23504.15	14.52	2.18E-10	1.76E-07	-6.75	up	yes
ENSMUSG00000079494	Nat8f5	N-acetyltransfe	0.22	0.20	0.18	1.16	1.39	1.65	0.14	-2.81	2.92E-10	2.31E-07	-6.64	down	yes
ENSMUSG00000021171	Esyt2	extended synapt	5.71	5.35	5.52	3.66	3.11	3.69	1.58	0.66	4.17E-10	3.21E-07	-6.49	up	no
ENSMUSG00000108815	Gm49388	predicted gene,	0	0	0	1.70	0	0	0.00	-12.47	5.42E-10	4.08E-07	-6.39	down	yes
ENSMUSG00000068457	Uty	ubiquitously tr	0	0	1.14	0	0	0	3787.61	11.89	1.13E-09	8.36E-07	-6.08	up	yes
ENSMUSG00000106874	Gm20186	predicted gene,	0	0.01	0	1.28	0.48	0.94	0.00	-8.33	5.08E-09	3.66E-06	-5.44	down	yes
ENSMUSG00000089945	Pakap	paralemmin A ki	2.38	1.80	1.23	0.87	0.39	0.57	2.96	1.56	8.70E-09	6.14E-06	-5.21	up	yes
ENSMUSG00000090996	Gm20458	predicted gene	0	0	0	0	1.61	0	0.00	-12.39	9.26E-09	6.39E-06	-5.19	down	yes
ENSMUSG00000046952	Gm5815	predicted pseud	0.98	1.48	1.48	0.19	0.07	0.29	7.19	2.85	1.04E-08	7.02E-06	-5.15	up	yes
ENSMUSG00000089865	Gm44503	predicted readt	0	0	0	0	0.44	0	0.00	-10.53	1.19E-08	7.90E-06	-5.10	down	yes
ENSMUSG00000058838	Rps27a-ps2	ribosomal prote	22.37	24.08	23.02	34.46	48.08	43.46	0.55	-0.86	1.48E-08	9.59E-06	-5.02	down	no
ENSMUSG00000091228	Gm20390	predicted gene	0	0	0	1.67	0	0	0.00	-12.44	1.91E-08	1.22E-05	-4.91	down	yes
ENSMUSG00000021647	Cartpt	CART prepropept	4.96	4.52	6.05	9.10	8.61	11.30	0.54	-0.90	3.90E-08	2.44E-05	-4.61	down	no
ENSMUSG00000114942	Gm49361	predicted gene,	0	0	0	0	0.32	0	0.00	-10.05	4.06E-08	2.50E-05	-4.60	down	yes
ENSMUSG00000095258	Gm10593	predicted gene	0.71	0.87	0.51	0.22	0.17	0.17	3.72	1.90	4.17E-08	2.52E-05	-4.60	up	yes
ENSMUSG00000048108	Tmem72	transmembrane p	0.69	0.51	0.36	0.24	0.19	0.19	2.54	1.35	4.41E-08	2.61E-05	-4.58	up	yes
ENSMUSG00000108348	Gm42372	predicted gene,	16.06	16.04	15.60	22.01	19.91	21.16	0.76	-0.40	5.27E-08	3.07E-05	-4.51	down	no
ENSMUSG00000085105	Gm12758	predicted gene	0.71	0.55	0.33	1.39	1.17	1.50	0.39	-1.35	5.67E-08	3.24E-05	-4.49	down	yes
ENSMUSG00000045948	Mrps12	mitochondrial r	11.85	13.31	12.80	19.11	27.00	21.04	0.57	-0.82	8.78E-08	4.93E-05	-4.31	down	no
ENSMUSG00000109176	Zfp264	zinc finger pro	0.74	0.44	0.12	0.02	0.03	0.05	13.91	3.80	1.87E-07	1.03E-04	-3.99	up	yes
ENSMUSG00000056553	Ptprn2	protein tyrosin	26.22	29.82	25.98	18.56	13.48	19.32	1.60	0.68	1.97E-07	1.07E-04	-3.97	up	no
ENSMUSG00000063681	Crb1	crumbs family m	0.18	0.14	0.12	0.03	0.04	0.05	3.79	1.92	2.64E-07	1.41E-04	-3.85	up	yes
ENSMUSG00000094293	Gm3893	predicted gene	1.43	2.46	1.01	0.22	0.34	0.27	5.85	2.55	4.13E-07	2.17E-04	-3.66	up	yes
ENSMUSG00000107927	Gm44090	predicted gene,	0	0.03	0.02	0.40	0.32	0.31	0.04	-4.56	4.39E-07	2.28E-04	-3.64	down	yes
ENSMUSG00000004798	Ulk2	unc-51 like kin	11.95	11.62	12.92	17.17	14.64	18.01	0.73	-0.45	5.48E-07	2.79E-04	-3.55	down	no
ENSMUSG00000074358	Ccdc61	coiled-coil dom	1.02	0.95	1.09	1.98	2.49	1.83	0.49	-1.04	5.77E-07	2.90E-04	-3.54	down	yes
ENSMUSG00000019478	Rab4a	RAB4A, member R	17.85	18.89	19.04	25.28	27.80	25.50	0.71	-0.49	7.58E-07	3.75E-04	-3.43	down	no
ENSMUSG00000098374	Gm28043	predicted gene,	4.26	2.73	3.01	1.88	1.13	1.88	2.04	1.03	9.62E-07	4.69E-04	-3.33	up	yes
ENSMUSG00000089862	Umad1	UMAP1-MVP12 ass	5.91	6.04	6.76	8.57	8.96	8.65	0.71	-0.48	1.24E-06	5.97E-04	-3.22	down	no
ENSMUSG00000090083	Rnf8	ring finger pro	1.20	1.32	1.48	2.03	1.89	1.96	0.68	-0.56	1.34E-06	6.33E-04	-3.20	down	no
ENSMUSG00000118603	BX666061	protein FAM205A	0.62	0.75	0.54	0.16	0.14	0.17	3.97	1.99	1.41E-06	6.57E-04	-3.18	up	yes
ENSMUSG00000019773	Fbxo5	F-box protein 5	1.63	2.06	1.27	0.46	0.60	0.69	2.84	1.50	1.51E-06	6.93E-04	-3.16	up	yes
ENSMUSG00000053898	Ech1	enoyl coenzyme	11.10	11.09	12.53	16.40	19.51	16.05	0.67	-0.58	1.75E-06	7.85E-04	-3.11	down	no
ENSMUSG00000103770	Pcdha9	protocadherin a	1.08	0.85	1.33	0.53	0.36	0.19	3.02	1.60	1.75E-06	7.85E-04	-3.11	up	yes
ENSMUSG00000079641	Rpl39	ribosomal prote	20.75	20.36	22.50	30.67	42.85	29.85	0.62	-0.70	1.90E-06	8.40E-04	-3.08	down	no
ENSMUSG00000027951	Adar	adenosine deami	21.42	23.84	20.88	15.51	12.16	16.65	1.49	0.58	2.10E-06	9.16E-04	-3.04	up	no
ENSMUSG00000078126	Rpl23a-ps3	ribosomal prote	21.54	20.96	26.92	34.45	41.57	36.96	0.61	-0.70	2.16E-06	9.29E-04	-3.03	down	no
ENSMUSG00000005716	Pvalb	parvalbumin [So	41.50	38.87	49.63	71.28	75.60	55.73	0.64	-0.64	2.35E-06	9.99E-04	-3.00	down	no
ENSMUSG00000109205	Gm44954	predicted gene	2.63	3.64	2.91	0.47	0.02	0.25	12.35	3.63	2.47E-06	1.04E-03	-2.98	up	yes
ENSMUSG00000039001	Rps21	ribosomal prote	83.99	78.78	93.82	117.8	170.56	122.4	0.62	-0.68	2.50E-06	1.04E-03	-2.98	down	no
ENSMUSG00000041377	Ninj2	ninjurin 2 [Sou	1.28	0.91	0.73	2.21	3.11	2.93	0.35	-1.50	2.63E-06	1.08E-03	-2.97	down	yes
ENSMUSG00000062995	Ica1	islet cell auto	10.11	10.18	11.20	14.26	15.37	13.92	0.72	-0.47	3.91E-06	1.58E-03	-2.80	down	no
ENSMUSG00000082962	Gm10601	predicted pseud	0.65	0.78	0.40	0.07	0.09	0.10	7.11	2.83	4.47E-06	1.77E-03	-2.75	up	yes
ENSMUSG00000090122	Kcne1l	potassium volta	2.22	1.77	2.26	3.67	3.70	3.90	0.55	-0.85	4.48E-06	1.77E-03	-2.75	down	no
ENSMUSG00000066838	Zfp772	zinc finger pro	2.78	2.76	2.66	4.74	3.47	4.72	0.63	-0.66	5.57E-06	2.17E-03	-2.66	down	no
ENSMUSG00000023805	Synj2	synaptojanin 2	9.27	11.00	9.71	7.54	5.41	6.91	1.51	0.59	6.16E-06	2.38E-03	-2.62	up	no
ENSMUSG00000040987	Mill2	MHC I like leuk	0.96	0.99	1.08	0.56	0.37	0.52	2.11	1.08	6.31E-06	2.40E-03	-2.62	up	yes

ENSMUSG00000046056	Sbsn	suprabasin [Sou	0.90	1.16	1.00	1.99	1.48	2.08	0.55	-0.86	6.57E-06	2.48E-03	-2.61	down	no
ENSMUSG00000078721	Fam205a1	family with seq	0.92	0.98	0.72	0.42	0.38	0.36	2.25	1.17	7.08E-06	2.64E-03	-2.58	up	yes
ENSMUSG00000082414	Gm13303	predicted gene	1.25	1.22	0.96	0.42	0.22	0.36	3.45	1.79	7.19E-06	2.65E-03	-2.58	up	yes
ENSMUSG00000042831	Alkbh6	alkB homolog 6	5.17	5.54	6.09	7.98	9.23	7.69	0.67	-0.57	8.05E-06	2.93E-03	-2.53	down	no
ENSMUSG00000024208	Uqcc2	ubiquinol-cytocl	29.87	27.80	30.72	39.93	58.10	41.88	0.63	-0.66	9.49E-06	3.42E-03	-2.47	down	no
ENSMUSG00000018659	Pnpo	pyridoxine 5'-pl	17.02	15.14	17.11	21.35	21.35	21.70	0.77	-0.39	1.09E-05	3.90E-03	-2.41	down	no
ENSMUSG00000029993	Nfu1	NFU1 iron-sulfu	5.46	5.42	6.07	7.60	8.43	7.49	0.72	-0.47	1.17E-05	4.10E-03	-2.39	down	no
ENSMUSG00000020396	Nefh	neurofilament, l	23.33	21.24	25.73	43.16	28.55	31.77	0.68	-0.56	1.18E-05	4.10E-03	-2.39	down	no
ENSMUSG00000001911	Nfix	nuclear factor	14.17	15.20	13.05	10.26	9.60	10.82	1.38	0.47	1.21E-05	4.17E-03	-2.38	up	no
ENSMUSG00000099041	Gm28035	predicted gene,	2.84	1.75	1.60	1.13	0.67	1.01	2.20	1.13	1.23E-05	4.20E-03	-2.38	up	yes
ENSMUSG00000061983	Rps12	ribosomal prote	27.81	25.33	30.20	40.56	43.81	34.37	0.70	-0.51	1.28E-05	4.32E-03	-2.36	down	no
ENSMUSG00000029994	Anxa4	annexin A4 [Sou	2.01	2.00	2.01	1.01	1.30	1.13	1.75	0.80	1.29E-05	4.32E-03	-2.36	up	no

Table S2D: Selection of genes robustly altered in PS/E2H vs PS/E3H mice. FPKM values of genes in individual mice (indicated in underscored numeral), along with fold change (fc) value, p value adjusted for false discovery rate of 0.05 (q value, qval) are presented. N=4 mice/group. PE2H, PS19 mice homozygous for APOE2; PE3H, PS19 mice homozygous for APOE3.

gene_id	gene_name	Description	FPKM. PE2H_1	FPKM. PE2H_2	FPKM. PE2H_3	FPKM. PE2H_4	FPKM. PE3H_1	FPKM. PE3H_2	FPKM. PE3H_3	FPKM. PE3H_4	fc	log2 (fc)	pval	qval	log10 (qval)	regulation	significant
ENSMUSG00000109209	Gm45104	predicted gene	0.14	0.04	0.08	0.09	1.92	2.52	2.62	1.66	0.04	-4.61	1.00E-63	3.37E-59	-58.47	down	yes
ENSMUSG00000098975	Gm27177	predicted gene	0.05	0.07	0.08	0.05	1.92	0.88	0.75	1.83	0.05	-4.43	5.06E-40	8.52E-36	-35.07	down	yes
ENSMUSG00000096596	Gm10591	predicted gene	0.12	0.06	0.09	0.08	3.54	1.99	5.03	6.67	0.02	-5.61	1.74E-31	1.96E-27	-26.71	down	yes
ENSMUSG00000073878	Gm13304	predicted gene	0.19	0.15	0.13	0.11	3.69	2.10	5.68	5.87	0.03	-4.90	1.14E-30	9.58E-27	-26.02	down	yes
ENSMUSG00000090338	Gm17081	predicted gene	0.06	0.09	0.09	0.06	0.95	0.94	1.74	1.34	0.06	-4.10	3.46E-29	2.33E-25	-24.63	down	yes
ENSMUSG00000093954	Gm16867	predicted gene	0.02	0.06	0.09	0.05	2.44	1.21	1.34	1.95	0.03	-4.97	4.09E-27	2.29E-23	-22.64	down	yes
ENSMUSG00000095675	Ccl21b	chemokine (C-C	0.11	0.15	0.06	0.11	3.04	1.53	6.16	6.29	0.02	-5.33	2.07E-26	9.97E-23	-22.00	down	yes
ENSMUSG00000118458	Gm10599	predicted pseu	0.09	0.12	0.11	0.09	0.85	0.87	1.90	1.50	0.08	-3.60	1.37E-22	5.76E-19	-18.24	down	yes
ENSMUSG00000094065	Ccl21d	chemokine (C-C	0.10	0.13	0.11	0.06	1.64	1.45	3.89	4.22	0.04	-4.80	2.15E-21	8.06E-18	-17.09	down	yes
ENSMUSG00000073879	Gm5859	predicted pseu	0.09	0.12	0.13	0.08	0.70	0.76	1.38	1.31	0.10	-3.33	2.23E-19	7.52E-16	-15.12	down	yes
ENSMUSG00000006154	Eps8l1	EPS8-like 1 [S	0.82	0.53	0.71	0.96	0.18	0.16	0.14	0.22	4.27	2.09	8.39E-17	2.57E-13	-12.59	up	yes
ENSMUSG00000091542	Gm17167	predicted gene	0.10	0.09	0.10	0.04	0.41	0.48	1.03	0.81	0.12	-3.05	1.01E-16	2.85E-13	-12.55	down	yes
ENSMUSG00000025453	Nnt	nicotinamide n	4.55	3.91	4.80	4.53	1.32	1.07	2.45	1.62	2.75	1.46	5.19E-15	1.34E-11	-10.87	up	yes
ENSMUSG00000095562	Erdr1	erythroid diff	10.90	7.65	7.90	11.85	4.23	3.34	3.00	3.84	2.66	1.41	1.68E-14	4.04E-11	-10.39	up	yes
ENSMUSG00000107705	Gm45062	predicted gene	0	0	0	0	0	0	0	5.76	0.00	-13.81	2.10E-14	4.71E-11	-10.33	down	yes
ENSMUSG00000054594	Oscar	osteoclast ass	0.57	0.52	0.62	0.85	0.06	0.03	0.05	0.07	11.95	3.58	4.20E-14	8.85E-11	-10.05	up	yes
ENSMUSG00000053985	Zfp14	zinc finger pr	2.79	2.63	2.68	2.74	4.28	5.29	6.22	4.66	0.53	-0.92	1.28E-12	2.54E-09	-8.60	down	no
ENSMUSG00000096862	Gm13301	predicted gene	0.05	0.04	0.05	0.03	0.52	0.27	0.53	0.61	0.09	-3.51	3.62E-12	6.78E-09	-8.17	down	yes
ENSMUSG00000096768	Gm47283	predicted gene	5.23	4.64	3.21	5.14	1.52	2.49	1.35	1.78	2.55	1.35	4.08E-12	7.24E-09	-8.14	up	yes
ENSMUSG00000068457	Uty	ubiquitously t	0.01	0.88	0	0	0	0	0	0	2207.08	11.11	4.44E-12	7.37E-09	-8.13	up	yes
ENSMUSG00000110105	Gm45844	predicted gene	0.48	0.43	0.50	0.47	0.05	0.06	0.18	0.08	5.21	2.38	4.60E-12	7.37E-09	-8.13	up	yes
ENSMUSG00000086604	Gm15510	predicted gene	1.23	1.25	0.50	1.75	0.15	0.13	0.12	0.11	9.40	3.23	1.17E-11	1.79E-08	-7.75	up	yes
ENSMUSG00000089865	Gm44503	predicted read	0	0	0	0	0	0	0	0.72	0.00	-10.81	2.23E-11	3.27E-08	-7.49	down	yes
ENSMUSG00000109176	Zfp264	zinc finger pr	0.05	0.02	0	0.02	0.37	0.41	0.55	0.27	0.06	-4.16	3.56E-11	4.99E-08	-7.30	down	yes
ENSMUSG00000109061	Gm49320	predicted gene	0	0	0	0	0.65	0	0	0	0.00	-10.67	8.19E-11	1.10E-07	-6.96	down	yes
ENSMUSG00000099876	Gm29650	predicted gene	0	3.61	0	0	0	0	0	0	9028.21	13.14	1.76E-10	2.28E-07	-6.64	up	yes
ENSMUSG00000076508	Igkv17-127	immunoglobulin	0	0	0	0	0	0	3.78	0	0.00	-13.21	2.43E-10	3.03E-07	-6.52	down	yes
ENSMUSG00000030413	Pglyrp1	peptidoglycan	2.49	2.78	2.34	5.70	0.63	0.93	1.18	1.06	3.51	1.81	4.83E-10	5.81E-07	-6.24	up	yes
ENSMUSG00000095753	Igkv4-53	immunoglobulin	0	0	0	0	0	2.45	0	0	0.00	-12.58	6.73E-10	7.81E-07	-6.11	down	yes
ENSMUSG00000090996	Gm20458	predicted gene	0	0	0	0.70	0	0	0	0	1746.25	10.77	1.83E-09	2.03E-06	-5.69	up	yes
ENSMUSG00000071470	Ccnb1ip1	cyclin B1 inte	0	0.51	0	0	0	0	0	0	1271.39	10.31	1.87E-09	2.03E-06	-5.69	up	yes
ENSMUSG00000030037	Mrpl53	mitochondrial	9.20	8.13	11.10	8.06	5.36	3.84	4.16	3.42	2.17	1.12	2.89E-09	3.04E-06	-5.52	up	yes
ENSMUSG00000072676	Tmem254a	transmembrane	1.76	2.48	1.74	1.97	4.05	4.85	3.59	3.71	0.49	-1.03	4.19E-09	4.27E-06	-5.37	down	yes
ENSMUSG00000053964	Lgals4	lectin, galact	0.78	0.83	0.85	1.24	0.22	0.33	0.26	0.34	3.18	1.67	1.03E-08	1.02E-05	-4.99	up	yes
ENSMUSG00000074358	Ccdc61	coiled-coil dc	2.29	1.89	2.01	1.99	1.10	0.71	1.18	0.95	2.07	1.05	3.46E-08	3.33E-05	-4.48	up	yes
ENSMUSG00000035299	Mid1	midline 1 [Sou	1.39	0.78	1.14	1.53	0.67	0.36	0.34	0.41	2.72	1.45	4.56E-08	4.27E-05	-4.37	up	yes
ENSMUSG00000084010	Gm13302	predicted gene	0.11	0.06	0.11	0.04	0.35	0.39	0.75	0.64	0.15	-2.74	7.42E-08	6.76E-05	-4.17	down	yes
ENSMUSG00000064179	Tnnt1	troponin T1, s	4.88	6.78	3.13	3.93	1.84	2.25	2.46	2.13	2.16	1.11	1.35E-07	1.20E-04	-3.92	up	yes
ENSMUSG00000039629	Strip2	striatin inter	5.19	4.90	5.12	4.29	6.32	8.80	7.18	7.39	0.66	-0.61	1.40E-07	1.21E-04	-3.92	down	no
ENSMUSG00000109205	Gm44954	predicted gene	0.29	0.53	0.78	0.45	3.27	4.63	2.65	3.01	0.15	-2.72	1.63E-07	1.37E-04	-3.86	down	yes
ENSMUSG00000074361	C5ar2	complement com	0.15	0.20	0.30	0.21	0.05	0.03	0.03	0.01	7.00	2.81	2.31E-07	1.90E-04	-3.72	up	yes
ENSMUSG00000093909	Gm3883	predicted gene	0.91	0.79	1.16	0.70	1.61	1.58	2.24	1.97	0.48	-1.06	2.57E-07	2.06E-04	-3.69	down	yes
ENSMUSG00000095463	Entpd4	ectonucleoside	4.12	4.57	3.83	2.77	7.48	6.19	5.76	7.76	0.56	-0.83	3.65E-07	2.86E-04	-3.54	down	no
ENSMUSG00000045948	Mrps12	mitochondrial	21.25	24.31	17.12	18.51	14.34	11.94	11.86	10.87	1.66	0.73	4.00E-07	3.06E-04	-3.51	up	no
ENSMUSG00000058626	Capn11	calpain 11 [Sc	0.13	0.06	0.10	0.06	0.08	1.17	0.46	1.61	0.11	-3.25	4.59E-07	3.44E-04	-3.46	down	yes
ENSMUSG00000096256	Gm21093	predicted gene	1.04	0.89	1.21	1.02	1.82	1.64	2.07	2.26	0.54	-0.90	4.83E-07	3.54E-04	-3.45	down	no
ENSMUSG00000118425	Gm50470	predicted gene	0.16	0.11	0.15	0.14	0.38	0.46	0.88	0.72	0.22	-2.17	5.22E-07	3.74E-04	-3.43	down	yes
ENSMUSG00000035429	Ptprh	protein tyrosi	0.31	0.30	0.29	0.39	0	0.06	0.07	0.04	7.39	2.88	5.37E-07	3.77E-04	-3.42	up	yes
ENSMUSG00000035692	Isg15	ISG15 ubiquiti	10.13	10.17	7.63	8.89	2.77	3.55	3.66	6.50	2.23	1.16	1.42E-06	9.73E-04	-3.01	up	yes
ENSMUSG00000040569	Slc26a7	solute carrier	0.49	0.47	0.81	0.35	0.83	1.45	1.20	1.56	0.42	-1.25	1.93E-06	1.30E-03	-2.89	down	yes
ENSMUSG00000053898	Ech1	enoyl coenzyme	18.75	20.25	15.72	18.24	10.94	13.03	11.64	14.48	1.46	0.54	2.57E-06	1.70E-03	-2.77	up	no
ENSMUSG00000070469	Adamtsl3	ADAMTS-like 3	0.29	0.20	0.38	0.18	0.42	0.71	0.62	0.90	0.39	-1.34	2.95E-06	1.91E-03	-2.72	down	yes
ENSMUSG00000108366	Gm5586	predicted gene	9.86	0.34	0.36	10.58	0.15	0.15	0.17	0.11	36.49	5.19	3.80E-06	2.42E-03	-2.62	up	yes
ENSMUSG00000089998	Phtf1os	putative homec	2.72	2.08	0.66	1.19	0.65	0.35	0.72	0.52	2.97	1.57	5.29E-06	3.30E-03	-2.48	up	yes
ENSMUSG00000118434	Gm13301	predicted gene	0.17	0.13	0.34	0.26	0.99	0.63	1.40	1.26	0.21	-2.26	6.09E-06	3.73E-03	-2.43	down	yes
ENSMUSG00000044042	Fmn1	formin 1 [Sour	0.73	0.62	0.88	0.89	1.22	1.16	1.24	1.16	0.65	-0.62	6.88E-06	4.14E-03	-2.38	down	no
ENSMUSG00000033061	Resp18	regulated endc	89.72	87.99	88.71	95.57	59.37	79.64	73.60	64.15	1.31	0.39	7.19E-06	4.25E-03	-2.37	up	no
ENSMUSG00000030306	Tmtc1	transmembrane	7.38	6.87	8.09	6.95	9.38	9.87	10.32	8.91	0.76	-0.39	8.49E-06	4.93E-03	-2.31	down	no
ENSMUSG00000095348	Gm3892	predicted gene	0.28	0.15	0.28	0.15	0.47	0.51	0.70	0.67	0.37	-1.44	1.01E-05	5.74E-03	-2.24	down	yes
ENSMUSG00000096257	Ccer2	coiled-coil gl	1.96	2.48	2.13	2.03	4.15	3.25	5.05	3.80	0.53	-0.92	1.17E-05	6.56E-03	-2.18	down	no
ENSMUSG00000008435	Rdh13	retinol dehydr	3.00	3.07	2.54	2.83	4.17	3.76	4.62	3.94	0.69	-0.53	1.24E-05	6.82E-03	-2.17	down	no
ENSMUSG00000029561	Oasl2	2'-5' oligoade	3.70	2.32	2.60	4.05	0.95	1.67	1.95	2.09	1.90	0.93	1.28E-05	6.91E-03	-2.16	up	no
ENSMUSG00000073647	Gm10557	predicted gene	2.89	3.08	3.96	1.91	5.76	8.09	5.97	5.50	0.47	-1.10	1.29E-05	6.91E-03	-2.16	down	yes
ENSMUSG00000005575	Ube2m	ubiquitin-conj	48.12	57.90	42.84	50.10	38.40	33.83	34.38	37.25	1.38	0.47	1.41E-05	7.41E-03	-2.13	up	no
ENSMUSG00000034218	Atm	ataxia telangi	0.49	0.36	0.69	0.48	0.74	0.96	0.99	0.89	0.56	-0.83	1.60E-05	8.31E-03	-2.08	down	no
ENSMUSG00000064215	Ifi27	interferon, al	36.74	35.03	30.49	31.19	20.71	23.90	26.29	27.30	1.36	0.44	1.70E-05	8.69E-03	-2.06	up	no
ENSMUSG00000038775	Vill	villin-like [S	1.00	1.03	1.28	1.41	0.72	0.61	0.48	0.74	1.85	0.88	1.73E-05	8.72E-03	-2.06	up	no
ENSMUSG00000031626	Sorbs2	sorbin and SH3	2.58	2.49	2.97	2.63	3.46	3.35	3.92	3.31	0.76	-0.40	2.09E-05	1.04E-02	-1.98	down	no
ENSMUSG00000049148	Plcxd3	phosphatidylin	0.10	0.10	0.27	0.22	0.29	0.67	0.60	0.54	0.33	-1.60	2.21E-05	1.08E-02	-1.97	down	yes
ENSMUSG00000034320	Slc26a2	solute carrier	0.64	0.58	0.73	0.81	0.90	1.04	1.17	1.19	0.64	-0.64	2.33E-05	1.12E-02	-1.95	down	no
ENSMUSG00000091811	Inafm1	InaF motif con	15.83	19.28	15.41	15.33	11.60	12.87	10.04	12.04	1.41	0.50	2.40E-05	1.14E-02	-1.94	up	no
ENSMUSG00000066838	Zfp772	zinc finger pr	3.64	3.62	4.09	3.62	2.59	2.10	3.00	2.51	1.47	0.55	2.45E-05	1.15E-02	-1.94	up	no
ENSMUSG00000035674	Ndufa3	NADH:ubiquinon	32.63	37.53	25.93	31.12	21.22	23.29	19.35	23.89	1.45	0.54	2.54E-05	1.16E-02	-1.94	up	no
ENSMUSG00000035596	Mboat7	membrane bound	15.77	15.89	16.81	16.48	13.61	13.99	12.89	12.66	1.22	0.29	2.55E-05	1.16E-02	-1.94	up	no
ENSMUSG00000103640	Gm31406	predicted gene	0	0	0	0	0.26	0.32	0.13	0.40	0.00	-11.44	2.70E-05	1.21E-02	-1.92	down	yes
ENSMUSG00000116165	Pdxp	pyridoxal (pyr	63.42	72.83	59.70	69.80	37.51	49.04	33.14	56.19	1.51	0.60	3.41E-05	1.51E-02	-1.82	up	no
ENSMUSG00000061816	Myl1	myosin, light	0.10	0.09	0.21	0.05	0.52	0.59	0.19	1.08	0.19	-2.39	3.49E-05	1.53E-02	-1.82	down	yes
ENSMUSG00000002012	Pnck	pregnancy upre	36.88	45.12	36.64	40.61	30.78	28.63	32.08	30.90	1.30	0.38	3.67E-05	1.58E-02	-1.80	up	no
ENSMUSG00000061119	Prcp	prolylcarboxyp	1.58	1.78	2.05	1.63	2.21	2.68	2.66	2.60	0.69	-0.53	3.96E-05	1.67E-02	-1.78	down	no
ENSMUSG00000104597	B230334C09Ri	RIKEN cDNA B23	6.97	6.22	7.47	5.66	8.80	8.93	11.68	8.27	0.70	-0.52	3.98E-05	1.67E-02	-1.78	down	no
ENSMUSG00000091177	Gm15494	predicted gene	0.05	0	0.11	0.42	0.86	1.18	1.92	1.24	0.11	-3.15	4.23E-05	1.75E-02	-1.76	down	yes
ENSMUSG00000095304	Plac9a	placenta speci	0.72	0.39	0.38	0.36	1.18	1.78	1.22	0.81	0.37	-1.43	4.26E-05	1.75E-02	-1.76	down	yes
ENSMUSG00000022829	Stxbp5l	syntaxin bindi	0.74	0.65	1.17	0.93	1.23	1.52	1.59	1.44	0.60	-0.73	4.58E-05	1.85E-02	-1.73	down	no
ENSMUSG00000090553	Snrpe	small nuclear	10.98	10.77	11.68	10.42	8.52	8.72	7.14	7.66	1.37	0.45	4.62E-05	1.85E-02	-1.73	up	no
ENSMUSG00000040987	Mill2	MHC I like leu	0.52	0.60	0.63	0.36	0.76	1.17	1.09	0.97	0.53	-0.92	4.67E-05	1.85E-02	-1.73	down	no
ENSMUSG00000039556	Ppp1r3f	protein phosph	4.63	4.82	4.44	5.85	3.03	3.58	2.90	4.03	1.46	0.54	4.75E-05	1.85E-02	-1.73	up	no
ENSMUSG00000043635	Adamts3	a disintegrin-	0.84	0.62	1.08	0.74	1.33	1.22	1.56	1.46	0.59	-0.77	4.79E-05	1.85E-02	-1.73	down	no
ENSMUSG00000027858	Tspan2	tetraspanin 2	22.41	21.15	26.38	18.30	26.37	32.10	32.31	31.12	0.72	-0.47	5.18E-05	1.98E-02	-1.70	down	no
ENSMUSG00000072680	Tmem254c	transmembrane	5.72	5.65	4.22	5.90	9.39	7.45	7.86	10.51	0.61	-0.71	5.24E-05	1.98E-02	-1.70	down	no
ENSMUSG00000022066	Entpd4b	ectonucleoside	5.08	5.35	5.21	5.57	8.88	6.72	6.65	8.71	0.69	-0.55	5.36E-05	2.01E-02	-1.70	down	no
ENSMUSG00000055296	Tmem245	transmembrane	2.57	2.61	3.32	2.71	3.56	3.77	4.10	3.54	0.75	-0.42	5.60E-05	2.07E-02	-1.68	down	no
ENSMUSG00000048347	Pcdhb18	protocadherin	0.75	0.85	0.67	0.62	1.04	1.24	1.57	1.07	0.59	-0.77	5.64E-05	2.07E-02	-1.68	down	no
ENSMUSG00000026675	Hsd17b7	hydroxysteroid	2.83	2.40	3.47	2.44	4.37	4.32	4.27	3.59	0.67	-0.57	5.81E-05	2.11E-02	-1.68	down	no
ENSMUSG00000090667	Gm765	predicted gene	1.22	0.47	1.51	0.54	3.22	1.66	2.03	2.36	0.40	-1.31	6.66E-05	2.39E-02	-1.62	down	yes
ENSMUSG00000093996	Fam205a3	family with se	0.71	0.73	0.86	0.53	1.09	1.14	1.39	1.22	0.59	-0.77	7.28E-05	2.58E-02	-1.59	down	no
ENSMUSG00000098900	Gm18190	predicted gene	0.28	0.14	0.19	0.15	0	0.02	0.02	0.02	13.38	3.74	7.59E-05	2.66E-02	-1.57	up	yes
ENSMUSG00000063623	C230062I16Ri	RIKEN cDNA C23	0.38	0.47	0.31	0.53	0.26	0.12	0.19	0.19	2.23	1.15	7.77E-05	2.70E-02	-1.57	up	yes
ENSMUSG00000078746	Fam205a4	family with se	0.68	0.69	0.83	0.51	1.08	1.09	1.35	1.14	0.58	-0.77	7.96E-05	2.73E-02	-1.56	down	no
ENSMUSG00000059751	Rps3a3	ribosomal prot	61.02	2.45	1.77	60.21	1.43	1.33	1.51	2.19	19.43	4.28	8.08E-05	2.75E-02	-1.56	up	yes

Table S2E: Selection of genes altered in PS/E4H vs PS/E3H mice. FPKM values of genes in individual mice (indicated in underscored numeral), along with fold change (fc) value, p value adjusted for false discovery rate of 0.05 (q value, qval) are presented. N=4 mice/group. PE4H, PS19 mice homozygous for APOE4; PE3H, PS19 mice homozygous for APOE3.

gene_id	gene_name	Description	FPKM. PE4H_1	FPKM. PE4H_2	FPKM. PE4H_3	FPKM. PE4H_4	FPKM. PE3H_1	FPKM. PE3H_2	FPKM. PE3H_3	FPKM. PE3H_4	fc	log2 (fc)	pval	qval	log10 (qval)	regulation	significant
ENSMUSG00000086604	Gm15510	predicted gene	1.31	1.41	0.88	1.41	0.15	0.13	0.12	0.11	9.93	3.31	1.28E-16	4.33E-12	-11.36	up	yes
ENSMUSG00000054594	Oscar	osteoclast ass	0.68	0.52	0.53	0.68	0.06	0.03	0.05	0.07	11.24	3.49	6.51E-14	7.84E-10	-9.11	up	yes
ENSMUSG00000006154	Eps8l1	EPS8-like 1 [S	0.45	0.81	0.66	0.91	0.18	0.16	0.14	0.22	4.00	2.00	6.99E-14	7.84E-10	-9.11	up	yes
ENSMUSG00000031562	Dctd	dCMP deaminase	0.90	0.79	0.86	0.90	1.58	2.05	1.77	1.91	0.47	-1.08	9.35E-13	7.87E-09	-8.10	down	yes
ENSMUSG00000089865	Gm44503	predicted read	0	0	0	0	0	0	0	0.72	0.00	-10.81	2.26E-11	1.52E-07	-6.82	down	yes
ENSMUSG00000021342	Prl	prolactin [Sou	0	1.59	0	0	0	0	0	0	3979.5	11.96	1.95E-10	1.09E-06	-5.96	up	yes
ENSMUSG00000076508	Igkv17-127	immunoglobulin	0	0	0	0	0	0	3.78	0	0.00	-13.21	2.43E-10	1.17E-06	-5.93	down	yes
ENSMUSG00000078519	2310026L22Rik	RIKEN cDNA 231	0	0	0	0	0	2.42	0	0	0.00	-12.56	5.51E-10	2.32E-06	-5.63	down	yes
ENSMUSG00000095753	Igkv4-53	immunoglobulin	0	0	0	0	0	2.45	0	0	0.00	-12.58	6.70E-10	2.51E-06	-5.60	down	yes
ENSMUSG00000095007	Igkv12-41	immunoglobulin	0	3.48	0	0	0	0	0	0	8709.1	13.09	1.05E-09	3.52E-06	-5.45	up	yes
ENSMUSG00000092232	Gm20521	predicted gene	0	2.22	0	0	0	0	0	0	5553.9	12.44	2.00E-09	6.11E-06	-5.21	up	yes
ENSMUSG00000087467	Gm13601	predicted gene	0	0	0	0.31	2.11	2.38	1.88	1.99	0.04	-4.75	2.53E-08	7.11E-05	-4.15	down	yes
ENSMUSG00000069045	Ddx3y	DEAD (Asp-Glu-	10.64	0.04	0.06	11.96	0.02	0.02	0.02	0.01	334.97	8.39	3.01E-08	7.80E-05	-4.11	up	yes
ENSMUSG00000110899	Gm48840	predicted gene	0.02	0.02	0.35	0.10	0	0	0	0	1258.5	10.30	7.83E-08	1.87E-04	-3.73	up	yes
ENSMUSG00000001119	Col6a1	collagen, type	5.05	5.21	3.86	6.02	6.49	8.54	9.44	8.51	0.61	-0.71	8.34E-08	1.87E-04	-3.73	down	no
ENSMUSG00000091177	Gm15494	predicted gene	0	0.21	0.06	0.14	0.86	1.18	1.92	1.24	0.08	-3.64	1.22E-07	2.56E-04	-3.59	down	yes
ENSMUSG00000074280	Gm6166	predicted gene	1.28	29.43	22.90	28.61	1.18	1.07	0.61	1.29	19.80	4.31	1.93E-07	3.82E-04	-3.42	up	yes
ENSMUSG00000004798	Ulk2	unc-51 like ki	15.01	13.16	11.21	12.31	16.91	17.27	18.87	18.77	0.72	-0.47	3.80E-07	7.11E-04	-3.15	down	no
ENSMUSG00000062132	Arhgap33os	Rho GTPase act	0.17	0.27	0.11	0.16	0	0.02	0	0	40.90	5.35	5.06E-07	8.97E-04	-3.05	up	yes
ENSMUSG00000027375	Mal	myelin and lym	105.1	104.3	101.2	110.44	83.77	84.12	93.85	83.29	1.22	0.29	7.30E-07	1.23E-03	-2.91	up	no
ENSMUSG00000064179	Tnnt1	troponin T1, s	5.29	3.60	3.79	3.11	1.84	2.25	2.46	2.13	1.82	0.86	1.21E-06	1.94E-03	-2.71	up	no
ENSMUSG00000068457	Uty	ubiquitously t	1.24	0.00	0.01	1.22	0	0	0	0	6203.3	12.60	2.80E-06	4.28E-03	-2.37	up	yes
ENSMUSG00000008435	Rdh13	retinol dehydr	3.05	3.18	2.42	2.80	4.17	3.76	4.62	3.94	0.69	-0.53	3.30E-06	4.83E-03	-2.32	down	no
ENSMUSG00000058740	Kcnt1	potassium chan	7.92	6.97	7.29	7.90	8.88	9.94	10.45	8.83	0.79	-0.34	3.81E-06	5.22E-03	-2.28	down	no
ENSMUSG00000030123	Plxnd1	plexin D1 [Sou	5.94	4.89	5.98	4.73	8.10	8.12	6.89	8.01	0.69	-0.53	3.88E-06	5.22E-03	-2.28	down	no
ENSMUSG00000035429	Ptprh	protein tyrosi	0.24	0.30	0.27	0.38	0	0.06	0.07	0.04	6.80	2.76	4.09E-06	5.30E-03	-2.28	up	yes
ENSMUSG00000081406	Rps6-ps4	ribosomal prot	35.19	37.65	34.82	37.59	25.14	28.87	26.80	29.73	1.31	0.39	5.29E-06	6.60E-03	-2.18	up	no
ENSMUSG00000046056	Sbsn	suprabasin [So	1.21	0.72	0.90	1.13	1.40	2.83	2.03	1.70	0.50	-1.01	7.18E-06	8.63E-03	-2.06	down	yes
ENSMUSG00000069049	Eif2s3y	eukaryotic tra	8.41	0.04	0.01	8.60	0.01	0	0.01	0	774.18	9.60	8.28E-06	9.62E-03	-2.02	up	yes
ENSMUSG00000022565	Plec	plectin [Sourc	4.36	3.56	3.72	3.65	5.10	4.88	4.92	4.99	0.77	-0.38	9.55E-06	1.04E-02	-1.98	down	no
ENSMUSG00000043773	1700048O20Rik	RIKEN cDNA 170	1.91	3.09	2.73	2.73	4.74	3.67	4.78	4.98	0.58	-0.80	9.57E-06	1.04E-02	-1.98	down	no
ENSMUSG00000072676	Tmem254a	transmembrane	1.84	1.64	2.92	2.59	4.05	4.85	3.59	3.71	0.56	-0.85	1.24E-05	1.31E-02	-1.88	down	no
ENSMUSG00000108282	Gm44317	predicted gene	0	0	0	0	0.02	0.06	0.08	0.04	0.00	-8.92	1.36E-05	1.39E-02	-1.86	down	yes
ENSMUSG00000057021	Vmn2r-ps159	vomeronasal 2,	0	0.15	0.09	0.16	0	0	0	0	990.18	9.95	1.40E-05	1.39E-02	-1.86	up	yes
ENSMUSG00000072680	Tmem254c	transmembrane	5.17	3.91	4.91	6.88	9.39	7.45	7.86	10.51	0.59	-0.75	1.55E-05	1.49E-02	-1.83	down	no
ENSMUSG00000053141	Ptprt	protein tyrosi	7.42	6.45	6.65	7.08	8.62	8.56	9.48	7.93	0.80	-0.33	1.61E-05	1.51E-02	-1.82	down	no
ENSMUSG00000032517	Mobp	myelin-associa	192.2	211.8	192.8	165.38	141.83	155.60	151.91	142.76	1.29	0.36	2.57E-05	2.34E-02	-1.63	up	no
ENSMUSG00000020102	Slc16a7	solute carrier	0.39	0.41	0.39	0.41	2.41	2.30	0.46	1.59	0.24	-2.08	2.74E-05	2.43E-02	-1.61	down	yes
ENSMUSG00000100690	Gm4322	predicted gene	2.77	1.88	0	2.84	0.09	0	0.11	0	37.35	5.22	3.07E-05	2.65E-02	-1.58	up	yes
ENSMUSG00000032128	Robo3	roundabout gui	0.51	0.38	0.67	0.53	0.98	0.93	1.10	0.73	0.56	-0.84	3.87E-05	3.26E-02	-1.49	down	no
ENSMUSG00000052353	Cemip	cell migration	0.93	0.69	0.72	0.60	1.21	0.98	1.21	1.15	0.65	-0.63	4.02E-05	3.30E-02	-1.48	down	no
ENSMUSG00000067916	Zfp991	zinc finger pr	1.44	0.83	0.82	0.96	1.45	1.92	2.14	1.89	0.55	-0.87	4.52E-05	3.63E-02	-1.44	down	no
ENSMUSG00000019577	Pdk4	pyruvate dehyd	5.03	5.00	6.17	5.73	3.50	3.01	4.66	3.78	1.47	0.55	4.68E-05	3.66E-02	-1.44	up	no
ENSMUSG00000044783	Hjurp	Holliday junct	5.01	4.11	4.29	5.45	7.09	8.72	5.31	6.71	0.68	-0.56	5.55E-05	4.25E-02	-1.37	down	no
ENSMUSG00000026463	Atp2b4	ATPase, Ca++ t	11.44	10.29	9.71	10.08	14.60	12.82	13.39	12.35	0.78	-0.36	6.01E-05	4.50E-02	-1.35	down	no
ENSMUSG00000087263	Gm15726	predicted gene	0.04	0.03	0.05	0	0.76	0.52	0.42	0.55	0.05	-4.24	6.78E-05	4.97E-02	-1.30	down	yes
ENSMUSG00000105096	Gbp10	guanylate-bind	0.37	0.26	0.30	0.18	0.04	0.05	0.07	0.14	3.67	1.88	7.25E-05	5.20E-02	-1.28	up	no
ENSMUSG00000029695	Aass	aminoadipate-s	1.96	1.78	2.21	1.83	1.22	1.37	1.43	1.37	1.44	0.53	7.76E-05	5.42E-02	-1.27	up	no
ENSMUSG00000056673	Kdm5d	lysine (K)-spe	1.42	0.00	0	1.44	0.00	0.00	0	0.00	407.50	8.67	7.88E-05	5.42E-02	-1.27	up	no
ENSMUSG00000107478	Gm45234	predicted gene	0	0	0	0	0.02	0.10	0.01	0.05	0.00	-8.78	8.61E-05	5.78E-02	-1.24	down	no
ENSMUSG00000085813	Gm15870	predicted gene	0	0	0	0	0.34	0.19	0.41	0.09	0.00	-11.31	8.75E-05	5.78E-02	-1.24	down	no
ENSMUSG00000045482	Trrap	transformation	3.07	2.54	2.90	2.55	3.36	3.53	3.75	3.39	0.79	-0.34	9.15E-05	5.92E-02	-1.23	down	no
ENSMUSG00000102742	Pcdhga11	protocadherin	6.70	7.53	8.16	6.57	9.82	9.76	9.02	10.21	0.75	-0.42	9.76E-05	6.20E-02	-1.21	down	no
ENSMUSG00000084350	Znf41-ps	ZNF41, pseudog	1.12	0.80	0.46	0.89	1.44	1.38	2.35	1.59	0.48	-1.05	1.17E-04	7.30E-02	-1.14	down	no
ENSMUSG00000090222	Ifi203-ps	interferon act	0	0	0	0	0.02	0.07	0.07	0.07	0.00	-9.23	1.28E-04	7.84E-02	-1.11	down	no
ENSMUSG00000110631	Gm42047	predicted gene	0.23	0.23	0.20	0.71	0.65	1.56	2.17	1.25	0.24	-2.03	1.36E-04	8.21E-02	-1.09	down	no
ENSMUSG00000068740	Celsr2	cadherin, EGF	9.10	7.11	7.99	7.32	10.68	10.97	9.49	9.84	0.77	-0.38	1.43E-04	8.32E-02	-1.08	down	no
ENSMUSG00000073643	Wdfy1	WD repeat and	2.53	4.70	5.28	2.45	7.18	8.85	9.54	8.30	0.44	-1.18	1.43E-04	8.32E-02	-1.08	down	no
ENSMUSG00000018707	Dync1h1	dynein cytopla	24.41	17.68	19.75	18.42	25.65	26.83	26.14	25.21	0.77	-0.37	1.52E-04	8.66E-02	-1.06	down	no
ENSMUSG00000110386	Gm42031	predicted gene	0.09	0.04	0.22	0.06	0.07	0.85	0.70	0.93	0.16	-2.64	1.58E-04	8.85E-02	-1.05	down	no
ENSMUSG00000041936	Agrn	agrin [Source:	9.49	7.30	8.02	7.50	10.36	10.78	10.36	9.81	0.78	-0.35	1.64E-04	8.93E-02	-1.05	down	no
ENSMUSG00000074065	Gm10617	predicted gene	0	0	0	0	0.15	0.11	0.09	0.13	0.00	-10.22	1.64E-04	8.93E-02	-1.05	down	no
ENSMUSG00000066553	Gm6969	predicted pseu	56.61	63.96	1.74	1.55	1.37	1.87	1.39	1.70	19.57	4.29	1.72E-04	9.08E-02	-1.04	up	no
ENSMUSG00000021223	Papln	papilin, prote	0.11	0.14	0.13	0.17	0.23	0.27	0.42	0.29	0.45	-1.14	1.73E-04	9.08E-02	-1.04	down	no
ENSMUSG00000041453	Rpl21	ribosomal prot	12.84	14.47	15.09	11.16	8.44	7.69	11.39	10.24	1.42	0.50	1.75E-04	9.08E-02	-1.04	up	no
ENSMUSG00000103144	Pcdhga1	protocadherin	0.66	0.71	0.48	0.54	2.11	0.77	1.40	0.83	0.47	-1.09	1.89E-04	9.54E-02	-1.02	down	no
ENSMUSG00000114496	Gm47601	predicted gene	0	0	0	0	0.07	0.04	0.16	0.02	0.00	-9.50	1.90E-04	9.54E-02	-1.02	down	no
ENSMUSG00000107927	Gm44090	predicted gene	0	0.02	0.03	0	0.29	0.09	0.21	0.13	0.07	-3.90	2.12E-04	1.04E-01	-0.98	down	no
ENSMUSG00000074634	Tmem267	transmembrane	2.79	4.14	5.62	2.75	1.65	2.41	2.78	2.03	1.72	0.79	2.13E-04	1.04E-01	-0.98	up	no
ENSMUSG00000030554	Synm	synemin, inter	4.74	4.75	4.53	4.37	6.02	5.23	5.88	6.30	0.78	-0.35	2.25E-04	1.07E-01	-0.97	down	no
ENSMUSG00000041757	Plekha6	pleckstrin hom	6.68	5.37	5.35	5.84	6.92	7.60	7.81	6.68	0.80	-0.32	2.27E-04	1.07E-01	-0.97	down	no
ENSMUSG00000020241	Col6a2	collagen, type	1.61	1.70	0.87	1.47	2.15	2.01	2.19	2.53	0.64	-0.65	2.29E-04	1.07E-01	-0.97	down	no
ENSMUSG00000020673	Tpo	thyroid peroxi	0.02	0.05	0.02	0.04	0.12	0.25	0.19	0.07	0.21	-2.23	2.40E-04	1.10E-01	-0.96	down	no
ENSMUSG00000072844	G530011O06Rik	RIKEN cDNA G53	0.08	0.36	0.42	0.08	0.86	0.68	0.89	0.87	0.29	-1.81	2.41E-04	1.10E-01	-0.96	down	no
ENSMUSG00000068396	Rpl34-ps1	ribosomal prot	91.15	125.0	97.30	100.48	67.64	86.69	73.71	79.32	1.35	0.43	2.53E-04	1.14E-01	-0.94	up	no
ENSMUSG00000046688	Tifa	TRAF-interacti	5.52	4.62	4.65	4.93	2.46	4.59	3.02	3.11	1.50	0.58	2.64E-04	1.17E-01	-0.93	up	no
ENSMUSG00000032855	Pkd1	polycystin 1,	3.71	2.66	2.67	2.84	3.55	4.05	4.43	3.71	0.75	-0.41	2.83E-04	1.23E-01	-0.91	down	no
ENSMUSG00000078862	Gm14326	predicted gene	2.18	5.16	4.01	2.34	5.57	6.15	6.28	6.73	0.55	-0.85	2.85E-04	1.23E-01	-0.91	down	no
ENSMUSG00000049630	C1ql3	C1q-like 3 [So	9.95	9.34	9.61	8.53	15.20	11.99	12.59	11.16	0.74	-0.44	3.16E-04	1.34E-01	-0.87	down	no
ENSMUSG00000095304	Plac9a	placenta speci	0.32	0.82	0.47	0.26	1.18	1.78	1.22	0.81	0.38	-1.41	3.19E-04	1.34E-01	-0.87	down	no
ENSMUSG00000094546	Ighv1-26	immunoglobulin	0	0	0	0	0	0.24	0.27	2.09	0.00	-12.67	3.42E-04	1.42E-01	-0.85	down	no
ENSMUSG00000031737	Irx5	Iroquois homeo	0.13	0.17	0.09	0.08	0.23	0.38	0.34	0.33	0.36	-1.47	3.46E-04	1.42E-01	-0.85	down	no
ENSMUSG00000095366	Gm21860	predicted gene	0.24	0.13	0.27	0.61	0.79	0.99	0.94	1.14	0.32	-1.63	3.74E-04	1.52E-01	-0.82	down	no
ENSMUSG00000038156	Spon1	spondin 1, (f-	7.01	6.98	7.22	6.84	8.48	9.45	7.80	8.94	0.81	-0.31	3.87E-04	1.55E-01	-0.81	down	no
ENSMUSG00000021182	Ccdc88c	coiled-coil do	1.53	1.47	1.57	1.64	1.74	2.04	1.92	2.13	0.79	-0.33	4.16E-04	1.65E-01	-0.78	down	no
ENSMUSG00000057230	Aak1	AP2 associated	13.28	11.00	12.78	12.95	15.54	14.67	17.29	14.09	0.81	-0.30	4.24E-04	1.66E-01	-0.78	down	no
ENSMUSG00000004113	Cacna1b	calcium channe	5.96	4.89	5.69	5.65	6.85	7.32	7.03	6.21	0.81	-0.30	4.29E-04	1.66E-01	-0.78	down	no
ENSMUSG00000089694	Nat8f7	N-acetyltransf	0.93	0.74	0.91	1.28	0.49	0.53	0.54	0.46	1.91	0.93	4.38E-04	1.67E-01	-0.78	up	no
ENSMUSG00000046952	Gm5815	predicted pseu	0.97	0.49	0.94	0.64	0.10	0.12	0.31	0.37	3.41	1.77	4.41E-04	1.67E-01	-0.78	up	no
ENSMUSG00000035722	Abca7	ATP-binding ca	1.78	1.72	1.75	1.59	2.12	2.64	2.22	2.07	0.76	-0.41	4.50E-04	1.68E-01	-0.77	down	no
ENSMUSG00000076501	Igkv2-137	immunoglobulin	0	0	0	0	0	0.06	0.34	1.43	0.00	-12.15	4.55E-04	1.68E-01	-0.77	down	no
ENSMUSG00000105558	Gm5855	predicted gene	0.24	1.06	0.68	1.46	0.17	0.13	0.43	0.08	4.28	2.10	4.63E-04	1.68E-01	-0.77	up	no
ENSMUSG00000092375	A730060N03Rik	RIKEN cDNA A73	0.38	0.15	0.40	0.16	0.65	0.63	1.20	0.55	0.35	-1.50	4.69E-04	1.68E-01	-0.77	down	no
ENSMUSG00000034390	Cmip	c-Maf inducing	11.34	10.00	10.95	9.38	13.38	13.86	12.39	12.63	0.80	-0.33	4.70E-04	1.68E-01	-0.77	down	no
ENSMUSG00000105769	Gm43351	predicted gene	0	0	0	0	0.38	0.15	0.59	0.41	0.00	-11.91	4.77E-04	1.69E-01	-0.77	down	no
ENSMUSG00000107470	Gm3375	predicted gene	27.42	29.17	2.20	2.67	2.06	1.55	1.42	2.24	8.44	3.08	4.86E-04	1.70E-01	-0.77	up	no
ENSMUSG00000050103	Agmo	alkylglycerol	1.61	1.55	1.41	1.45	1.04	0.92	1.30	1.12	1.38	0.46	4.98E-04	1.72E-01	-0.76	up	no
ENSMUSG00000029716	Tfr2	transferrin re	0.77	0.66	0.58	0.63	0.94	1.24	0.97	0.93	0.65	-0.63	5.00E-04	1.72E-01	-0.76	down	no
ENSMUSG00000064193	Gm4735	predicted gene	30.83	87.21	84.35	93.26	33.78	31.69	29.65	32.56	2.32	1.21	5.13E-04	1.73E-01	-0.76	up	no

Table S2F: Selection of genes altered in PS/E4H vs PS/E2H mice. FPKM values of genes in individual mice (indicated in underscored numeral), along with fold change (fc) value, p value adjusted for false discovery rate of 0.05 (q value, qval) are presented. N=4 mice/group. PE4H, PS19 mice homozygous for APOE4; PE2H, PS19 mice homozygous for APOE2.

gene_id	gene_name	Description	FPKM. PE4H_1	FPKM. PE4H_2	FPKM. PE4H_3	FPKM. PE4H_4	FPKM. PE2H_1	FPKM. PE2H_2	FPKM. PE2H_3	FPKM.PE 2H_4	fc	log2 (fc)	pval	qval	log10 (qval)	regula tion	signif icant
ENSMUSG00000095675	Ccl21b	chemokine (C-C	6.34	6.73	5.38	4.95	0.11	0.15	0.06	0.11	55.12	5.78	3.58E-57	1.19E-52	-51.92	up	yes
ENSMUSG00000109209	Gm45104	predicted gene	2.00	1.30	1.76	1.89	0.14	0.04	0.08	0.09	19.49	4.28	2.44E-53	4.06E-49	-48.39	up	yes
ENSMUSG00000073878	Gm13304	predicted gene	6.37	4.94	5.22	3.51	0.19	0.15	0.13	0.11	34.47	5.11	1.36E-44	1.51E-40	-39.82	up	yes
ENSMUSG00000098975	Gm27177	predicted gene	0.81	0.85	1.74	2.06	0.05	0.07	0.08	0.05	21.81	4.45	3.64E-44	3.03E-40	-39.52	up	yes
ENSMUSG00000096596	Gm10591	predicted gene	5.53	5.22	4.48	3.09	0.12	0.06	0.09	0.08	51.87	5.70	1.34E-42	8.94E-39	-38.05	up	yes
ENSMUSG00000094065	Ccl21d	chemokine (C-C	4.97	3.95	4.25	3.12	0.10	0.13	0.11	0.06	40.57	5.34	2.05E-41	1.13E-37	-36.95	up	yes
ENSMUSG00000110105	Gm45844	predicted gene	0.04	0.07	0.06	0.07	0.48	0.43	0.50	0.47	0.13	-2.96	1.28E-28	6.07E-25	-24.22	down	yes
ENSMUSG00000097842	9330104G04Rik	RIKEN cDNA 9330	0.23	0.17	0.35	0.34	1.39	1.52	1.60	1.36	0.18	-2.44	7.80E-25	3.24E-21	-20.49	down	yes
ENSMUSG00000035299	Mid1	midline 1 [Sour	0.35	0.27	0.26	0.31	1.39	0.78	1.14	1.53	0.25	-2.01	6.71E-20	2.48E-16	-15.61	down	yes
ENSMUSG00000090338	Gm17081	predicted gene	1.42	0.77	1.13	0.48	0.06	0.09	0.09	0.06	13.18	3.72	9.10E-18	3.03E-14	-13.52	up	yes
ENSMUSG00000053985	Zfp14	zinc finger pro	5.72	5.15	4.46	5.50	2.79	2.63	2.68	2.74	1.92	0.94	2.99E-16	9.03E-13	-12.04	up	no
ENSMUSG00000118458	Gm10599	predicted pseud	1.79	0.83	1.07	0.52	0.09	0.12	0.11	0.09	9.94	3.31	3.25E-15	8.35E-12	-11.08	up	yes
ENSMUSG00000093954	Gm16867	predicted gene,	1.04	0.54	2.17	2.37	0.02	0.06	0.09	0.05	27.66	4.79	3.26E-15	8.35E-12	-11.08	up	yes
ENSMUSG00000107705	Gm45062	predicted gene	0	0	6.24	0	0	0	0	0	15591.60	13.93	3.41E-13	8.09E-10	-9.09	up	yes
ENSMUSG00000066107	Gm12666	predicted gene	1.44	1.10	0.70	0.92	0	0	0	0	10373.24	13.34	1.18E-12	2.61E-09	-8.58	up	yes
ENSMUSG00000044783	Hjurp	Holliday juncti	5.01	4.11	4.29	5.45	7.75	6.77	8.43	7.53	0.62	-0.69	3.32E-12	6.90E-09	-8.16	down	no
ENSMUSG00000073879	Gm5859	predicted pseud	1.12	0.70	0.82	0.41	0.09	0.12	0.13	0.08	7.41	2.89	3.53E-12	6.91E-09	-8.16	up	yes
ENSMUSG00000091542	Gm17167	predicted gene	0.81	0.49	0.52	0.28	0.10	0.09	0.10	0.04	6.42	2.68	5.18E-12	9.57E-09	-8.02	up	yes
ENSMUSG00000094006	Igkv4-59	immunoglobulin	2.87	17.19	0	0	0	0	0	0	50170.09	15.61	8.81E-12	1.54E-08	-7.81	up	yes
ENSMUSG00000029994	Anxa4	annexin A4 [Sou	5.08	3.57	2.47	3.90	1.48	1.53	1.82	1.43	2.40	1.26	1.04E-11	1.73E-08	-7.76	up	yes
ENSMUSG00000074358	Ccdc61	coiled-coil dom	0.93	0.95	0.79	1.05	2.29	1.89	2.01	1.99	0.45	-1.14	1.12E-11	1.77E-08	-7.75	down	yes
ENSMUSG00000096862	Gm13301	predicted gene	0.66	0.45	0.53	0.24	0.05	0.04	0.05	0.03	11.10	3.47	5.07E-11	7.67E-08	-7.12	up	yes
ENSMUSG00000030413	Pglyrp1	peptidoglycan r	1.16	0.74	0.91	0.55	2.49	2.78	2.34	5.70	0.25	-1.99	9.20E-11	1.33E-07	-6.88	down	yes
ENSMUSG00000092232	Gm20521	predicted gene	0	2.22	0	0	0	0	0	0	5553.92	12.44	1.36E-10	1.88E-07	-6.73	up	yes
ENSMUSG00000031562	Dctd	dCMP deaminase	0.90	0.79	0.86	0.90	1.89	1.78	2.18	1.44	0.47	-1.08	2.16E-10	2.83E-07	-6.55	down	yes
ENSMUSG00000096764	Gm21985	predicted gene	0	0	0	0	0	0	0.79	0	0.00	-10.95	2.22E-10	2.83E-07	-6.55	down	yes
ENSMUSG00000021342	Prl	prolactin [Sour	0	1.59	0	0	0	0	0	0	3979.54	11.96	2.30E-10	2.83E-07	-6.55	up	yes
ENSMUSG00000090996	Gm20458	predicted gene	0	0	0	0	0	0	0	0.70	0.00	-10.77	5.01E-10	5.95E-07	-6.23	down	yes
ENSMUSG00000095007	Igkv12-41	immunoglobulin	0	3.48	0	0	0	0	0	0	8709.14	13.09	1.20E-09	1.37E-06	-5.86	up	yes
ENSMUSG00000001240	Ramp2	receptor (calci	2.84	3.46	3.49	4.36	6.12	6.69	5.98	9.50	0.50	-1.00	1.35E-09	1.49E-06	-5.83	down	yes
ENSMUSG00000095351	Igkv3-2	immunoglobulin	3.02	0	0	0	0	0	0	0	7559.13	12.88	1.50E-09	1.58E-06	-5.80	up	yes
ENSMUSG00000053964	Lgals4	lectin, galacto	0.22	0.32	0.21	0.13	0.78	0.83	0.85	1.24	0.24	-2.07	1.52E-09	1.58E-06	-5.80	down	yes
ENSMUSG00000092511	Gm20547	predicted gene	0.85	0	0	0	0	0	0	0	2127.73	11.06	1.65E-09	1.67E-06	-5.78	up	yes
ENSMUSG00000109176	Zfp264	zinc finger pro	0.27	0.42	0.65	0.22	0.05	0.02	0	0.02	17.38	4.12	1.72E-09	1.68E-06	-5.77	up	yes
ENSMUSG00000087067	Gm11532	predicted gene	0	0	0.18	0	0	0	0	0	453.68	8.83	2.19E-09	2.08E-06	-5.68	up	yes
ENSMUSG00000052861	Dnah6	dynein, axonema	0.44	0.36	0.45	0.44	0.16	0.09	0.23	0.15	2.70	1.43	1.51E-08	1.39E-05	-4.86	up	yes
ENSMUSG00000066838	Zfp772	zinc finger pro	2.26	2.16	2.46	1.73	3.64	3.62	4.09	3.62	0.58	-0.80	4.84E-08	4.35E-05	-4.36	down	no
ENSMUSG00000030123	Plxnd1	plexin D1 [Sour	5.94	4.89	5.98	4.73	8.43	8.33	8.40	7.22	0.67	-0.59	5.38E-08	4.71E-05	-4.33	down	no
ENSMUSG00000046688	Tifa	TRAF-interactin	5.52	4.62	4.65	4.93	3.45	2.99	3.24	2.67	1.60	0.68	1.02E-07	8.69E-05	-4.06	up	no
ENSMUSG00000038642	Ctss	cathepsin S [So	235.60	172.70	201.19	224.71	146.02	141.30	155.61	131.80	1.45	0.54	1.34E-07	1.11E-04	-3.95	up	no
ENSMUSG00000107927	Gm44090	predicted gene,	0	0.02	0.03	0	0.31	0.43	0.18	0.27	0.04	-4.63	2.00E-07	1.63E-04	-3.79	down	yes
ENSMUSG00000095562	Erdr1	erythroid diffe	1.09	1.14	0.13	1.70	10.90	7.65	7.90	11.85	0.11	-3.24	3.41E-07	2.70E-04	-3.57	down	yes
ENSMUSG00000035722	Abca7	ATP-binding cas	1.78	1.72	1.75	1.59	2.82	2.47	2.31	2.53	0.67	-0.57	5.11E-07	3.95E-04	-3.40	down	no
ENSMUSG00000030577	Cd22	CD22 antigen [S	2.45	1.22	1.30	1.25	0.77	0.39	0.75	0.56	2.53	1.34	5.24E-07	3.96E-04	-3.40	up	yes
ENSMUSG00000098900	Gm18190	predicted gene,	0	0	0	0	0.28	0.14	0.19	0.15	0.00	-10.88	5.61E-07	4.14E-04	-3.38	down	yes
ENSMUSG00000074280	Gm6166	predicted gene	1.28	29.43	22.90	28.61	0.90	1.80	1.11	0.79	17.85	4.16	7.00E-07	5.06E-04	-3.30	up	yes
ENSMUSG00000095463	Entpd4	ectonucleoside	5.80	5.54	6.95	7.77	4.12	4.57	3.83	2.77	1.70	0.77	7.69E-07	5.44E-04	-3.26	up	no
ENSMUSG00000021091	Serpina3n	serine (or cyst	248.90	182.80	185.58	121.22	51.49	45.44	85.65	81.03	2.80	1.49	8.07E-07	5.59E-04	-3.25	up	yes
ENSMUSG00000007440	Pcdha11	protocadherin a	0.49	0.60	1.28	0.51	1.14	2.25	2.03	2.31	0.37	-1.42	1.12E-06	7.61E-04	-3.12	down	yes
ENSMUSG00000023367	Tmem176a	transmembrane p	9.45	8.58	8.35	7.79	5.47	6.19	6.48	4.97	1.48	0.56	1.44E-06	9.56E-04	-3.02	up	no
ENSMUSG00000001119	Col6a1	collagen, type	5.05	5.21	3.86	6.02	7.52	7.02	7.24	8.51	0.66	-0.59	1.60E-06	1.04E-03	-2.98	down	no
ENSMUSG00000110631	Gm42047	predicted gene,	0.23	0.23	0.20	0.71	1.12	0.94	1.14	0.81	0.34	-1.54	1.73E-06	1.11E-03	-2.96	down	yes
ENSMUSG00000026204	Ptprn	protein tyrosin	75.15	68.54	76.33	71.17	92.71	101.30	87.75	108.89	0.75	-0.42	1.89E-06	1.18E-03	-2.93	down	no
ENSMUSG00000062132	Arhgap33os	Rho GTPase acti	0.17	0.27	0.11	0.16	0	0.02	0	0.02	19.09	4.25	2.03E-06	1.24E-03	-2.91	up	yes
ENSMUSG00000045948	Mrps12	mitochondrial r	13.20	11.90	13.45	14.63	21.25	24.31	17.12	18.51	0.65	-0.61	2.05E-06	1.24E-03	-2.91	down	no
ENSMUSG00000084010	Gm13302	predicted gene	0.73	0.40	0.52	0.26	0.11	0.06	0.11	0.04	6.01	2.59	2.18E-06	1.27E-03	-2.89	up	yes
ENSMUSG00000046056	Sbsn	suprabasin [Sou	1.21	0.72	0.90	1.13	1.92	2.54	1.64	1.62	0.51	-0.97	2.18E-06	1.27E-03	-2.89	down	no
ENSMUSG00000040552	C3ar1	complement comp	3.72	2.51	2.76	3.14	1.93	1.61	2.14	1.50	1.69	0.76	3.03E-06	1.74E-03	-2.76	up	no
ENSMUSG00000074361	C5ar2	complement comp	0.04	0.02	0.04	0	0.15	0.20	0.30	0.21	0.11	-3.13	4.18E-06	2.35E-03	-2.63	down	yes
ENSMUSG00000031246	Sh3bgrl	SH3-binding dom	26.85	27.33	25.00	27.37	19.63	21.21	22.01	19.85	1.29	0.37	4.44E-06	2.46E-03	-2.61	up	no
ENSMUSG00000027858	Tspan2	tetraspanin 2 [	39.10	31.50	27.03	34.19	22.41	21.15	26.38	18.30	1.49	0.58	4.66E-06	2.54E-03	-2.59	up	no
ENSMUSG00000096768	Gm47283	predicted gene,	0.66	0.46	0.05	0.85	5.23	4.64	3.21	5.14	0.11	-3.18	5.19E-06	2.78E-03	-2.56	down	yes
ENSMUSG00000031503	Col4a2	collagen, type	2.72	2.74	3.47	2.57	4.23	3.65	4.75	6.16	0.61	-0.71	5.73E-06	3.03E-03	-2.52	down	no
ENSMUSG00000069184	Zfp72	zinc finger pro	1.99	1.89	1.56	1.86	1.18	1.14	1.13	1.16	1.58	0.66	6.20E-06	3.22E-03	-2.49	up	no
ENSMUSG00000092375	A730060N03Rik	RIKEN cDNA A730	0.38	0.15	0.40	0.16	0.70	0.80	1.19	0.90	0.30	-1.74	6.30E-06	3.22E-03	-2.49	down	yes
ENSMUSG00000118425	Gm50470	predicted gene,	0.83	0.49	0.57	0.34	0.16	0.11	0.15	0.14	4.11	2.04	6.53E-06	3.29E-03	-2.48	up	yes
ENSMUSG00000022899	Slc15a2	solute carrier	5.59	4.07	5.33	4.93	3.57	3.18	3.56	3.49	1.44	0.53	6.78E-06	3.36E-03	-2.47	up	no
ENSMUSG00000005575	Ube2m	ubiquitin-conju	34.46	38.49	35.76	36.37	48.12	57.90	42.84	50.10	0.73	-0.46	6.98E-06	3.41E-03	-2.47	down	no
ENSMUSG00000026177	Slc11a1	solute carrier	3.69	2.93	2.25	3.12	1.76	1.55	2.10	1.21	1.81	0.86	7.87E-06	3.79E-03	-2.42	up	no
ENSMUSG00000098915	Rpl15-ps2	ribosomal prote	0.91	1.18	0.87	0.84	5.50	5.31	3.46	0.87	0.25	-1.99	8.58E-06	4.08E-03	-2.39	down	yes
ENSMUSG00000009281	Rarres2	retinoic acid r	5.06	5.61	4.35	3.82	2.99	2.59	3.20	2.84	1.62	0.70	9.14E-06	4.28E-03	-2.37	up	no
ENSMUSG00000020396	Nefh	neurofilament,	27.51	26.87	26.03	22.45	39.83	31.90	33.66	34.72	0.73	-0.45	9.43E-06	4.35E-03	-2.36	down	no
ENSMUSG00000030237	Slco1a4	solute carrier	8.29	8.49	7.27	8.36	5.37	6.49	6.36	5.18	1.39	0.47	1.01E-05	4.60E-03	-2.34	up	no
ENSMUSG00000118434	Gm13301	predicted gene	1.52	0.98	1.54	0.51	0.17	0.13	0.34	0.26	5.07	2.34	1.04E-05	4.68E-03	-2.33	up	yes
ENSMUSG00000073664	Nbeal1	neurobeachin li	0.81	0.66	0.76	0.78	0.49	0.44	0.63	0.42	1.53	0.61	1.16E-05	5.15E-03	-2.29	up	no
ENSMUSG00000079494	Nat8f5	N-acetyltransfe	0.49	0.33	0.17	0.26	0.91	2.00	1.56	0.70	0.24	-2.05	1.20E-05	5.23E-03	-2.28	down	yes
ENSMUSG00000019773	Fbxo5	F-box protein 5	1.42	0.98	1.26	1.05	0.49	0.35	0.69	0.33	2.53	1.34	1.22E-05	5.27E-03	-2.28	up	yes
ENSMUSG00000028028	Alpk1	alpha-kinase 1	1.37	0.60	0.81	0.90	0.29	0.48	0.53	0.35	2.23	1.15	1.27E-05	5.40E-03	-2.27	up	yes
ENSMUSG00000020932	Gfap	glial fibrillar	545.98	404.34	385.09	394.91	252.75	261.69	365.94	273.41	1.50	0.58	1.34E-05	5.65E-03	-2.25	up	no
ENSMUSG00000026830	Ermn	ermin, ERM-like	32.70	35.82	27.03	28.98	23.18	17.04	23.40	13.44	1.62	0.69	1.42E-05	5.88E-03	-2.23	up	no
ENSMUSG00000050541	Adra1b	adrenergic rece	4.38	3.39	4.58	2.99	5.56	6.19	6.15	5.75	0.65	-0.62	1.57E-05	6.43E-03	-2.19	down	no
ENSMUSG00000057021	Vmn2r-ps159	vomeronasal 2,	0	0.15	0.09	0.16	0	0	0	0	990.18	9.95	1.63E-05	6.63E-03	-2.18	up	yes
ENSMUSG00000039194	Rlbp1	retinaldehyde b	7.58	8.09	6.74	8.32	5.59	4.63	5.88	3.66	1.55	0.64	2.05E-05	8.20E-03	-2.09	up	no
ENSMUSG00000033964	Zbtb41	zinc finger and	3.07	3.15	3.09	3.46	2.36	2.08	2.68	2.23	1.37	0.45	2.33E-05	9.21E-03	-2.04	up	no
ENSMUSG00000062760	Shisal1	shisa like 1 [S	6.20	6.55	6.36	6.02	8.16	7.53	8.10	7.82	0.80	-0.33	2.44E-05	9.50E-03	-2.02	down	no
ENSMUSG00000043773	1700048O20Rik	RIKEN cDNA 1700	1.91	3.09	2.73	2.73	4.77	4.73	3.66	4.27	0.60	-0.73	2.46E-05	9.50E-03	-2.02	down	no
ENSMUSG00000074513	Arfip1	ADP-ribosylatic	12.27	11.47	9.69	10.64	8.31	6.38	8.73	7.30	1.43	0.52	3.01E-05	1.15E-02	-1.94	up	no
ENSMUSG00000033597	Caskin1	CASK interactin	10.85	10.30	10.31	9.38	13.40	12.45	12.63	12.95	0.79	-0.33	3.07E-05	1.15E-02	-1.94	down	no
ENSMUSG00000109205	Gm44954	predicted gene	1.54	3.35	3.92	2.33	0.29	0.53	0.78	0.45	5.43	2.44	3.11E-05	1.15E-02	-1.94	up	yes
ENSMUSG00000022565	Plec	plectin [Source	4.36	3.56	3.72	3.65	4.65	4.76	4.95	5.36	0.78	-0.37	3.12E-05	1.15E-02	-1.94	down	no
ENSMUSG00000070923	Klhl9	kelch-like 9 [S	21.83	22.54	23.51	24.54	19.20	15.84	18.54	17.38	1.30	0.38	3.28E-05	1.20E-02	-1.92	up	no
ENSMUSG00000059751	Rps3a3	ribosomal prote	1.13	1.86	1.21	1.26	61.02	2.45	1.77	60.21	0.04	-4.52	3.31E-05	1.20E-02	-1.92	down	yes
ENSMUSG00000034156	Tspoap1	TSPO associated	10.93	8.90	10.83	10.22	12.50	13.56	12.47	14.55	0.77	-0.38	3.54E-05	1.25E-02	-1.90	down	no
ENSMUSG00000036594	H2-Aa	histocompatibil	4.17	3.89	5.22	2.98	1.37	2.37	3.04	1.57	1.95	0.96	3.55E-05	1.25E-02	-1.90	up	no
ENSMUSG00000052026	Slc6a7	solute carrier	14.27	13.89	13.24	15.63	17.91	21.43	16.40	20.12	0.75	-0.41	3.57E-05	1.25E-02	-1.90	down	no
ENSMUSG00000041483	Zfp281	zinc finger pro	8.72	7.34	7.93	8.74	5.92	5.68	7.05	5.60	1.35	0.43	3.63E-05	1.26E-02	-1.90	up	no
ENSMUSG00000067869	Tcea1-ps1	transcription e	10.99	13.82	14.22	9.07	4.83	7.15	2.99	7.75	2.12	1.08	3.82E-05	1.30E-02	-1.88	up	yes
ENSMUSG00000045545	Krt14	keratin 14 [Sou	0	0	0	0	0.05	0.45	0.16	0	0.00	-10.70	3.84E-05	1.30E-02	-1.88	down	yes
ENSMUSG00000061119	Prcp	prolylcarboxype	2.53	2.42	2.32	3.26	1.58	1.78	2.05	1.63	1.50	0.58	4.29E-05	1.44E-02	-1.84	up	no

Table S2G: Selection of genes altered in PS/E4H vs E4H mice. FPKM values of genes in individual mice (indicated in underscored numeral), along with fold change (fc) value, p value adjusted for false discovery rate of 0.05 (q value, qval) are presented. N=3-4 mice/group. PE4H, PS19 mice homozygous for APOE4.

gene_id	gene_name	Description	FPKM. PE4H_1	FPKM. PE4H_2	FPKM. PE4H_3	FPKM. PE4H_4	FPKM. E4H_1	FPKM. E4H_2	FPKM. E4H_3	fc	log2 (fc)	pval	qval	log10 (qval)	regulat ion	signifi cant
ENSMUSG00000020932	Gfap	glial fibrillary a	545.98	404.34	385.09	394.91	51.50	51.45	51.25	8.42	3.07	1.66E-182	5.56E-178	-177.25	up	yes
ENSMUSG00000036887	C1qa	complement compone	203.76	159.52	159.22	166.67	32.88	35.00	33.12	5.12	2.36	4.92E-142	8.24E-138	-137.08	up	yes
ENSMUSG00000038642	Ctss	cathepsin S [Sourc	235.60	172.70	201.19	224.71	51.04	49.94	56.47	3.97	1.99	1.57E-95	1.75E-91	-90.76	up	yes
ENSMUSG00000068129	Cst7	cystatin F (leukoc	22.41	10.73	9.29	11.62	0.16	0.27	0.18	66.74	6.06	3.57E-72	2.99E-68	-67.52	up	yes
ENSMUSG00000036905	C1qb	complement compone	180.35	119.35	128.74	156.31	37.34	38.76	36.27	3.90	1.96	1.61E-64	1.08E-60	-59.97	up	yes
ENSMUSG00000046805	Mpeg1	macrophage express	46.93	31.96	31.03	31.02	7.25	7.43	8.29	4.60	2.20	2.72E-62	1.52E-58	-57.82	up	yes
ENSMUSG00000022037	Clu	clusterin [Source	440.33	444.20	452.07	458.09	235.74	213.62	235.05	1.97	0.98	2.71E-59	1.30E-55	-54.89	up	no
ENSMUSG00000024610	Cd74	CD74 antigen (inva	28.89	28.58	38.64	18.90	3.83	3.65	3.12	8.14	3.02	1.79E-57	7.47E-54	-53.13	up	yes
ENSMUSG00000023992	Trem2	triggering recepto	10.36	8.14	7.17	9.57	2.47	2.26	2.11	3.87	1.95	3.64E-57	1.35E-53	-52.87	up	yes
ENSMUSG00000036896	C1qc	complement compone	88.76	56.92	56.96	69.56	16.98	17.11	17.39	3.97	1.99	1.53E-54	5.14E-51	-50.29	up	yes
ENSMUSG00000030579	Tyrobp	TYRO protein tyros	72.12	63.93	51.96	54.02	16.67	14.82	16.32	3.80	1.92	5.98E-53	1.82E-49	-48.74	up	yes
ENSMUSG00000018927	Ccl6	chemokine (C-C mot	6.57	6.23	5.61	5.55	0.91	0.65	1.05	6.90	2.79	2.02E-52	5.63E-49	-48.25	up	yes
ENSMUSG00000073411	H2-D1	histocompatibility	60.40	42.41	47.19	58.58	17.17	16.97	17.06	3.06	1.61	1.04E-51	2.68E-48	-47.57	up	yes
ENSMUSG00000040552	C3ar1	complement compone	3.72	2.51	2.76	3.14	0.56	0.54	0.58	5.41	2.43	6.41E-51	1.53E-47	-46.81	up	yes
ENSMUSG00000038147	Cd84	CD84 antigen [Sour	3.38	2.29	2.29	2.49	0.37	0.48	0.47	5.91	2.56	6.38E-50	1.42E-46	-45.85	up	yes
ENSMUSG00000021665	Hexb	hexosaminidase B	170.48	134.18	128.19	173.64	55.15	57.06	57.78	2.68	1.42	3.73E-48	7.79E-45	-44.11	up	yes
ENSMUSG00000033685	Ucp2	uncoupling protein	8.29	7.84	7.37	7.77	3.37	2.87	2.65	2.64	1.40	5.12E-47	1.01E-43	-43.00	up	yes
ENSMUSG00000059498	Fcgr3	Fc receptor, IgG,	24.18	19.72	29.30	23.99	6.86	7.33	6.74	3.48	1.80	2.47E-44	4.59E-41	-40.34	up	yes
ENSMUSG00000073421	H2-Ab1	histocompatibility	3.93	4.05	4.37	2.91	0.58	0.59	0.57	6.56	2.71	3.55E-44	6.25E-41	-40.20	up	yes
ENSMUSG00000026656	Fcgr2b	Fc receptor, IgG,	4.24	2.93	3.40	3.27	0.73	0.90	0.66	4.53	2.18	7.10E-44	1.19E-40	-39.93	up	yes
ENSMUSG00000079293	Clec7a	C-type lectin doma	12.01	8.65	7.53	7.31	0.35	0.26	0.12	36.11	5.17	1.05E-43	1.67E-40	-39.78	up	yes
ENSMUSG00000006519	Cyba	cytochrome b-245,	15.72	14.34	14.44	14.17	3.43	3.46	4.58	3.84	1.94	2.59E-43	3.93E-40	-39.41	up	yes
ENSMUSG00000026701	Prdx6	peroxiredoxin 6 [S	90.40	77.74	70.80	83.53	39.50	37.62	38.21	2.10	1.07	4.48E-43	6.52E-40	-39.19	up	yes
ENSMUSG00000007891	Ctsd	cathepsin D [Sourc	713.67	477.17	441.43	534.96	171.20	160.32	172.41	3.23	1.69	6.78E-43	9.46E-40	-39.02	up	yes
ENSMUSG00000000682	Cd52	CD52 antigen [Sour	8.64	6.77	5.62	6.31	1.12	0.86	0.84	7.28	2.86	9.97E-43	1.33E-39	-38.87	up	yes
ENSMUSG00000016256	Ctsz	cathepsin Z [Sourc	102.32	74.47	66.52	80.50	27.33	27.14	26.67	2.99	1.58	1.38E-41	1.77E-38	-37.75	up	yes
ENSMUSG00000028581	Laptm5	lysosomal-associat	23.18	19.26	18.46	20.35	7.92	8.41	9.17	2.39	1.26	1.46E-41	1.81E-38	-37.74	up	yes
ENSMUSG00000017009	Sdc4	syndecan 4 [Source	46.29	39.26	44.21	42.60	20.11	15.94	18.24	2.38	1.25	2.26E-41	2.71E-38	-37.57	up	yes
ENSMUSG00000018774	Cd68	CD68 antigen [Sour	44.87	34.69	23.91	33.08	7.96	7.10	7.97	4.45	2.15	6.21E-41	7.17E-38	-37.14	up	yes
ENSMUSG00000058715	Fcer1g	Fc receptor, IgE,	28.08	21.74	20.26	21.66	6.84	5.35	5.48	3.89	1.96	9.45E-41	1.05E-37	-36.98	up	yes
ENSMUSG00000027995	Tlr2	toll-like recepto	4.44	2.76	2.74	3.85	0.47	0.54	0.59	6.43	2.69	1.68E-39	1.81E-36	-35.74	up	yes
ENSMUSG00000030560	Ctsc	cathepsin C [Sourc	4.68	4.15	3.79	3.71	1.47	1.29	1.43	2.92	1.55	2.59E-38	2.71E-35	-34.57	up	yes
ENSMUSG00000015852	Fcrls	Fc receptor-like S	27.13	18.71	21.48	27.54	8.19	8.25	7.78	2.94	1.55	2.14E-37	2.17E-34	-33.66	up	yes
ENSMUSG00000030789	Itgax	integrin alpha X	7.53	3.67	3.96	4.09	0.21	0.11	0.10	34.11	5.09	2.89E-37	2.84E-34	-33.55	up	yes
ENSMUSG00000021423	Ly86	lymphocyte antigen	9.29	6.73	5.72	8.34	1.44	1.81	2.09	4.22	2.08	1.10E-35	1.05E-32	-31.98	up	yes
ENSMUSG00000021242	Npc2	NPC intracellular	23.75	21.12	19.56	22.52	11.64	10.30	11.15	1.97	0.98	3.19E-35	2.97E-32	-31.53	up	no
ENSMUSG00000022548	Apod	apolipoprotein D	249.64	248.73	215.72	189.21	87.89	65.15	87.15	2.82	1.50	4.69E-35	4.25E-32	-31.37	up	yes
ENSMUSG00000015950	Ncf1	neutrophil cytoso	5.47	4.66	4.45	5.18	1.77	1.80	1.44	2.95	1.56	7.33E-35	6.46E-32	-31.19	up	yes
ENSMUSG00000019970	Sgk1	serum/glucocortico	45.63	36.66	32.55	42.40	16.55	12.88	11.51	2.88	1.53	1.67E-34	1.43E-31	-30.84	up	yes
ENSMUSG00000097415	AU020206	expressed sequence	5.33	4.60	4.35	5.48	1.01	1.52	1.44	3.73	1.90	1.16E-33	9.73E-31	-30.01	up	yes
ENSMUSG00000004707	Ly9	lymphocyte antigen	3.38	2.10	2.45	1.83	0.36	0.23	0.31	8.07	3.01	1.44E-33	1.18E-30	-29.93	up	yes
ENSMUSG00000059336	Slc14a1	solute carrier fam	9.30	7.95	6.28	9.08	2.60	2.19	2.87	3.20	1.68	1.56E-33	1.24E-30	-29.91	up	yes
ENSMUSG00000021998	Lcp1	lymphocyte cytosol	3.09	2.50	2.50	2.96	1.12	1.12	1.19	2.42	1.27	3.47E-33	2.70E-30	-29.57	up	yes
ENSMUSG00000004730	Adgre1	adhesion G protein	8.89	5.50	6.64	7.27	1.94	2.06	2.18	3.43	1.78	9.82E-33	7.47E-30	-29.13	up	yes
ENSMUSG00000004266	Ptpn6	protein tyrosine p	5.47	4.23	4.03	4.51	1.40	1.39	1.45	3.23	1.69	5.27E-32	3.92E-29	-28.41	up	yes
ENSMUSG00000020120	Plek	pleckstrin [Source	13.70	9.43	10.24	12.12	3.67	3.92	4.58	2.80	1.49	5.49E-32	3.99E-29	-28.40	up	yes
ENSMUSG00000036594	H2-Aa	histocompatibility	4.17	3.89	5.22	2.98	0.44	0.47	0.85	6.90	2.79	5.61E-32	4.00E-29	-28.40	up	yes
ENSMUSG00000015355	Cd48	CD48 antigen [Sour	3.91	4.79	3.61	3.80	0.60	0.52	0.70	6.62	2.73	6.87E-32	4.79E-29	-28.32	up	yes
ENSMUSG00000060802	B2m	beta-2 microglobul	244.98	202.27	215.59	297.08	107.23	102.84	94.64	2.36	1.24	1.70E-31	1.16E-28	-27.94	up	yes
ENSMUSG00000000290	Itgb2	integrin beta 2 [S	10.69	6.87	6.30	6.80	1.95	2.02	1.85	3.95	1.98	3.67E-31	2.46E-28	-27.61	up	yes
ENSMUSG00000079037	Prnp	prion protein [Sou	654.04	578.21	706.09	780.93	324.89	326.99	353.81	2.03	1.02	4.25E-31	2.79E-28	-27.55	up	yes
ENSMUSG00000026480	Ncf2	neutrophil cytoso	1.42	1.33	1.47	1.21	0.43	0.42	0.46	3.12	1.64	7.13E-31	4.59E-28	-27.34	up	yes
ENSMUSG00000029810	Tmem176b	transmembrane prot	25.67	26.01	23.53	22.11	12.20	11.18	10.86	2.13	1.09	8.91E-31	5.63E-28	-27.25	up	yes
ENSMUSG00000025351	Cd63	CD63 antigen [Sour	42.15	37.11	27.57	36.63	13.46	12.77	15.35	2.59	1.37	9.49E-31	5.88E-28	-27.23	up	yes
ENSMUSG00000032359	Ctsh	cathepsin H [Sourc	8.28	6.51	4.88	6.81	1.92	1.81	2.19	3.36	1.75	1.41E-30	8.58E-28	-27.07	up	yes
ENSMUSG00000030342	Cd9	CD9 antigen [Sourc	60.52	47.49	36.34	57.11	17.99	15.80	18.48	2.89	1.53	5.42E-30	3.24E-27	-26.49	up	yes
ENSMUSG00000015340	Cybb	cytochrome b-245,	1.39	1.05	1.06	0.55	0.08	0.10	0.07	12.41	3.63	5.54E-30	3.25E-27	-26.49	up	yes
ENSMUSG00000023367	Tmem176a	transmembrane prot	9.45	8.58	8.35	7.79	3.64	2.96	3.80	2.47	1.30	5.50E-29	3.17E-26	-25.50	up	yes
ENSMUSG00000024300	Myo1f	myosin IF [Source	2.66	1.96	1.68	1.98	0.41	0.40	0.56	4.57	2.19	6.95E-29	3.94E-26	-25.40	up	yes
ENSMUSG00000015947	Fcgr1	Fc receptor, IgG,	4.46	3.40	4.00	3.81	1.19	1.06	1.29	3.31	1.73	7.73E-29	4.31E-26	-25.37	up	yes
ENSMUSG00000041559	Fmod	fibromodulin [Sour	12.33	11.16	10.37	12.54	5.96	5.11	4.83	2.19	1.13	9.95E-29	5.46E-26	-25.26	up	yes
ENSMUSG00000029622	Arpc1b	actin related prot	6.73	5.64	4.80	5.66	2.25	2.31	2.40	2.46	1.30	1.71E-28	9.21E-26	-25.04	up	yes
ENSMUSG00000034957	Cebpa	CCAAT/enhancer bin	7.73	6.49	6.76	8.01	3.24	2.71	3.02	2.42	1.28	7.01E-28	3.72E-25	-24.43	up	yes
ENSMUSG00000028859	Csf3r	colony stimulating	4.85	3.41	4.28	4.27	1.31	1.54	1.27	3.07	1.62	1.01E-27	5.30E-25	-24.28	up	yes
ENSMUSG00000040747	Cd53	CD53 antigen [Sour	10.02	8.35	8.19	9.49	3.56	2.85	3.64	2.69	1.43	3.36E-27	1.73E-24	-23.76	up	yes
ENSMUSG00000024621	Csf1r	colony stimulating	34.41	28.88	31.19	29.80	17.48	15.62	17.58	1.84	0.88	6.51E-27	3.30E-24	-23.48	up	no
ENSMUSG00000027848	Olfml3	olfactomedin-like	8.01	6.27	6.26	7.80	3.33	3.39	3.28	2.13	1.09	1.94E-26	9.68E-24	-23.01	up	yes
ENSMUSG00000022817	Itgb5	integrin beta 5 [S	30.13	24.87	27.07	24.62	13.45	11.36	13.69	2.08	1.06	2.81E-26	1.39E-23	-22.86	up	yes
ENSMUSG00000015090	Ptgds	prostaglandin D2 s	605.29	704.97	647.09	641.99	353.67	261.57	324.41	2.07	1.05	3.03E-26	1.47E-23	-22.83	up	yes
ENSMUSG00000040997	Abhd4	abhydrolase domain	34.97	36.70	33.57	34.49	19.98	18.04	21.10	1.77	0.83	3.07E-26	1.47E-23	-22.83	up	no
ENSMUSG00000029723	Tsc22d4	TSC22 domain famil	12.23	12.47	12.44	10.78	6.37	5.69	6.41	1.95	0.96	4.49E-26	2.12E-23	-22.67	up	no
ENSMUSG00000035805	Mlc1	megalencephalic le	100.00	94.04	93.32	94.74	56.24	51.97	61.39	1.69	0.76	8.51E-26	3.95E-23	-22.40	up	no
ENSMUSG00000044811	Cd300c2	CD300C molecule 2	9.99	6.49	8.18	8.81	2.18	2.70	2.11	3.59	1.84	1.92E-25	8.82E-23	-22.05	up	yes
ENSMUSG00000031762	Mt2	metallothionein 2	264.00	232.88	197.63	169.31	88.31	85.23	73.88	2.62	1.39	2.13E-25	9.65E-23	-22.02	up	yes
ENSMUSG00000044583	Tlr7	toll-like recepto	2.63	3.19	2.25	2.15	0.69	0.74	0.65	3.67	1.88	2.82E-25	1.26E-22	-21.90	up	yes
ENSMUSG00000022323	Rida	reactive intermedi	17.93	18.49	17.21	19.67	10.69	9.13	10.49	1.81	0.86	4.78E-25	2.10E-22	-21.68	up	no
ENSMUSG00000021477	Ctsl	cathepsin L [Sourc	27.90	24.42	24.16	28.39	14.78	12.83	15.75	1.81	0.86	8.27E-24	3.59E-21	-20.44	up	no
ENSMUSG00000030341	Tnfrsf1a	tumor necrosis fac	9.53	7.26	8.07	7.68	3.01	3.69	3.13	2.48	1.31	1.42E-23	6.10E-21	-20.21	up	yes
ENSMUSG00000041548	Hspb8	heat shock protein	18.33	18.38	13.76	16.49	7.53	6.54	7.87	2.29	1.19	5.93E-23	2.51E-20	-19.60	up	yes
ENSMUSG00000021939	Ctsb	cathepsin B [Sourc	201.84	166.40	165.19	184.14	108.22	102.80	111.85	1.67	0.74	9.84E-23	4.12E-20	-19.39	up	no
ENSMUSG00000028874	Fgr	FGR proto-oncogene	0.85	0.71	0.44	0.57	0.03	0.06	0.03	15.39	3.94	1.08E-22	4.45E-20	-19.35	up	yes
ENSMUSG00000015839	Nfe2l2	nuclear factor, er	8.62	8.22	7.09	9.30	4.10	4.38	3.93	2.01	1.01	1.25E-22	5.12E-20	-19.29	up	yes
ENSMUSG00000073418	C4b	complement compone	77.57	45.49	38.11	48.10	7.70	7.55	8.42	6.63	2.73	1.27E-22	5.14E-20	-19.29	up	yes
ENSMUSG00000051439	Cd14	CD14 antigen [Sour	5.32	4.36	4.99	3.68	0.87	1.27	1.26	4.05	2.02	1.44E-22	5.76E-20	-19.24	up	yes
ENSMUSG00000079484	Phyhd1	phytanoyl-CoA diox	7.70	9.09	12.64	9.24	3.02	3.16	3.50	2.99	1.58	1.49E-22	5.87E-20	-19.23	up	yes
ENSMUSG00000026424	Gpr37l1	G protein-coupled	116.12	116.57	117.23	115.17	72.69	65.52	77.03	1.62	0.70	2.44E-22	9.48E-20	-19.02	up	no
ENSMUSG00000026365	Cfh	complement compone	6.25	5.89	6.42	6.03	3.48	2.92	3.53	1.86	0.89	3.48E-22	1.34E-19	-18.87	up	no
ENSMUSG00000018920	Cxcl16	chemokine (C-X-C m	3.60	2.09	1.82	2.09	0.43	0.42	0.50	5.31	2.41	4.28E-22	1.63E-19	-18.79	up	yes
ENSMUSG00000052336	Cx3cr1	chemokine (C-X3-C	20.54	16.69	17.26	18.23	10.88	9.48	9.43	1.83	0.87	4.48E-22	1.69E-19	-18.77	up	no
ENSMUSG00000079419	Ms4a6c	membrane-spanning	2.47	1.36	2.06	1.07	0.14	0.18	0.15	11.15	3.48	5.09E-22	1.89E-19	-18.72	up	yes
ENSMUSG00000046688	Tifa	TRAF-interacting p	5.52	4.62	4.65	4.93	1.83	1.89	2.36	2.43	1.28	5.93E-22	2.18E-19	-18.66	up	yes
ENSMUSG00000018008	Cyth4	cytohesin 4 [Sourc	4.05	3.06	3.35	3.30	1.66	1.57	1.62	2.13	1.09	8.06E-22	2.93E-19	-18.53	up	yes
ENSMUSG00000064267	Hvcn1	hydrogen voltage-g	3.23	2.59	2.16	2.21	0.78	0.46	0.62	4.08	2.03	9.63E-22	3.47E-19	-18.46	up	yes
ENSMUSG00000026177	Slc11a1	solute carrier fam	3.69	2.93	2.25	3.12	0.45	0.83	0.78	4.35	2.12	1.04E-21	3.71E-19	-18.43	up	yes
ENSMUSG00000052160	Pld4	phospholipase D fa	6.50	5.16	4.70	6.64	1.83	2.47	2.14	2.68	1.42	1.77E-21	6.23E-19	-18.21	up	yes
ENSMUSG00000020399	Havcr2	hepatitis A virus	3.60	3.57	3.12	3.08	1.05	1.30	1.25	2.79	1.48	2.25E-21	7.84E-19	-18.11	up	yes
ENSMUSG00000033488	Cryzl2	crystallin zeta li	6.96	6.59	6.05	6.88	3.12	2.99	3.49	2.07	1.05	3.16E-21	1.09E-18	-17.96	up	yes
ENSMUSG00000024397	Aif1	allograft inflamma	9.76	7.28	5.97	8.34	2.21	2.45	2.77	3.16	1.66	4.01E-21	1.37E-18	-17.86	up	yes
ENSMUSG00000089929	Bcl2a1b	B cell leukemia/ly	6.41	4.21	3.96	5.66	0.96	0.91	1.03	5.23	2.39	5.48E-21	1.85E-18	-17.73	up	yes

Table S2H: Selection of genes altered in PS/E3H vs E3H mice. FPKM values of genes in individual mice (indicated in underscored numeral), along with fold change (fc) value, p value adjusted for false discovery rate of 0.05 (q value, qval) are presented. N=3-4 mice/group. PE3H, PS19 mice homozygous for APOE3.

gene_id	gene_name	Description	FPKM. PE3H_1	FPKM. PE3H_2	FPKM. PE3H_3	FPKM. PE3H_4	FPKM. E3H_1	FPKM. E3H_2	FPKM. E3H_3	fc	log2 (fc)	pval	qval	log10 (qval)	regulation	significant
ENSMUSG00000030789	Itgax	integrin alpha	2.88	4.65	4.35	3.28	0.22	0.25	0.15	18.05	4.17	5.41E-98	1.82E-93	-92.74	up	yes
ENSMUSG00000068129	Cst7	cystatin F (leu	7.60	11.75	15.76	12.52	0.42	0.27	0.50	30.28	4.92	5.65E-76	9.53E-72	-71.02	up	yes
ENSMUSG00000079293	Clec7a	C-type lectin d	3.81	8.09	7.15	8.06	0.36	0.40	0.29	19.31	4.27	2.16E-75	2.43E-71	-70.61	up	yes
ENSMUSG00000015852	Fcrls	Fc receptor-lik	20.55	25.53	28.35	25.25	8.37	9.58	9.79	2.69	1.43	1.29E-46	1.08E-42	-41.96	up	yes
ENSMUSG00000030124	Lag3	lymphocyte-acti	12.46	13.50	11.75	11.63	4.32	3.50	3.28	3.33	1.74	1.64E-41	1.11E-37	-36.96	up	yes
ENSMUSG00000036887	C1qa	complement comp	86.57	148.90	140.49	155.87	41.90	39.09	39.93	3.30	1.72	4.56E-34	2.57E-30	-29.59	up	yes
ENSMUSG00000024610	Cd74	CD74 antigen (i	12.01	17.51	18.23	22.87	4.05	4.28	4.87	4.01	2.00	3.18E-33	1.53E-29	-28.82	up	yes
ENSMUSG00000036905	C1qb	complement comp	87.01	105.74	130.46	142.18	42.00	40.18	36.48	2.94	1.56	5.81E-33	2.45E-29	-28.61	up	yes
ENSMUSG00000020932	Gfap	glial fibrillar	206.22	390.94	365.89	418.36	54.27	57.79	53.91	6.24	2.64	1.48E-32	5.53E-29	-28.26	up	yes
ENSMUSG00000036896	C1qc	complement comp	39.79	50.22	57.24	70.04	20.29	17.59	18.23	2.90	1.54	8.83E-28	2.98E-24	-23.53	up	yes
ENSMUSG00000007891	Ctsd	cathepsin D [So	317.84	509.94	501.51	556.62	175.08	188.03	180.72	2.60	1.38	1.99E-27	6.10E-24	-23.21	up	yes
ENSMUSG00000031762	Mt2	metallothionein	165.58	160.63	210.82	271.67	72.18	66.13	65.60	2.97	1.57	3.55E-27	9.99E-24	-23.00	up	yes
ENSMUSG00000023992	Trem2	triggering rece	5.69	8.09	7.72	8.45	3.03	2.25	2.79	2.78	1.48	1.23E-25	3.19E-22	-21.50	up	yes
ENSMUSG00000030579	Tyrobp	TYRO protein ty	35.11	66.70	57.32	67.84	16.35	17.19	17.92	3.31	1.73	5.54E-25	1.33E-21	-20.87	up	yes
ENSMUSG00000038642	Ctss	cathepsin S [So	101.97	167.65	171.67	193.91	54.75	57.72	60.68	2.75	1.46	1.15E-24	2.58E-21	-20.59	up	yes
ENSMUSG00000030342	Cd9	CD9 antigen [So	29.92	44.04	43.93	49.48	16.73	18.14	16.89	2.43	1.28	1.50E-24	3.17E-21	-20.50	up	yes
ENSMUSG00000018774	Cd68	CD68 antigen [S	16.48	31.90	31.25	36.60	8.02	8.58	7.71	3.59	1.84	1.37E-23	2.72E-20	-19.56	up	yes
ENSMUSG00000005142	Man2b1	mannosidase 2,	9.78	10.28	11.46	10.78	6.40	6.75	6.18	1.64	0.71	3.49E-23	6.54E-20	-19.18	up	no
ENSMUSG00000018927	Ccl6	chemokine (C-C	4.54	6.73	6.67	9.58	1.54	0.77	1.38	5.60	2.49	5.70E-23	1.01E-19	-18.99	up	yes
ENSMUSG00000031765	Mt1	metallothionein	122.83	132.11	160.34	182.96	72.25	64.05	62.35	2.26	1.18	7.25E-23	1.22E-19	-18.91	up	yes
ENSMUSG00000020674	Pxdn	peroxidasin [So	2.84	3.61	3.54	2.91	6.38	6.34	5.91	0.52	-0.95	1.24E-21	2.00E-18	-17.70	down	no
ENSMUSG00000016256	Ctsz	cathepsin Z [So	46.62	77.14	67.34	81.13	27.08	29.51	27.87	2.42	1.27	4.71E-21	7.23E-18	-17.14	up	yes
ENSMUSG00000021423	Ly86	lymphocyte anti	3.87	5.32	6.20	6.91	1.92	1.71	2.02	2.96	1.56	9.57E-21	1.40E-17	-16.85	up	yes
ENSMUSG00000021342	Prl	prolactin [Sour	0	0	0	0	2.98	2.03	1.34	0.00	-14.37	1.37E-20	1.93E-17	-16.71	down	yes
ENSMUSG00000030577	Cd22	CD22 antigen [S	0.44	1.21	1.03	0.94	0.05	0.13	0.10	9.48	3.24	1.55E-20	2.09E-17	-16.68	up	yes
ENSMUSG00000017009	Sdc4	syndecan 4 [Sou	30.90	33.54	42.36	41.70	16.99	20.75	18.50	1.98	0.99	3.12E-20	4.04E-17	-16.39	up	no
ENSMUSG00000027848	Olfml3	olfactomedin-li	5.78	6.79	6.27	6.87	3.42	3.81	3.78	1.75	0.81	1.03E-19	1.29E-16	-15.89	up	no
ENSMUSG00000112148	Lilrb4a	leukocyte immun	0.46	1.99	1.34	1.75	0.12	0.16	0.17	9.45	3.24	1.25E-19	1.50E-16	-15.82	up	yes
ENSMUSG00000033685	Ucp2	uncoupling prot	5.63	6.50	7.48	7.59	3.54	3.49	2.83	2.07	1.05	1.98E-19	2.30E-16	-15.64	up	yes
ENSMUSG00000021665	Hexb	hexosaminidase	91.63	127.50	138.90	139.87	61.66	61.52	64.66	1.99	0.99	2.95E-19	3.32E-16	-15.48	up	no
ENSMUSG00000026656	Fcgr2b	Fc receptor, Ig	1.58	3.41	3.24	4.12	0.78	0.81	0.71	4.02	2.01	4.37E-19	4.76E-16	-15.32	up	yes
ENSMUSG00000022817	Itgb5	integrin beta 5	19.50	27.26	25.13	25.21	13.62	14.12	12.88	1.79	0.84	2.63E-18	2.73E-15	-14.56	up	no
ENSMUSG00000015340	Cybb	cytochrome b-24	0.41	1.27	1.25	1.58	0.15	0.15	0.14	7.58	2.92	2.67E-18	2.73E-15	-14.56	up	yes
ENSMUSG00000030701	Plekhb1	pleckstrin homo	126.98	147.90	145.66	149.97	89.59	102.75	91.91	1.51	0.59	8.34E-18	8.27E-15	-14.08	up	no
ENSMUSG00000079037	Prnp	prion protein [	662.54	561.58	570.67	608.60	351.93	358.11	381.50	1.65	0.72	2.31E-17	2.23E-14	-13.65	up	no
ENSMUSG00000059498	Fcgr3	Fc receptor, Ig	12.89	17.60	22.02	23.24	7.35	8.35	7.05	2.50	1.32	4.07E-17	3.81E-14	-13.42	up	yes
ENSMUSG00000036833	Pnpla7	patatin-like ph	2.83	3.41	3.46	3.75	1.75	1.73	1.87	1.89	0.92	6.09E-17	5.55E-14	-13.26	up	no
ENSMUSG00000064267	Hvcn1	hydrogen voltag	1.41	2.27	2.57	3.21	0.59	0.39	0.64	4.39	2.13	7.15E-17	6.35E-14	-13.20	up	yes
ENSMUSG00000036594	H2-Aa	histocompatibil	1.47	3.01	2.82	3.64	0.76	0.62	0.67	4.02	2.01	8.51E-17	7.37E-14	-13.13	up	yes
ENSMUSG00000040055	Gjb6	gap junction pr	24.96	28.85	36.61	32.77	15.85	18.08	16.86	1.82	0.86	1.14E-16	9.65E-14	-13.02	up	no
ENSMUSG00000028874	Fgr	FGR proto-oncog	0.29	0.75	0.56	0.86	0.05	0.05	0.06	11.10	3.47	2.34E-16	1.92E-13	-12.72	up	yes
ENSMUSG00000020120	Plek	pleckstrin [Sou	6.75	9.66	10.26	12.01	4.42	4.00	4.37	2.27	1.18	3.95E-16	3.10E-13	-12.51	up	yes
ENSMUSG00000021702	Thbs4	thrombospondin	4.46	5.28	7.45	4.67	2.02	2.53	1.87	2.55	1.35	3.95E-16	3.10E-13	-12.51	up	yes
ENSMUSG00000044811	Cd300c2	CD300C molecule	4.61	8.32	9.36	8.50	2.19	2.13	2.65	3.32	1.73	5.47E-16	4.19E-13	-12.38	up	yes
ENSMUSG00000071637	Cebpd	CCAAT/enhancer	1.84	1.70	1.92	2.64	0.65	0.77	0.58	3.04	1.60	7.44E-16	5.58E-13	-12.25	up	yes
ENSMUSG00000059336	Slc14a1	solute carrier	4.81	7.73	7.07	7.59	2.99	3.05	3.30	2.18	1.13	1.10E-15	8.09E-13	-12.09	up	yes
ENSMUSG00000058715	Fcer1g	Fc receptor, Ig	12.01	21.73	17.36	21.05	6.89	6.97	6.67	2.63	1.40	1.27E-15	9.10E-13	-12.04	up	yes
ENSMUSG00000079419	Ms4a6c	membrane-spanni	0.76	1.10	2.11	2.24	0.15	0.14	0.23	9.07	3.18	2.07E-15	1.45E-12	-11.84	up	yes
ENSMUSG00000039013	Siglecf	sialic acid bin	0.72	1.20	0.83	0.76	0.13	0.14	0.18	5.90	2.56	2.80E-15	1.92E-12	-11.72	up	yes
ENSMUSG00000055541	Lair1	leukocyte-assoc	1.84	2.46	2.75	2.59	1.17	1.14	1.17	2.08	1.05	2.84E-15	1.92E-12	-11.72	up	yes
ENSMUSG00000041559	Fmod	fibromodulin [S	11.59	12.72	13.23	14.18	8.15	7.01	6.35	1.80	0.85	7.03E-15	4.60E-12	-11.34	up	no
ENSMUSG00000040552	C3ar1	complement comp	1.38	2.63	3.31	3.17	0.81	0.73	0.69	3.54	1.82	7.08E-15	4.60E-12	-11.34	up	yes
ENSMUSG00000041607	Mbp	myelin basic pr	338.77	386.47	380.50	365.42	250.74	288.66	256.33	1.39	0.47	9.33E-15	5.94E-12	-11.23	up	no
ENSMUSG00000095562	Erdr1	erythroid diffe	4.23	3.34	3.00	3.84	14.44	10.26	8.01	0.33	-1.60	1.98E-14	1.23E-11	-10.91	down	yes
ENSMUSG00000004707	Ly9	lymphocyte anti	0.82	1.99	1.90	2.59	0.39	0.35	0.35	5.01	2.33	2.27E-14	1.39E-11	-10.86	up	yes
ENSMUSG00000072893	4933439C10R	RIKEN cDNA 4933	1.25	1.40	1.24	1.25	2.71	2.79	2.30	0.49	-1.02	2.50E-14	1.51E-11	-10.82	down	yes
ENSMUSG00000024621	Csf1r	colony stimulat	23.31	29.46	30.45	33.43	17.25	17.46	17.83	1.67	0.74	2.70E-14	1.60E-11	-10.80	up	no
ENSMUSG00000022037	Clu	clusterin [Sour	299.98	396.41	381.66	409.46	226.04	247.57	232.85	1.58	0.66	3.01E-14	1.75E-11	-10.76	up	no
ENSMUSG00000000290	Itgb2	integrin beta 2	3.88	7.93	7.77	9.15	2.58	2.63	2.12	2.94	1.56	4.10E-14	2.35E-11	-10.63	up	yes
ENSMUSG00000104415	Gm37069	predicted gene,	1.33	1.34	1.53	1.22	0.51	0.35	0.53	2.93	1.55	5.44E-14	3.02E-11	-10.52	up	yes
ENSMUSG00000044468	Tent5c	terminal nucleo	1.19	1.80	1.92	2.29	0.57	0.78	0.53	2.89	1.53	5.46E-14	3.02E-11	-10.52	up	yes
ENSMUSG00000004266	Ptpn6	protein tyrosin	2.45	4.35	3.85	4.62	1.42	1.38	1.40	2.73	1.45	6.88E-14	3.74E-11	-10.43	up	yes
ENSMUSG00000029723	Tsc22d4	TSC22 domain fa	9.86	9.90	10.27	12.03	6.54	6.87	5.92	1.63	0.71	7.18E-14	3.85E-11	-10.42	up	no
ENSMUSG00000044827	Tlr1	toll-like recep	0.54	1.04	0.77	0.79	0.14	0.13	0.19	5.21	2.38	1.15E-13	6.08E-11	-10.22	up	yes
ENSMUSG00000027447	Cst3	cystatin C [Sou	707.51	738.18	701.10	897.14	488.31	481.70	445.47	1.61	0.69	1.50E-13	7.79E-11	-10.11	up	no
ENSMUSG00000023067	Cdkn1a	cyclin-dependen	5.34	10.08	11.06	10.08	3.66	3.71	3.94	2.42	1.28	1.76E-13	8.99E-11	-10.05	up	yes
ENSMUSG00000015947	Fcgr1	Fc receptor, Ig	2.28	2.75	3.27	3.27	1.29	1.11	1.05	2.51	1.33	2.02E-13	1.02E-10	-9.99	up	yes
ENSMUSG00000004730	Adgre1	adhesion G prot	3.75	7.03	5.96	7.45	2.25	2.04	2.65	2.61	1.39	2.15E-13	1.07E-10	-9.97	up	yes
ENSMUSG00000031610	Scrg1	scrapie respons	22.87	24.01	24.02	25.25	13.60	12.80	14.85	1.75	0.81	2.39E-13	1.17E-10	-9.93	up	no
ENSMUSG00000040569	Slc26a7	solute carrier	0.83	1.45	1.20	1.56	0.41	0.45	0.38	3.03	1.60	2.50E-13	1.21E-10	-9.92	up	yes
ENSMUSG00000020143	Dock2	dedicator of cy	0.61	1.05	1.05	1.02	0.33	0.29	0.41	2.72	1.44	3.32E-13	1.58E-10	-9.80	up	yes
ENSMUSG00000026424	Gpr37l1	G protein-coupl	94.26	115.55	104.77	117.16	72.29	76.05	68.83	1.49	0.58	5.18E-13	2.43E-10	-9.61	up	no
ENSMUSG00000026701	Prdx6	peroxiredoxin 6	52.35	67.36	74.12	81.81	39.47	40.77	40.08	1.72	0.78	5.58E-13	2.58E-10	-9.59	up	no
ENSMUSG00000028581	Laptm5	lysosomal-assoc	13.44	19.15	19.33	20.85	10.29	9.73	10.31	1.80	0.85	7.04E-13	3.21E-10	-9.49	up	no
ENSMUSG00000004110	Cacna1e	calcium channel	5.72	5.44	5.74	4.95	8.81	8.37	7.99	0.65	-0.62	1.19E-12	5.37E-10	-9.27	down	no
ENSMUSG00000002602	Axl	AXL receptor ty	12.61	15.11	17.33	17.13	9.58	9.99	9.36	1.61	0.69	1.32E-12	5.85E-10	-9.23	up	no
ENSMUSG00000026177	Slc11a1	solute carrier	1.36	2.80	2.54	3.41	0.83	0.79	0.69	3.28	1.71	1.47E-12	6.42E-10	-9.19	up	yes
ENSMUSG00000042613	Pbxip1	pre B cell leuk	14.64	18.01	17.12	19.54	10.73	11.13	11.37	1.56	0.65	1.50E-12	6.47E-10	-9.19	up	no
ENSMUSG00000053279	Aldh1a1	aldehyde dehydr	17.64	24.85	24.10	28.07	10.89	15.07	12.35	1.85	0.89	1.68E-12	7.16E-10	-9.14	up	no
ENSMUSG00000051439	Cd14	CD14 antigen [S	2.66	3.54	4.10	5.29	1.43	1.23	0.93	3.25	1.70	1.74E-12	7.34E-10	-9.13	up	yes
ENSMUSG00000025351	Cd63	CD63 antigen [S	19.95	35.28	32.02	37.41	14.55	17.08	13.83	2.06	1.04	1.84E-12	7.68E-10	-9.11	up	yes
ENSMUSG00000021624	Cd180	CD180 antigen [	0.97	1.85	2.00	1.76	0.56	0.59	0.54	2.92	1.55	2.28E-12	9.40E-10	-9.03	up	yes
ENSMUSG00000034708	Grn	granulin [Sourc	23.28	27.61	29.38	35.17	16.99	17.62	16.07	1.71	0.77	2.90E-12	1.18E-09	-8.93	up	no
ENSMUSG00000037625	Cldn11	claudin 11 [Sou	64.31	67.97	67.03	76.49	45.08	50.09	41.05	1.52	0.60	3.01E-12	1.21E-09	-8.92	up	no
ENSMUSG00000027995	Tlr2	toll-like recep	1.30	3.43	2.31	3.73	0.31	0.72	0.71	4.63	2.21	3.16E-12	1.26E-09	-8.90	up	yes
ENSMUSG00000029622	Arpc1b	actin related p	3.40	5.00	5.26	7.04	2.17	2.21	2.35	2.31	1.21	3.82E-12	1.50E-09	-8.82	up	yes
ENSMUSG00000038147	Cd84	CD84 antigen [S	1.00	2.21	2.40	2.66	0.72	0.63	0.46	3.42	1.77	4.05E-12	1.57E-09	-8.80	up	yes
ENSMUSG00000006519	Cyba	cytochrome b-24	7.44	13.90	12.84	14.90	5.17	4.76	3.68	2.70	1.44	4.44E-12	1.70E-09	-8.77	up	yes
ENSMUSG00000032589	Bsn	bassoon [Source	12.66	10.67	10.86	10.39	18.94	17.28	18.27	0.61	-0.70	4.67E-12	1.77E-09	-8.75	down	no
ENSMUSG00000036570	Fxyd1	FXYD domain-con	11.57	15.77	16.73	18.96	8.89	7.91	6.79	2.00	1.00	4.73E-12	1.77E-09	-8.75	up	yes
ENSMUSG00000017760	Ctsa	cathepsin A [So	27.17	33.70	33.74	35.69	22.00	22.42	20.45	1.51	0.59	5.40E-12	2.00E-09	-8.70	up	no
ENSMUSG00000031447	Lamp1	lysosomal-assoc	145.06	170.63	174.48	177.81	115.13	125.53	119.52	1.39	0.48	5.51E-12	2.02E-09	-8.69	up	no
ENSMUSG00000040264	Gbp2b	guanylate bindi	0.42	1.31	1.73	1.65	0	0.02	0	185.94	7.54	6.49E-12	2.36E-09	-8.63	up	yes
ENSMUSG00000097961	Gm27000	predicted gene,	10.12	12.40	11.82	10.45	23.04	22.59	18.04	0.53	-0.92	7.98E-12	2.86E-09	-8.54	down	no

ENSMUSG00000026285	Pdcd1	programmed cell	0.57	0.85	0.88	0.69	0.14	0.06	0.10	7.56	2.92	1.17E-11	4.17E-09	-8.38 up	yes
ENSMUSG00000022488	Nckap1l	NCK associated	3.19	4.43	4.86	4.91	1.99	2.32	2.52	1.91	0.93	1.21E-11	4.24E-09	-8.37 up	no
ENSMUSG00000081229	Lamr1-ps1	laminin recepto	1.59	0.92	0.76	1.19	0	0	0	#######	13.44	1.29E-11	4.48E-09	-8.35 up	yes
ENSMUSG00000035805	Mlc1	megalencephalic	67.73	87.48	89.19	94.20	51.69	54.14	58.25	1.55	0.63	1.44E-11	4.97E-09	-8.30 up	no
ENSMUSG00000000982	Ccl3	chemokine (C-C	1.86	4.18	3.08	3.42	0.47	0.34	0.79	5.84	2.55	1.58E-11	5.40E-09	-8.27 up	yes

Table S2I: Selection of genes altered in PS/E2H vs E2H mice. FPKM values of genes in individual mice (indicated in underscored numeral), along with fold change (fc) value, p value adjusted for false discovery rate of 0.05 (q value, qval) are presented. N=3-4 mice/group. PE2H, PS19 mice homozygous for APOE2.

gene_id	gene_name	Description	FPKM. PE2H_1	FPKM. PE2H_2	FPKM. PE2H_3	FPKM. PE2H_4	FPKM. E2H_1	FPKM. E2H_2	FPKM. E2H_3	fc	log2 (fc)	pval	qval	log10 (qval)	regulation	significant
ENSMUSG00000033880	Lgals3bp	lectin, galactc	32.25	23.66	31.52	32.74	7.32	7.37	8.60	3.87	1.95	4.87E-45	1.61E-40	-39.79	up	yes
ENSMUSG00000020932	Gfap	glial fibrillar	252.75	261.69	365.94	273.41	42.93	67.11	61.24	5.05	2.34	1.10E-42	1.82E-38	-37.74	up	yes
ENSMUSG00000030789	Itgax	integrin alpha	1.96	3.15	4.25	1.76	0.15	0.23	0.20	14.30	3.84	6.24E-39	6.87E-35	-34.16	up	yes
ENSMUSG00000073418	C4b	complement comp	39.70	30.52	49.85	32.65	9.95	7.89	10.05	4.11	2.04	8.60E-39	7.10E-35	-34.15	up	yes
ENSMUSG00000021091	Serpina3n	serine (or cyst	51.49	45.44	85.65	81.03	14.64	13.27	12.50	4.89	2.29	3.22E-32	2.13E-28	-27.67	up	yes
ENSMUSG00000036887	C1qa	complement comp	125.64	136.57	133.80	123.41	45.62	53.76	41.80	2.76	1.46	4.39E-30	2.41E-26	-25.62	up	yes
ENSMUSG00000034708	Grn	granulin [Sourc	29.10	30.87	34.83	31.64	13.64	14.24	15.23	2.20	1.14	8.59E-27	4.05E-23	-22.39	up	yes
ENSMUSG00000029561	Oasl2	2'-5' oligoader	3.70	2.32	2.60	4.05	0.64	0.52	0.81	4.84	2.28	3.16E-25	1.30E-21	-20.88	up	yes
ENSMUSG00000035692	Isg15	ISG15 ubiquitir	10.13	10.17	7.63	8.89	2.20	2.11	1.95	4.41	2.14	1.42E-23	5.22E-20	-19.28	up	yes
ENSMUSG00000061232	H2-K1	histocompatibil	24.84	24.49	25.95	21.13	9.36	10.54	11.78	2.28	1.19	4.13E-21	1.36E-17	-16.87	up	yes
ENSMUSG00000073411	H2-D1	histocompatibil	50.16	50.31	53.53	50.11	16.75	23.67	21.27	2.48	1.31	5.43E-21	1.63E-17	-16.79	up	yes
ENSMUSG00000030124	Lag3	lymphocyte-acti	13.34	18.75	12.50	11.25	2.47	4.44	3.28	4.11	2.04	9.85E-20	2.71E-16	-15.57	up	yes
ENSMUSG00000036905	C1qb	complement comp	113.91	127.81	121.42	106.91	49.08	56.85	42.49	2.38	1.25	1.39E-19	3.54E-16	-15.45	up	yes
ENSMUSG00000079037	Prnp	prion protein [	690.35	734.63	669.32	707.99	345.76	385.49	364.74	1.92	0.94	6.87E-19	1.62E-15	-14.79	up	no
ENSMUSG00000058626	Capn11	calpain 11 [Sou	0.13	0.06	0.10	0.06	0.82	1.23	0.98	0.09	-3.53	7.90E-19	1.74E-15	-14.76	down	yes
ENSMUSG00000036896	C1qc	complement comp	52.66	52.71	59.19	44.44	19.99	25.24	20.46	2.39	1.25	1.49E-18	3.07E-15	-14.51	up	yes
ENSMUSG00000074896	Ifit3	interferon-indu	20.83	18.22	18.94	14.20	5.50	7.11	7.15	2.74	1.45	4.34E-18	8.42E-15	-14.07	up	yes
ENSMUSG00000040264	Gbp2b	guanylate bindi	1.04	1.21	2.01	0.66	0	0	0	12308.52	13.59	2.86E-17	5.25E-14	-13.28	up	yes
ENSMUSG00000028037	Ifi44	interferon-indu	1.09	0.60	0.86	1.03	0.07	0.19	0.12	7.02	2.81	3.25E-17	5.65E-14	-13.25	up	yes
ENSMUSG00000098374	Gm28043	predicted gene,	5.40	4.54	4.33	4.03	1.88	1.13	1.88	2.80	1.49	3.57E-17	5.89E-14	-13.23	up	yes
ENSMUSG00000038642	Ctss	cathepsin S [Sc	146.02	141.30	155.61	131.80	63.36	77.99	59.36	2.15	1.10	3.37E-16	5.31E-13	-12.28	up	yes
ENSMUSG00000024610	Cd74	CD74 antigen (i	9.73	16.74	15.61	11.12	2.27	4.46	3.41	3.93	1.98	5.41E-16	8.13E-13	-12.09	up	yes
ENSMUSG00000030107	Usp18	ubiquitin speci	2.52	1.58	2.33	1.77	0.43	0.58	0.49	4.10	2.04	8.84E-16	1.27E-12	-11.90	up	yes
ENSMUSG00000035929	H2-Q4	histocompatibil	3.68	3.70	4.25	3.55	0.94	1.43	1.41	3.01	1.59	1.13E-15	1.56E-12	-11.81	up	yes
ENSMUSG00000028341	Nr4a3	nuclear recepto	2.24	2.38	2.21	2.76	4.66	4.64	5.72	0.48	-1.06	5.62E-15	7.42E-12	-11.13	down	yes
ENSMUSG00000007891	Ctsd	cathepsin D [Sc	381.31	442.15	531.74	359.94	185.13	216.04	179.13	2.22	1.15	6.01E-15	7.64E-12	-11.12	up	yes
ENSMUSG00000015950	Ncf1	neutrophil cytc	4.12	4.27	4.48	3.62	1.95	1.93	1.86	2.16	1.11	8.19E-15	1.00E-11	-11.00	up	yes
ENSMUSG00000040552	C3ar1	complement comp	1.93	1.61	2.14	1.50	0.71	0.59	0.55	2.92	1.55	1.96E-14	2.31E-11	-10.64	up	yes
ENSMUSG00000046718	Bst2	bone marrow str	11.97	10.45	9.21	15.75	3.26	3.13	4.55	3.25	1.70	4.51E-14	5.13E-11	-10.29	up	yes
ENSMUSG00000073489	Ifi204	interferon acti	1.01	0.56	0.90	1.00	0.16	0.20	0.12	5.53	2.47	2.84E-13	3.12E-10	-9.51	up	yes
ENSMUSG00000079293	Clec7a	C-type lectin c	5.14	4.14	10.20	1.87	0.31	0.28	0.28	18.36	4.20	3.15E-13	3.36E-10	-9.47	up	yes
ENSMUSG00000039146	Ifi44l	interferon-indu	0.85	1.04	1.12	0.79	0	0	0.07	42.18	5.40	3.45E-13	3.57E-10	-9.45	up	yes
ENSMUSG00000038147	Cd84	CD84 antigen [S	1.69	1.23	2.20	1.34	0.57	0.54	0.54	2.95	1.56	4.70E-13	4.71E-10	-9.33	up	yes
ENSMUSG00000018927	Ccl6	chemokine (C-C	4.85	2.99	6.10	2.89	0.83	1.07	1.18	4.11	2.04	5.13E-13	4.98E-10	-9.30	up	yes
ENSMUSG00000110631	Gm42047	predicted gene,	1.12	0.94	1.14	0.81	0.28	0.22	0.40	3.33	1.74	8.25E-13	7.79E-10	-9.11	up	yes
ENSMUSG00000081229	Lamr1-ps1	laminin recepto	1.20	1.30	1.11	1.41	0	0	0	12539.39	13.61	2.61E-12	2.39E-09	-8.62	up	yes
ENSMUSG00000042613	Pbxip1	pre B cell leuk	17.06	16.65	19.25	17.75	9.94	10.47	11.15	1.68	0.75	2.69E-12	2.40E-09	-8.62	up	no
ENSMUSG00000025492	Ifitm3	interferon indu	31.56	30.35	27.14	36.61	15.45	15.53	14.72	2.06	1.04	4.25E-12	3.69E-09	-8.43	up	yes
ENSMUSG00000068129	Cst7	cystatin F (leu	7.90	10.09	18.97	8.18	0.23	1.04	0.60	18.14	4.18	8.96E-12	7.59E-09	-8.12	up	yes
ENSMUSG00000028268	Gbp3	guanylate bindi	3.25	2.67	3.63	1.92	0.79	0.74	1.19	3.16	1.66	9.68E-12	8.00E-09	-8.10	up	yes
ENSMUSG00000024529	Lox	lysyl oxidase [	0.49	0.39	0.65	0.42	0.09	0.06	0.12	5.47	2.45	1.11E-11	8.97E-09	-8.05	up	yes
ENSMUSG00000051439	Cd14	CD14 antigen [S	3.43	3.09	4.75	4.30	1.67	1.02	1.06	3.12	1.64	1.20E-11	9.44E-09	-8.02	up	yes
ENSMUSG00000004707	Ly9	lymphocyte anti	1.45	0.91	2.44	0.97	0.27	0.34	0.21	5.27	2.40	1.38E-11	1.06E-08	-7.97	up	yes
ENSMUSG00000056737	Capg	capping proteir	2.87	2.74	3.51	2.53	1.18	1.26	1.19	2.41	1.27	1.51E-11	1.14E-08	-7.94	up	yes
ENSMUSG00000034459	Ifit1	interferon-indu	4.22	2.24	4.00	2.48	0.71	0.92	1.18	3.45	1.79	2.31E-11	1.68E-08	-7.77	up	yes
ENSMUSG00000108815	Gm49388	predicted gene,	0	0	0	0	1.70	0	0	0.00	-12.47	2.38E-11	1.68E-08	-7.77	down	yes
ENSMUSG00000029622	Arpc1b	actin related p	4.66	4.23	5.28	4.32	2.76	2.30	2.42	1.85	0.89	2.39E-11	1.68E-08	-7.77	up	no
ENSMUSG00000094685	Gm5900	predicted pseuc	5.84	2.53	6.10	3.86	0.70	0.59	0.45	7.90	2.98	4.35E-11	2.99E-08	-7.52	up	yes
ENSMUSG00000107705	Gm45062	predicted gene	0	0	0	0	0	0.86	0	0.00	-11.49	5.82E-11	3.92E-08	-7.41	down	yes
ENSMUSG00000093938	Evi2b	ecotropic viral	1.15	0.58	0.11	0.61	0	0	0	6119.77	12.58	6.43E-11	4.24E-08	-7.37	up	yes
ENSMUSG00000030577	Cd22	CD22 antigen [S	0.77	0.39	0.75	0.56	0.04	0.14	0.10	6.53	2.71	8.51E-11	5.51E-08	-7.26	up	yes
ENSMUSG00000032359	Ctsh	cathepsin H [Sc	5.32	5.48	5.84	4.36	2.56	2.94	2.57	1.95	0.96	1.21E-10	7.66E-08	-7.12	up	no
ENSMUSG00000033355	Rtp4	receptor transp	3.22	2.41	2.96	3.60	1.33	1.07	1.21	2.53	1.34	1.30E-10	8.11E-08	-7.09	up	yes
ENSMUSG00000033777	Tlr13	toll-like recep	1.09	1.22	1.35	1.58	0.52	0.51	0.38	2.79	1.48	1.41E-10	8.63E-08	-7.06	up	yes
ENSMUSG00000026728	Vim	vimentin [Sourc	15.63	15.90	32.01	18.54	7.85	8.19	8.67	2.49	1.32	1.63E-10	9.76E-08	-7.01	up	yes
ENSMUSG00000024737	Slc15a3	solute carrier	1.31	0.98	1.40	0.71	0.29	0.40	0.27	3.44	1.78	1.84E-10	1.09E-07	-6.96	up	yes
ENSMUSG00000036103	Colec12	collectin sub-f	1.95	2.01	2.15	2.40	0.81	0.95	1.24	2.13	1.09	2.92E-10	1.69E-07	-6.77	up	yes
ENSMUSG00000005142	Man2b1	mannosidase 2,	10.11	12.01	10.69	8.74	5.34	5.94	6.07	1.80	0.84	3.14E-10	1.79E-07	-6.75	up	no
ENSMUSG00000026104	Stat1	signal transduc	6.69	4.95	6.97	5.86	3.52	3.09	3.43	1.83	0.87	3.57E-10	2.00E-07	-6.70	up	no
ENSMUSG00000068457	Uty	ubiquitously tr	0.01	0.88	0	0	0	0	0	2207.08	11.11	3.65E-10	2.01E-07	-6.70	up	yes
ENSMUSG00000027015	Cybrd1	cytochrome b re	0.85	0.73	1.06	1.00	0.34	0.33	0.36	2.64	1.40	3.92E-10	2.12E-07	-6.67	up	yes
ENSMUSG00000036594	H2-Aa	histocompatibil	1.37	2.37	3.04	1.57	0.54	0.70	0.67	3.28	1.71	4.40E-10	2.34E-07	-6.63	up	yes
ENSMUSG00000022218	Tgm1	transglutaminas	0.27	0.52	1.66	0.11	0.02	0.03	0.03	25.59	4.68	5.22E-10	2.73E-07	-6.56	up	yes
ENSMUSG00000097415	AU020206	expressed seque	3.82	3.23	4.07	2.50	1.36	1.30	1.68	2.35	1.23	5.77E-10	2.98E-07	-6.53	up	yes
ENSMUSG00000089865	Gm44503	predicted readt	0	0	0	0	0	0.44	0	0.00	-10.53	6.13E-10	3.11E-07	-6.51	down	yes
ENSMUSG00000033685	Ucp2	uncoupling prot	6.10	6.80	7.22	10.17	3.59	3.84	3.05	2.17	1.12	6.62E-10	3.31E-07	-6.48	up	yes
ENSMUSG00000020681	Ace	angiotensin I c	7.76	7.34	7.81	8.55	4.91	4.70	4.98	1.62	0.69	6.98E-10	3.44E-07	-6.46	up	no
ENSMUSG00000026656	Fcgr2b	Fc receptor, Ig	2.21	1.20	3.49	2.67	0.80	0.77	0.69	3.19	1.67	7.47E-10	3.63E-07	-6.44	up	yes
ENSMUSG00000037321	Tap1	transporter 1,	1.64	1.25	1.84	1.39	0.56	0.64	0.75	2.35	1.24	7.67E-10	3.67E-07	-6.44	up	yes
ENSMUSG00000022436	Sh3bp1	SH3-domain binc	3.69	3.48	2.96	5.42	1.59	1.45	1.61	2.51	1.33	1.17E-09	5.53E-07	-6.26	up	yes
ENSMUSG00000064267	Hvcn1	hydrogen voltag	1.58	1.74	1.62	1.44	0.50	0.74	0.64	2.55	1.35	1.29E-09	5.99E-07	-6.22	up	yes
ENSMUSG00000016256	Ctsz	cathepsin Z [Sc	56.85	66.63	71.01	46.83	28.53	34.89	29.37	1.95	0.96	1.42E-09	6.51E-07	-6.19	up	no
ENSMUSG00000000982	Ccl3	chemokine (C-C	2.43	1.77	3.39	1.66	0.48	0.40	0.21	6.37	2.67	1.50E-09	6.79E-07	-6.17	up	yes
ENSMUSG00000019122	Ccl9	chemokine (C-C	1.11	1.59	1.22	1.30	0.32	0.44	0.53	3.03	1.60	2.13E-09	9.49E-07	-6.02	up	yes
ENSMUSG00000025498	Irf7	interferon regu	2.59	1.72	1.92	3.08	0.27	0.54	0.96	3.94	1.98	2.65E-09	1.16E-06	-5.93	up	yes
ENSMUSG00000060802	B2m	beta-2 microglc	241.27	192.64	245.92	182.24	101.24	132.09	111.34	1.88	0.91	3.08E-09	1.34E-06	-5.87	up	no
ENSMUSG00000004328	Hif3a	hypoxia inducit	1.28	1.44	1.78	2.60	0.73	0.34	0.67	3.07	1.62	3.23E-09	1.38E-06	-5.86	up	yes
ENSMUSG00000018774	Cd68	CD68 antigen [S	20.19	21.39	29.92	14.82	7.34	10.87	7.80	2.49	1.32	3.71E-09	1.57E-06	-5.80	up	yes
ENSMUSG00000028480	Glipr2	GLI pathogenesi	3.00	2.56	2.88	4.03	1.33	1.39	1.08	2.46	1.30	4.43E-09	1.85E-06	-5.73	up	yes
ENSMUSG00000045294	Insig1	insulin inducec	14.19	15.24	15.02	12.18	21.86	20.27	24.41	0.64	-0.65	5.21E-09	2.15E-06	-5.67	down	no
ENSMUSG00000063415	Cyp26b1	cytochrome P45C	2.92	2.41	3.19	2.82	0.97	1.42	1.53	2.17	1.12	5.28E-09	2.15E-06	-5.67	up	yes
ENSMUSG00000028859	Csf3r	colony stimulat	3.06	2.86	4.68	3.07	1.54	1.16	1.76	2.30	1.20	6.01E-09	2.42E-06	-5.62	up	yes
ENSMUSG00000022901	Cd86	CD86 antigen [S	1.74	1.39	1.90	1.15	0.48	0.55	0.70	2.68	1.42	6.16E-09	2.45E-06	-5.61	up	yes
ENSMUSG00000030921	Trim30a	tripartite moti	1.69	1.42	2.30	1.21	0.69	0.62	0.58	2.63	1.39	6.38E-09	2.51E-06	-5.60	up	yes
ENSMUSG00000071648	Rom1	rod outer segme	5.04	5.01	5.16	5.60	2.63	2.73	2.71	1.93	0.95	7.66E-09	2.98E-06	-5.53	up	no
ENSMUSG00000050359	Sprr1a	small proline-r	0.97	0.91	0.80	0.85	0.05	0.07	0	22.15	4.47	7.95E-09	3.05E-06	-5.52	up	yes
ENSMUSG00000113342	AA414992	expressed seque	0.25	0.26	0.43	0.29	1.17	1.40	1.19	0.25	-2.02	9.69E-09	3.68E-06	-5.43	down	yes
ENSMUSG00000020101	Vsir	V-set immunoreg	7.74	7.62	8.41	7.42	5.01	4.87	5.35	1.54	0.62	9.87E-09	3.70E-06	-5.43	up	no
ENSMUSG00000024965	Fermt3	fermitin family	1.24	1.41	1.61	1.25	0.63	0.65	0.75	2.03	1.02	1.07E-08	3.96E-06	-5.40	up	yes
ENSMUSG00000038059	Smim3	small integral	1.45	1.54	1.51	1.91	0.76	0.79	0.89	1.97	0.98	1.11E-08	4.07E-06	-5.39	up	no
ENSMUSG00000024621	Csf1r	colony stimulat	31.97	29.95	31.25	23.54	16.25	18.46	15.44	1.75	0.80	1.18E-08	4.30E-06	-5.37	up	no
ENSMUSG00000017009	Sdc4	syndecan 4 [Sou	36.28	37.97	41.42	49.64	24.76	24.90	21.31	1.75	0.80	1.26E-08	4.51E-06	-5.35	up	no
ENSMUSG00000023992	Trem2	triggering rece	6.21	6.87	6.88	4.36	2.09	3.41	2.54	2.27	1.18	1.38E-08	4.85E-06	-5.31	up	yes
ENSMUSG00000015340	Cybb	cytochrome b-24	0.47	0.39	1.18	0.41	0.09	0.09	0.17	5.15	2.37	1.38E-08	4.85E-06	-5.31	up	yes
ENSMUSG00000021886	Gpr65	G-protein coupl	0.53	0.54	1.08	1.77	0.06	0.10	0.21	8.06	3.01	1.51E-08	5.26E-06	-5.28	up	yes

ENSMUSG00000024079	Eif2ak2	eukaryotic trar	1.51	1.27	1.98	1.37	0.74	0.58	0.75	2.22	1.15	1.64E-08	5.63E-06	-5.25 up	yes
ENSMUSG00000052911	Lamb2	laminin, beta 2	5.67	5.78	6.55	4.44	2.97	3.15	3.35	1.78	0.83	1.81E-08	6.15E-06	-5.21 up	no
ENSMUSG00000046879	Irgm1	immunity-relate	3.18	2.47	3.29	2.95	1.25	1.53	1.79	1.95	0.96	2.02E-08	6.81E-06	-5.17 up	no
ENSMUSG00000000386	Mx1	MX dynamin-like	0.49	0.26	0.36	0.50	0.08	0.02	0.09	6.20	2.63	2.25E-08	7.50E-06	-5.13 up	yes
